# From Foreshock 30-Second Waves to Magnetospheric Pc3 Waves

**DOI:** 10.1007/s11214-025-01152-y

**Published:** 2025-03-07

**Authors:** Lucile Turc, Kazue Takahashi, Primož Kajdič, Emilia K. J. Kilpua, Theodoros Sarris, Minna Palmroth, Jan Soucek, Yann Pfau-Kempf, Andrew Dimmock, Naoko Takahashi

**Affiliations:** 1 Department of Physics, University of Helsinki, Helsinki, Finland; 2 The Johns Hopkins University Applied Physics Laboratory, Laurel, MD USA; 3 Departamento de Ciencias Espaciales, Instituto de Geofísica, Universidad Nacional Autónoma de México, Mexico City, Mexico; 4 Department of Electrical and Computer Engineering, Democritus University of Thrace, Xanthi, Greece; 5 Finnish Meteorological Institute, Helsinki, Finland; 6 Institute of Atmospheric Physics, Czech Academy of Sciences, Prague, Czech Republic; 7 Swedish Institute of Space Physics, Uppsala, Sweden; 8 Radio Research Institute, National Institute of Information and Communication Technology, Tokyo, Japan

## Abstract

Ultra-low frequency waves, with periods between 1-1000 s, are ubiquitous in the near-Earth plasma environment and play an important role in magnetospheric dynamics and in the transfer of electromagnetic energy from the solar wind to the magnetosphere. A class of those waves, often referred to as Pc3 waves when they are recorded from the ground, with periods between 10 and 45 s, are routinely observed in the dayside magnetosphere. They originate from the ion foreshock, a region of geospace extending upstream of the quasi-parallel portion of Earth’s bow shock. There, the interaction between shock-reflected ions and the incoming solar wind gives rise to a variety of waves, and predominantly fast-magnetosonic waves with a period typically around 30 s. The connection between these waves upstream of the shock and their counterparts observed inside the magnetosphere and on the ground was inferred already early on in space observations due to similar properties, thereby implying the transmission of the waves across near-Earth space, through the shock and the magnetopause. This review provides an overview of foreshock 30-second/Pc3 waves research from the early observations in the 1960s to the present day, covering the entire propagation pathway of these waves, from the foreshock to the ground. We describe the processes at play in the different regions of geospace, and review observational, theoretical and numerical works pertaining to the study of these waves. We conclude this review with unresolved questions and upcoming opportunities in both observations and simulations to further our understanding of these waves.

## Introduction

**Fig. 1:**
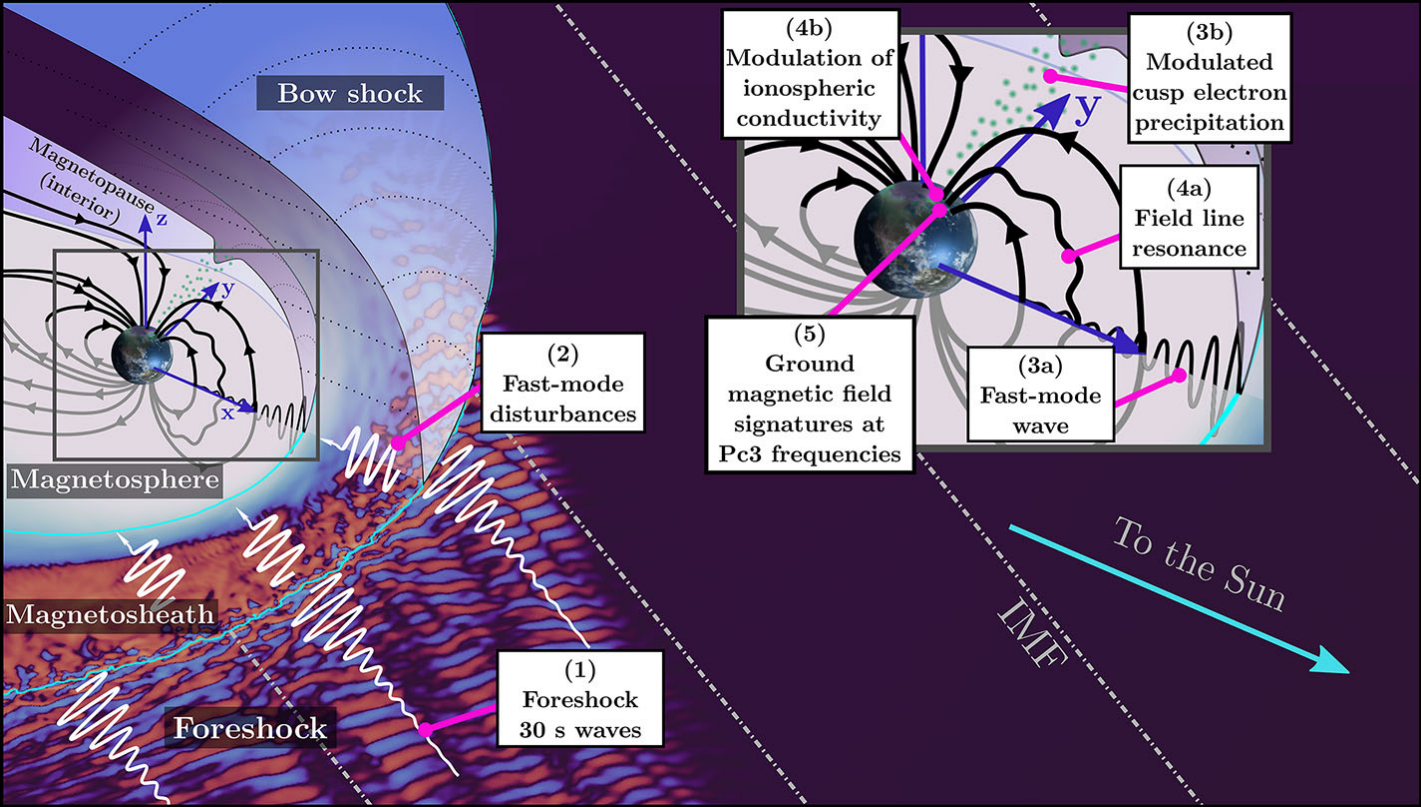
Overview of near-Earth space, with the main steps of the transmission pathways of foreshock 30-second waves: (1) generation in the foreshock, (2) transmission through the magnetosheath, (3) entry into the magnetosphere in the equatorial region as fast-mode waves (3a) coupling with field line resonances (4a) and at high latitudes via modulated precipitation (3b) resulting in variations of the ionospheric conductivity (4b), eventually leading to (5) the observations of Pc3 pulsations on the ground

The Earth’s magnetosphere, the region of space dominated by our planet’s magnetic field, acts as a magnetic obstacle to the solar wind plasma streaming through our solar system. Because this flow is supermagnetosonic, a standing bow shock forms ahead of the magnetosphere to slow down and deflect the solar wind. Downstream of the bow shock lies the magnetosheath, a transition layer filled with heated and compressed solar wind plasma and magnetic field, separating the upstream solar wind from the magnetosphere (see Fig. 1 for an overview of these different regions of near-Earth space).

For most solar wind conditions encountered at Earth, the terrestrial bow shock is supercritical, meaning that a fraction of the solar wind particles are reflected back into the upstream in order to provide efficient energy dissipation for the flow to become submagnetosonic upon crossing the shock (e.g. Burgess et al. 2012). At the quasi-perpendicular shock, where the angle $\theta _{\mathrm{Bn}}$ between the shock normal and the interplanetary magnetic field (IMF) is greater than $45^{\circ}$, these reflected particles return to the bow shock within a few gyrations and rapidly cross into the downstream. As a result, the shock front appears as an abrupt and well-defined transition between the solar wind and the magnetosheath (Bale et al. 2005; Burgess et al. 2012). In contrast, at the quasi-parallel shock, where $\theta _{\mathrm{Bn}} < 45^{\circ}$, shock-reflected particles can stream back far into the upstream, forming an extended foreshock which can be observed as far as the Moon’s orbit ($\sim 60~R_{\mathrm{E}}$) (Eastwood et al. 2005c; Burgess et al. 2005, 2012; Wilson 2016). The quasi-parallel shock transition extends over many ion gyroradii, and the exact shock position often cannot be determined unambiguously (e.g. Schwartz and Burgess 1991; Lucek et al. 2008; Battarbee et al. 2020). The foreshock is divided into an electron foreshock, containing only backstreaming electrons, and an ion foreshock, where both ions and electrons are found. As this review is solely concerned with processes occurring in the ion foreshock, we will hereafter refer to the ion foreshock simply as the foreshock.

The Earth’s foreshock is permeated with a variety of waves in the Ultra-Low Frequency (ULF) range ($1 \mathrm{mHz} - 1$ Hz, below the ion gyrofrequency), generated through instabilities caused by the backstreaming particles reflected at the bow shock (Greenstadt et al. 1995; Eastwood et al. 2005c; Wilson 2016). In this review, we will focus on the “30-second” waves, which are the dominant type of waves observed in Earth’s foreshock (Eastwood et al. 2005a). These waves are quasi-monochromatic, circularly polarised, fast-magnetosonic waves, with their typical period around 30 s (Fairfield 1969; Eastwood et al. 2005b; Wilson 2016). Although these waves propagate sunward in the plasma rest frame, they are carried earthward by the faster solar wind flow, towards the bow shock and the magnetosphere.

It has been inferred already early on that foreshock 30-second waves could be an important source of geomagnetic pulsations in the same frequency range, termed Pc3 ($10 - 45$ s or $22 - 100$ mHz) in the International Association of Geomagnetism and Aeronomy (IAGA) classification of continuous pulsations (see Table 1 and Jacobs et al. 1964), in the dayside magnetosphere (Troitskaya et al. 1971; Greenstadt and Olson 1977; Russell et al. 1983; Engebretson et al. 1987). The frequencies of both foreshock 30-second and dayside Pc3 waves show the same dependency on the IMF magnitude (Russell and Hoppe 1981; Takahashi et al. 1984; Le and Russell 1996), and the occurrence of Pc3 waves in the magnetosphere is well-correlated with the IMF direction, and thus with the position of the foreshock (Wolfe et al. 1985; Yumoto et al. 1985; Odera 1986; Chi et al. 1994; Heilig et al. 2007; Bier et al. 2014). Dayside Pc3 wave power maximises for quasi-radial IMF orientations, when the foreshock is located upstream of the subsolar bow shock, and decreases as the foreshock retreats towards the flanks. These observations suggest that foreshock 30-second waves can survive in some form as they cross the bow shock and into the magnetosheath to eventually reach the outer boundary of the magnetosphere, the magnetopause. The transmission of foreshock waves across near-Earth space, and in particular through Earth’s bow shock and magnetosheath, is a major open problem in space physics. Considerable effort has also been put into unravelling the physical processes responsible for wave transmission and understanding the impact of solar wind conditions on the wave properties. Yet, many questions remain unresolved and this area of research is still very active to date.

**Table 1:** Classification of continuous pulsations. Based on Jacobs et al. (1964)

	Frequency range	Period range
Pc1	200 mHz – 5 Hz	0.2–5 s
Pc2	100–200 mHz	5–10 s
Pc3	22–100 mHz	10–45 s
Pc4	6.67–22 mHz	45–150 s
Pc5	1.67–6.67 mHz	150–600 s

This review aims at providing a comprehensive overview of our current understanding of 30-second/Pc3 waves and of their transmission from the foreshock to the ground. The main steps of their propagation pathways are illustrated in Fig. 1. These waves travel across geophysical regions with vastly differing properties and dynamics, which are generally studied separately and in which different terminologies are sometimes used. For example, foreshock 30-second waves are commonly referred to as “upstream waves” in magnetospheric studies, while the “Pc3” classification is not used in the foreshock community, as it specifically applies to magnetospheric waves. Here, we will bring together the different pieces of this complex puzzle to form a complete picture of the 30-second/Pc3 wave generation and transmission across near-Earth space and their impact on their environment.

In the past two decades, significant progress made on both the numerical and observational fronts has brought about considerable breakthroughs in our understanding of kinetic – ion and electron scale – processes in near-Earth space and their contribution to its global dynamics. On the one hand, satellite constellations such as Cluster (Escoubet et al. 2001), the Time History of Events and Macroscale Interactions during Substorms (THEMIS, Angelopoulos 2008) and the Magnetospheric Multiscale (MMS, Burch et al. 2016) missions have enabled the determination of wave properties with great accuracy through multi-spacecraft analysis techniques, resulting in a thorough characterisation of ULF waves in the foreshock, magnetosheath and magnetosphere (e.g., Eastwood et al. 2005a; Archer et al. 2005; Narita et al. 2006; Sarris et al. 2009c; Shi et al. 2020). On the other hand, the ever-increasing computing power has progressively allowed to run kinetic models with larger simulation domains and more realistic setups, providing a framework to study kinetic processes in their global context. Foreshock 30-second waves, born from ion beam instabilities, are an inherently kinetic phenomenon. Our understanding of these waves benefits largely from hybrid simulations (kinetic ions and fluid electrons) which are shedding new light on their properties and their propagation across near-Earth space (e.g., Lin and Wang 2005; Blanco-Cano et al. 2006, 2009; Palmroth et al. 2015; Shi et al. 2017; Turc et al. 2018, 2019; Takahashi et al. 2021; Shi et al. 2021; Turc et al. 2023). In this review, we will consider 30-second/Pc3 wave propagation through the lens of kinetic physics and discuss the importance of kinetic effects, in particular for wave transmission across boundaries such as the bow shock and the magnetopause.

Understanding foreshock dynamics is crucial in terms of solar wind-magnetospheric coupling. The focus of this review will be on foreshock waves transmitting into the magnetosphere, but we also note that transient foreshock structures can cause large-scale disturbances in Earth’s magnetosphere, including magnetopause motions and bursts of intense ULF wave activity (Jacobsen et al. 2009; Hartinger et al. 2013b; Shen et al. 2018; Wang et al. 2019). Furthermore, the processing of the solar wind across the shock is initiated already in the foreshock region, where the solar wind starts slowing down and being deflected (Cao et al. 2009; Gutynska et al. 2020). The foreshock is therefore an inherent and essential part of the near-Earth space system.

The organisation of this review follows the pathway of the 30-second/Pc3 waves on their earthward journey. We start in Sect. 2 with their source in the foreshock, their main properties and their impact on the quasi-parallel bow shock. We then discuss in Sect. 3 the transmission of fast-magnetosonic waves through shock waves, which has been predominantly studied theoretically and numerically. Section 4 continues with an overview of ULF wave activity in the magnetosheath in relation to the quasi-parallel bow shock and foreshock. Section 5 focuses on wave transmission through the magnetopause, while further propagation into the magnetosphere and coupling with standing waves through field-line resonances are described in Sect. 6. The impact of Pc3 waves on magnetospheric and ionospheric dynamics is presented in Sect. 7. The influence of solar wind parameters and changing upstream conditions is considered in Sect. 8. Finally, Sect. 9 discusses similar waves in other environments in our solar system and we conclude this review with an outlook for future research at Earth and beyond, making use of novel numerical and observational assets.

## Generation of 30-Second Waves in the Foreshock

In this section we provide a brief introduction to the foreshock, focusing on processes relevant for the generation of magnetospheric Pc3 pulsations. We first define different regions of the foreshock and the phenomena that reside in each of them, and then we describe the generation of foreshock waves and their evolution into new structures as a result of wave steepening when approaching the bow shock.

### The Foreshock Region: Suprathermal Populations and Waves Upstream of the Bow Shock

**Fig. 2:**
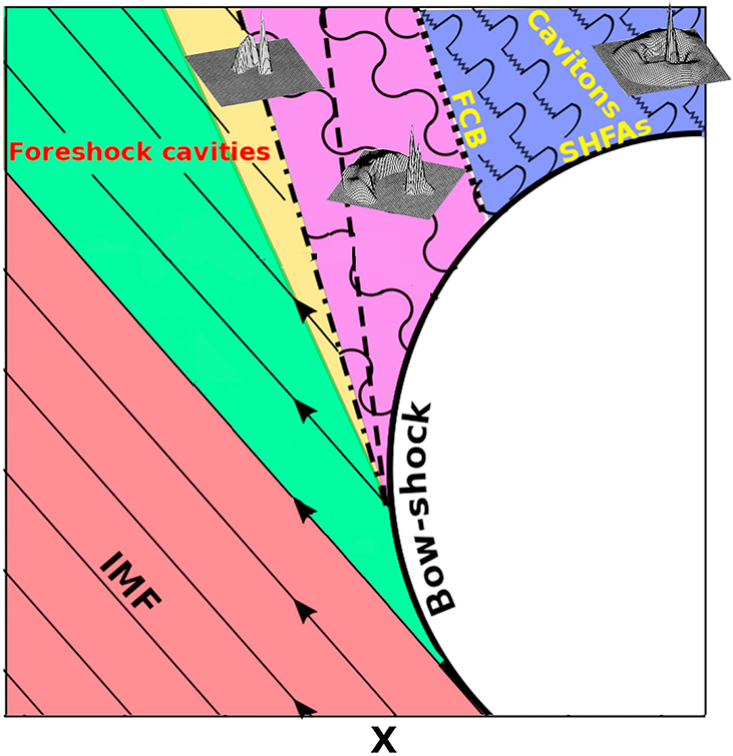
Regions in the near-Earth environment and associated ion populations. The green, yellow, purple and blue colors represent regions magnetically connected to the bow shock. The ion and ULF wave foreshocks are colored in purple and blue. Superposed are three panels exhibiting examples of field-aligned (Fairfield 1969; Paschmann et al. 1980), intermediate (Argo et al. 1967; Meziane and d’Uston 1998) and diffuse (Paschmann et al. 1981; Bonifazi and Moreno 1981a,b; Winske and Leroy 1984) ion distributions that coexist with narrow-peaked solar wind ion distributions. Adapted from Kajdič et al. (2017) and Paschmann et al. (1981)

Before discussing the generation and properties of 30-second waves, we first introduce the foreshock and the different particle populations and waves found in this region. The foreshock is a region located upstream of the bow shock of the Earth that is magnetically connected to the bow shock. It is in this region that the solar wind is first decelerated and thermalized (Cao et al. 2009; Gutynska et al. 2020). There are in fact different foreshock sections that receive names according to the phenomena (waves, particles) that populate them. These sections and phenomena are presented schematically in Fig. 2 (after Kajdič et al. 2017).

In this Figure, the Earth’s bow shock is represented by the thick black curve, while the straight and wavy thin black lines represent the IMF lines. In an idealized, steady situation, there exists a magnetic field line (or a surface when considering the 3D situation) that divides the region upstream of the bow shock into the region that is not magnetically connected to the shock (red color in Fig. 2) and the region where the IMF lines cross the bow shock. At the location where this tangential magnetic field line touches the shock, the $\theta _{\mathrm{Bn}}$ angle is equal to $90^{\circ}$, and the shock there is exactly perpendicular. When moving along the bow shock surface (upward and left in the Fig. 2), this angle diminishes and the shock becomes quasi-perpendicular ($45^{ \circ}<\theta _{\mathrm{Bn}}<90^{\circ}$).

Immediately downstream of the tangential line there is a region populated, in addition to the solar wind particles, by hot ($\sim 100$ eV) electrons that are reflected at the shock (Fredricks et al. 1968). This region is called the electron foreshock (green color). One type of ULF waves, called the upstream-propagating whistlers or 1 Hz waves, is often observed there (e.g., Russell et al. 1971; Hoppe and Russell 1981; Hoppe et al. 1982; Greenstadt et al. 1995). More ubiquitous are the higher-frequency, electrostatic Langmuir waves with frequencies equal to the local plasma frequency (e.g., Fuselier et al. 1985).

A small portion ($\lesssim 10\%$) of incident solar wind ions is also reflected at the quasi-perpendicular shock. These particles reach a maximum distance of ≲1 ion gyroradius upstream of the shock ramp and are then turned back towards it by the Lorentz force (e.g., Schwartz et al. 1983). Hence, the reflected ions are responsible for the formation of density and magnetic field magnitude increases just in front of the shock, known as the foot. The reflected ions may cross into the magnetosheath after just one reflection or they may do so after being reflected several times by the shock. They cannot, however, escape large distances from the bow shock. The solar wind upstream of the quasi-perpendicular shock is thus relatively calm in terms of any ULF wave activity (except for fluctuations already present in the solar wind itself).

Moving along the shock surface, different phenomena are observed in the upstream region. For $\theta _{\mathrm{Bn}}$ ranging between $70^{\circ}$ and $40^{\circ}$, the reflected ions can escape far upstream in the form of field-aligned ion beams (FAB, e.g., Fairfield 1969; Paschmann et al. 1980; Schwartz and Burgess 1984; Meziane et al. 2005) with energies of up to $\sim 10$ keV. FABs are also known as backstreaming ions or suprathermal ions and are responsible for the formation of the suprathermal ion foreshock (yellow region in Fig. 2). As explained below, FABs excite the 30-second waves. The region of the foreshock populated by ULF wave activity, including 30-second waves, is called the ULF wave foreshock (purple) and starts somewhat downstream of the suprathermal ion foreshock boundary. This is due to the fact that the ULF waves need some time to grow, during which they are convected earthward by the solar wind (Blanco-Cano et al. 2009; Turc et al. 2018). Sometimes, during periods of large solar wind plasma $\beta $ (ratio of the thermal to the magnetic pressures), there appear the so-called 3-second ULF waves superposed onto the 30-second waves (Le and Russell 1992b; Le et al. 1993; Blanco-Cano et al. 1999; Hobara et al. 2007; Wang et al. 2020b). Wang et al. (2020b) showed that these waves form due to the non-resonant ion beam instability.

Deep inside the foreshock, another suprathermal ion population is found, called the diffuse ions (blue region in Fig. 2, e.g., Lin et al. 1974; West and Buck 1976; Gosling et al. 1978; Paschmann et al. 1981; Bonifazi and Moreno 1981a,b; Winske and Leroy 1984). These particles exhibit almost isotropic velocity distributions and energies up to $\sim 100$ keV. As the foreshock ULF waves arrive to this region, they refract and steepen, which eventually leads to the formation of smaller-scale ($\lesssim 1$ Earth radius, $R_{\mathrm{E}} = 6371~\mathrm{km}$) transient phenomena.

### Generation and Properties of Foreshock ULF Waves

**Fig. 3:**
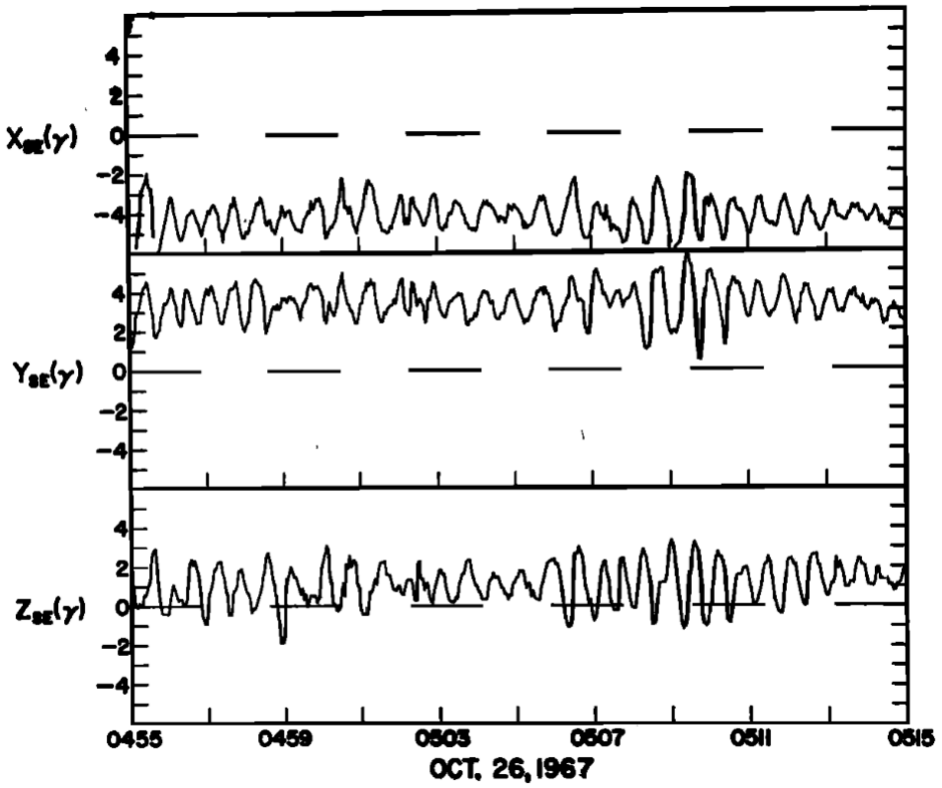
Large-amplitude quasi-sinusoidal waves with a period around 30 s on the three magnetic field components measured by the Explorer 34 spacecraft in the Earth’s foreshock. The waves were observed about $14~R_{\mathrm{E}}$ from the average bow shock position. Figure reproduced with permission from Fairfield (1969), copyright by the AGU

ULF wave activity in Earth’s foreshock is dominated by large-amplitude, quasi-sinusoidal waves with a period close to 30 seconds (e.g. Eastwood et al. 2005c; Wilson 2016). Two example intervals when these waves were observed by the Explorer 34 and the International Sun-Earth Explorer (ISEE) satellites are provided in Figs. 3 and 6, respectively. These waves were discovered in the 1960s, when measurements from the Vela 3A satellite revealed the presence of magnetic field fluctuations in the ULF range upstream of Earth’s bow shock (Greenstadt et al. 1968). Fairfield (1969) was the first to propose that FABs excite the electromagnetic ion beam-cyclotron instability, generating the observed low-frequency, right-hand mode waves with periods of 0.1 ion gyrofrequency ($\Omega _{ \mathrm{i}}$) and wavelengths of $\sim 1~R_{\mathrm{E}}$ (see also Barnes 1970; Gary 1978; Sentman et al. 1981; Eastwood et al. 2002). In the spacecraft data, $0.1 \Omega _{\mathrm{i}}$ roughly translates into periods of $\sim \,30$ s. These waves propagate in the same direction as the reflected ions, towards the upstream in the plasma rest frame, but are convected downstream by the solar wind. Consequently, in the spacecraft frame of reference, they appear left-handed.

A large body of theoretical work focuses on the different instabilities that can arise from the interaction of an ion beam with the solar wind plasma, in order to identify the source of foreshock waves. Sentman et al. (1981) calculated the electromagnetic dispersion relation for right- and left-hand circularly polarized ULF waves with propagation directions parallel and antiparallel to the IMF in the presence of two different kinds of suprathermal ion populations – FABs and diffuse ions (Paschmann et al. 1981). Sentman et al. (1981) found that the FABs destabilize the plasma most strongly at $\omega \sim 0.1\,\Omega _{\mathrm{i}}$ by way of a resonant ion beam instability for waves propagating upstream (in the plasma rest frame), and by way of a non-resonant firehose-like instability for waves propagating downstream. The diffuse ions destabilize the plasma most strongly at similar frequencies by way of resonant instability of both right- and left-hand polarized waves propagating away from the bow shock. The authors also calculated the growth times of these waves to be $\sim 60$ s for those associated with FABs and $\sim 150$ s for those associated with diffuse ions.

Gary et al. (1984) examined the linear theory of electromagnetic instabilities driven by relatively cold ion beams in a homogeneous Vlasov plasma. They found four distinct instabilities that produce waves propagating parallel to the magnetic field. For sufficiently energetic ion beams, two unstable right-handed modes appear, one resonant and one non-resonant. The first has a lower threshold and larger growth rate for a beam density $\lesssim 2\%$ of the incident solar wind density and the beam velocity $\lesssim 10 v_{\mathrm{A}}$ (Alfvén velocity). The resonant mode propagates in the direction of the reflected beam (towards upstream), while the non-resonant mode propagates in the direction opposite to the beam (towards downstream). The latter thus appear as right-handed waves also in the spacecraft frame of reference. On the other hand, for small beam velocities and sufficiently large $T_{\perp}/T_{\parallel}$ (temperatures perpendicular and parallel to the ambient magnetic field), a left-hand ion cyclotron instability develops, while hot beams of reflected ions drive a left-hand beam resonant mode. Because the FABs in Earth’s foreshock are tenuous, cool and propagate much faster than the Alfvén speed, and the plasma $\beta $ is relatively high, the right-hand resonant ion beam instability is the best candidate for the source of foreshock 30-second waves (Gary 1991, 1993).

Blanco-Cano and Schwartz (1997b) examined the electromagnetic dispersion relation derived from linear Vlasov theory in order to study the properties of ULF waves in the presence of a hot ion beam (e.g. diffuse ions). Right-hand and left-hand resonant instabilities under conditions consistent with the foreshock were considered. The authors used spacecraft observations by the AMPTE-UKS mission as inputs for their models. Comparison of the observed transport ratios of the waves observed during 10 intervals by the AMPTE-UKS with the theoretical values enabled Blanco-Cano and Schwartz (1997a) to identify regions in the foreshock populated by Alfvénic waves and others populated by magnetosonic fluctuations. Their results suggest that the observed Alfvén waves are generated locally by hot diffuse ions, while the right-handed waves may result from the superposition of waves generated by either cold FABs far upstream or locally by diffuse ions.

The launch of the Cluster mission (Escoubet et al. 2001) in 2000 brought about considerable advances in our understanding of foreshock waves. Its four-spacecraft constellation enabled characterising the wave properties with greater accuracy in employing multi-spacecraft analysis techniques such as timing analysis and the wave telescope/$k$-filtering method (e.g., Eastwood et al. 2002, 2003, 2005c; Archer et al. 2005; Hobara et al. 2007; Hnat et al. 2016; Turc et al. 2019). This allowed in particular to determine the wave properties in the plasma rest frame, and to compare the data with theoretical wave dispersion relations (e.g., Narita and Glassmeier 2005; Narita et al. 2006). More recently, measurements from the THEMIS (Angelopoulos 2008) and MMS (Burch et al. 2016) missions also contributed to our knowledge of 30-second waves. We summarise here our current understanding of their properties.

**Fig. 4:**
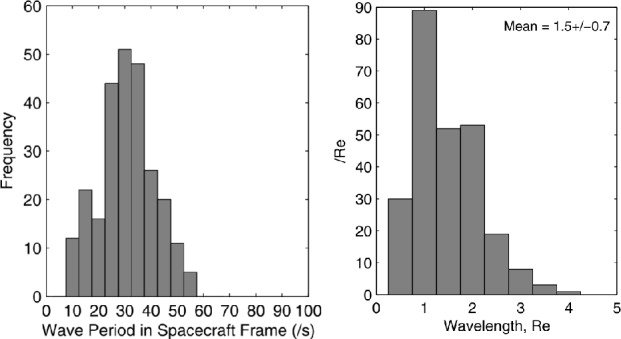
Histograms of the wave periods in the spacecraft frame and of the wavelengths of 30-second waves observed by the Cluster spacecraft during 255 2-min intervals in the foreshock in 2001. The wave period was determined from the average autocorrelation of the time series and the wavelength based on timing analysis between the four-spacecraft measurements. Figure adapted and reproduced with permission from Eastwood et al. (2005a), copyright by the AGU

Statistical studies have shown that the period of 30-second waves can vary between 10 and 80 s, depending mostly on the IMF strength (see example of a statistical survey in Fig. 4 (left) and Le and Russell 1996; Eastwood et al. 2005a; Hsieh and Shue 2013). Their observed wavelength typically ranges between 1 and 5 $R_{ \mathrm{E}}$, with a mean value around 1.5 $R_{\mathrm{E}}$ (see Fig. 4 (right) and Eastwood et al. 2005a; Archer et al. 2005). Their correlation length transverse to the wavevector tends to be much larger, 8–18 $R_{\mathrm{E}}$ (Archer et al. 2005), but decreases for large IMF values (Turc et al. 2019). Their propagation speed in the plasma rest frame is close to the Alfvén speed (Eastwood et al. 2002; Mazelle et al. 2003; Eastwood et al. 2005a), though other studies have shown that the dominant waves in the foreshock propagate at the fast-mode speed (Narita et al. 2006). They propagate at a small angle relative to the ambient magnetic field direction, of the order of $20^{\circ}$ on average (Le and Russell 1994; Eastwood et al. 2005b; Hsieh and Shue 2013).

The growth rate of foreshock 30-second waves was determined experimentally for one event observed by the Acceleration, Reconnection, Turbulence and Electrodynamics of Moon’s Interaction with the Sun (ARTEMIS) spacecraft near the Moon, yielding a growth rate $\gamma \sim 0.035 \Omega _{\mathrm{i}}$, consistent with theoretical predictions (Dorfman et al. 2017). Interestingly, although the 30-second waves are excited by the field-aligned ion beams, they are not observed simultaneously with them (e.g. Eastwood et al. 2005a; Turc et al. 2019). This is due to the fact that these waves need some time to grow during which they are convected towards downstream. As a result, these waves are observed together with intermediate ion populations (e.g. Argo et al. 1967; Eastwood et al. 2005a; Turc et al. 2019).

### Evolution of Foreshock ULF Waves

**Fig. 5:**
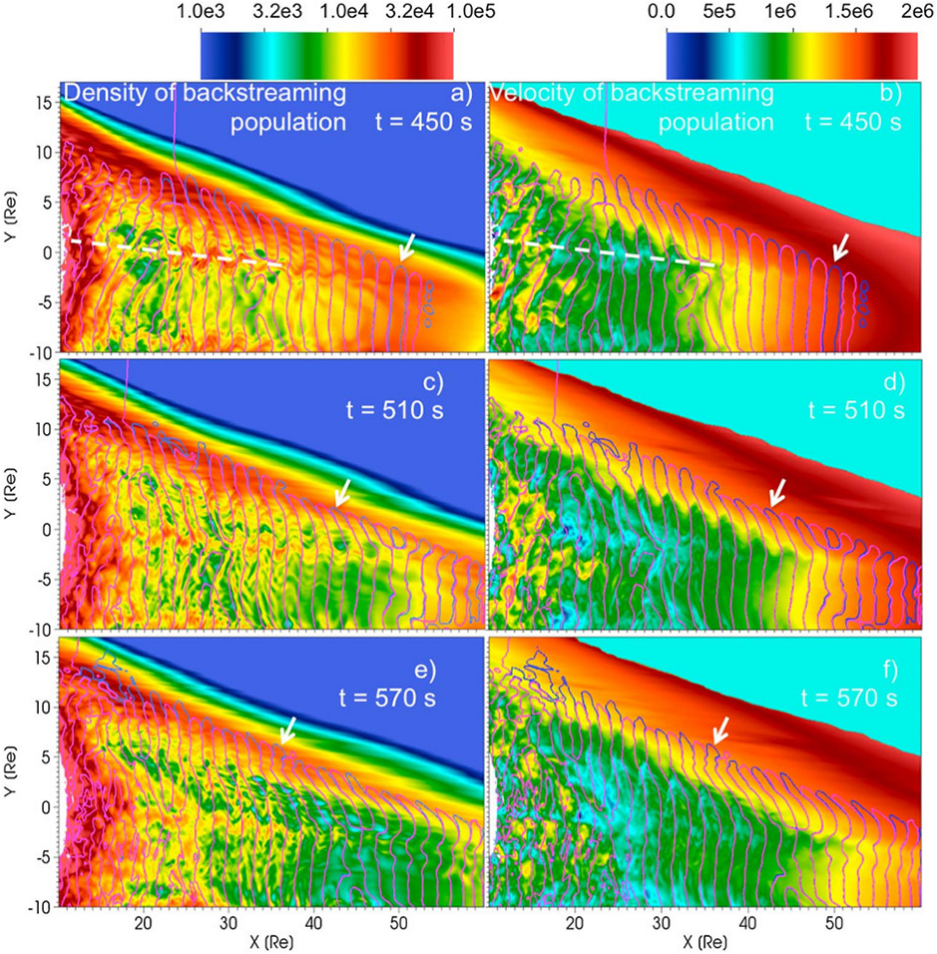
Refraction of the foreshock wave fronts as they are advected through regions of different suprathermal ion density and velocity, in a global 2D hybrid-Vlasov simulation with a quasi-radial IMF geometry. The white arrow tracks a single wave front at different times in the simulation. Figure reproduced with permission from Palmroth et al. (2015), copyright by the AGU

As the 30-second waves are carried by the solar wind into the region of the foreshock populated by diffuse ions (Winske and Leroy 1984; Paschmann et al. 1981; Bonifazi and Moreno 1981a,b), their propagation direction becomes more oblique (Le and Russell 1994; Eastwood et al. 2005b; Hsieh and Shue 2013), even though the growth rate of the ion-ion beam instability that causes the waves maximises in the parallel direction (Gary 1993). This conundrum has inspired many explanation attempts. For example, Winske (1985) suggested another mechanism for the wave growth based on the right-hand resonant instability near the shock. Killen et al. (1995) noted that also the beam-ring ion distributions could explain the wave obliquity. Hada et al. (1987) suggested refraction caused by a the spatial variation of the backstreaming ions as the cause for the oblique waves. Palmroth et al. (2015) used a 2D3V (2D in ordinary space and 3D in velocity space) hybrid-Vlasov (kinetic ions, fluid electrons) simulation run using Vlasiator to investigate the causes for the wave obliquity. They compared the simulation results to THEMIS data, and noticed that the foreshock waves are in good correspondence with observations. They suggested that the variability of the density and velocity of the backstreaming ions organise the large-scale structure of the foreshock, and that this spatial and temporal variability of the density and velocity causes the wave vector to refract from the parallel direction. This process is illustrated in Fig. 5.

**Fig. 6:**
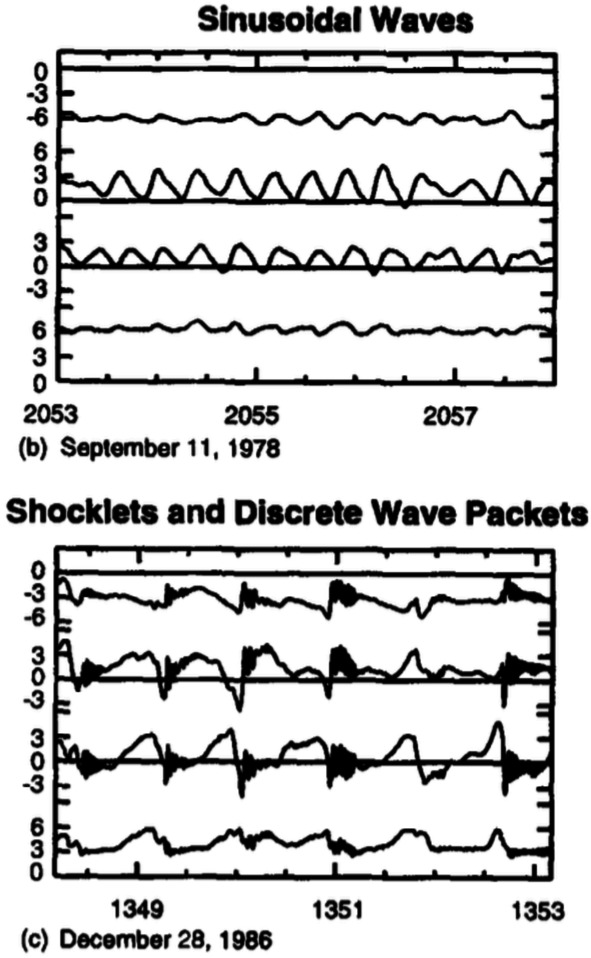
Examples of sinusoidal transverse ULF waves (top) and shocklets (bottom) in the Earth’s foreshock. Figure adapted from Greenstadt et al. (1995), reproduced with permission from COSPAR, copyright by Elsevier

When foreshock ULF waves approach the bow shock, wave steepening may cause them to become a type of structures called the shocklets (Hada et al. 1987; Omidi and Winske 1990, and illustrated in the bottom part of Fig. 6). These are highly compressive, obliquely propagating magnetosonic fluctuations with a highly steepened upstream edge commonly associated with a discrete wave packet of oblique whistler mode waves (Russell et al. 1971; Russell and Hoppe 1983; Hoppe et al. 1981). Although they are predominantly right-handed in the plasma rest frame, shocklets can sometimes exhibit a left-hand polarisation (Hoppe and Russell 1983). Their scale sizes are of the order of a few $R_{\mathrm{E}}$, comparable with those of 30-second waves, thus supporting a possible connection between these waves and shocklets (Hoppe et al. 1981; Lucek et al. 2002).

**Fig. 7:**
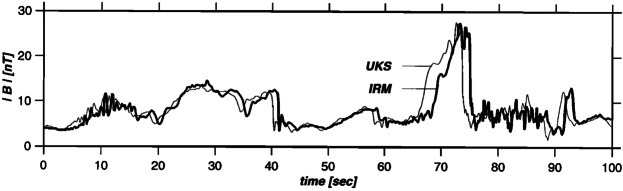
Observations of a single short large-amplitude magnetic structure (SLAMS) by two space missions. Figure reproduced with permission from de Wit et al. (1999), copyright by the AGU

If their amplitude continues growing, foreshock ULF waves may evolve into short-large amplitude magnetic structures (SLAMS, Schwartz 1991; Schwartz et al. 1992; Giacalone et al. 1993; Dubouloz and Scholer 1995a,b; Scholer et al. 2003; Tsubouchi and Lembège 2004; Lucek et al. 2004, 2008, see Fig. 7). SLAMS are observed as relatively featureless pulses of magnetic field and plasma density with durations of $\sim 10$ s in the spacecraft data. They are typically defined as structures where the peak magnetic field magnitude reaches at least twice the value in the ambient plasma (e.g. Schwartz et al. 1992; Lucek et al. 2004). Their polarisation can be both left-handed and right-handed, with sometimes both polarisations being encountered within a single structure (Schwartz et al. 1992; Lucek et al. 2004). Close to the shock, the correlation lengths of SLAMS are much smaller than the wavelength of the 30-second waves (e.g. Lucek et al. 2008). In the directions parallel and perpendicular to the shock normal, the SLAMS extensions are typically only between about 15 and a few tens of upstream ion inertial lengths ($d_{ \mathrm{i}} \sim 100-150$ km upstream of Earth), respectively (Dubouloz and Scholer 1995b,a; Lucek et al. 2002).

The processes giving rise to SLAMS, and whether shocklets and SLAMS are successive evolutionary stages of ULF waves or completely distinct phenomena, are still to date the topic of intensive scrutiny. In a recent study, Chen et al. (2021b) have shown that 3-second waves can steepen into SLAMS, consistent with the smaller scale sizes of these structures compared to the wavelength of 30-second waves.

Refracted ULF waves are compressive and propagate obliquely with respect to the ambient IMF. Their interaction with locally-generated transverse ULF waves can result in the formation of transient foreshock structures known as foreshock cavitons (Omidi and Sibeck 2007; Blanco-Cano et al. 2009; Kajdič et al. 2011, 2013). These are observed in spacecraft data as simultaneous depletions of magnetic field magnitude and plasma density that are surrounded by rims where these two quantities are enhanced. As the cavitons approach the bow shock, they interact with it and evolve into spontaneous hot flow anomalies (SHFA, Zhang et al. 2013; Omidi et al. 2013, 2016; Tarvus et al. 2021). In the spacecraft data and hybrid simulations, SHFAs exhibit cores of even more depleted magnetic field and plasma density with highly enhanced ion temperature.

Foreshock cavitons and SHFAs are two types of transient mesoscale structures commonly encountered in the foreshock. Transient mesoscale structures, most frequently referred to as *foreshock transients* are characterised by modified plasma and magnetic field properties compared with the ambient foreshock, and have sizes ranging from the local ion gyroradius up to $\sim 10~R_{\mathrm{E}}$ and durations from several seconds up to several minutes in spacecraft data (Zhang and Zong 2020). It has been shown that the largest of these transient structures, namely hot flow anomalies and foreshock bubbles, can cause displacements of the magnetopause and are a source of magnetospheric Pc3 and Pc5 waves (with periods in the $10-45$ s and $150-600$ s ranges, respectively, e.g. Eastwood et al. 2011; Hartinger et al. 2013b; Wang et al. 2017, 2020a, 2021).

## Interaction of Foreshock Waves with the Bow Shock

### Shock Reformation and Rippling

ULF waves, shocklets, SLAMS, foreshock cavitons and SHFAs participate in what is known as the nonstationarity or reformation of the quasi-parallel shock (Burgess 1989; Thomas et al. 1990). These terms refer to quasi-periodical changes of the shock structure. Scholer (1993) showed that the dominant process of quasi-parallel shock reformation involves SLAMS that are convected towards the shock and strongly steepen $\sim 20\,d_{\mathrm{i}}$ upstream of it, eventually becoming a new shock front whose magnitude exceeds that of the old front (see also Onsager et al. 1991; Scholer and Burgess 1992; Hao et al. 2017; Lefebvre et al. 2009; Johlander et al. 2022). Conclusive observational evidence of quasi-parallel shock reformation due to SLAMS was recently reported by Johlander et al. (2022), using data from the MMS mission. They showed that the transition from upstream to downstream was first observed by the spacecraft located furthest in the sunward direction and last by the satellite closest to Earth, implying that the shock crossing was due to a SLAMS merging with and replacing the shock front.

Shock reformation can also occur for oblique shock configurations ($\theta _{\mathrm{Bn}}\sim 45^{\circ}$), near the edge of the ULF wave foreshock, which typically extends to $\theta _{\mathrm{Bn}} \sim 50^{\circ}$ (e.g. Le and Russell 1992a; Andrés et al. 2015). Since the foreshock waves there are not as steepened as those upstream of the quasi-parallel shocks and no SLAMS form there, reformation processes at marginally quasi-perpendicular shocks are somewhat different, involving foreshock ULF waves. The first direct observational evidence for the reformation of the oblique bow shock by the foreshock ULF waves was presented by Liu et al. (2021), taking advantage of the MMS data when these spacecraft formed a string-of-pearls configuration and crossed the bow shock near $\theta _{\mathrm{Bn}}=50^{\circ}$. Liu et al. (2021) found that after the interaction with one period of the ULF wave, a new bow shock formed that exhibited a sharp transition region upstream of the old bow shock remnant.

**Fig. 8:**
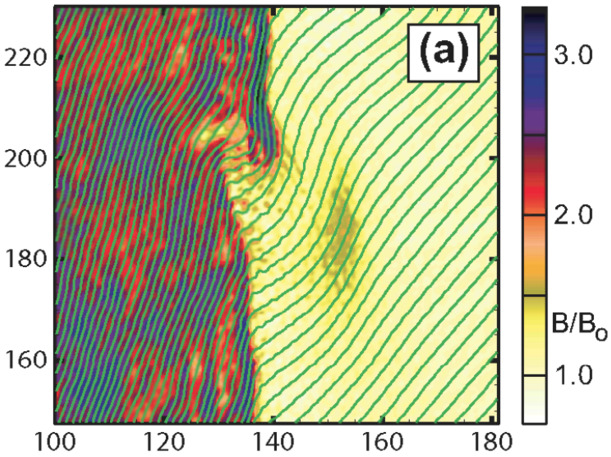
Bending of magnetic field lines at the shock transition in local hybrid simulations by Krauss-Varban et al. (2008). Figure reproduced with permission from Krauss-Varban et al. (2008), copyright by AIP Publishing

Since the process of shock reformation is not in phase along the whole quasi-parallel section of the shock surface, shocklets and SLAMS are responsible for another phenomenon, called the shock surface rippling. This corresponds to local deformations of the shock front. Large-amplitude ULF waves also contribute to this phenomenon by bending the magnetic field lines just upstream of the shock transition (see Burgess 1995; Krauss-Varban et al. 2008, and Fig. 8). Shock rippling means that the surface of the shock is not smooth. Instead, the location of the shock transition, the local normal direction and the $\theta _{\mathrm{Bn}}$ angle vary with the location on the shock and in time.

Shock rippling was first identified in hybrid numerical simulations (e.g. Burgess 1989; Schwartz and Burgess 1991; Krauss-Varban and Omidi 1991; Scholer and Terasawa 1990; Scholer 1993; Scholer et al. 1993; Hao et al. 2016, 2017). Direct observations of the rippled quasi-parallel shock were reported by Gingell et al. (2017) in the data of the MMS mission. They showed that the observed shock ripples were a transient phenomenon, occurring over time scales shorter that the ion gyroperiod, and closely linked with shock reformation.

As we shall see, shock rippling may have an important role in the wave-shock wave interaction, since it means that different portions of the upstream waveforms encounter sections of the shock with varying strength and geometry.

### Transmission of Foreshock Waves Through the Shock

The similarities between foreshock 30-second waves and magnetospheric Pc3 waves have led many workers to invoke a direct transmission of foreshock waves across the bow shock (e.g. Russell et al. 1983; Clausen et al. 2009; Francia et al. 2012; Takahashi et al. 2016), although there exist investigations that may not share this point of view (Engebretson et al. 1991a). As we will detail below, vastly different results are obtained for the interaction of fast-mode waves with a shock depending on whether one considers this interaction in a magnetohydrodynamic (MHD) or in an ion-kinetic framework. This controversy remained unresolved because no clear evidence of the fast-mode waves predicted by the direct transmission scenario had been found inside the magnetosheath. At the same time, the change in the wave mode through the bow shock in the competing theory precludes the wave transmission into the magnetosphere, and thus was inconsistent with observations of magnetospheric Pc3 waves.

The interaction of various types of plasma waves with a shock and their possible transmission into the downstream have been extensively studied using theory and simulations. Early MHD theoretical considerations, such as those by, for example, McKenzie and Westphal (1969), Asséo and Berthomieu (1970), Hassam (1978) and Whang et al. (1987), used linearized Rankine-Hugoniot equations to study the interaction of Alfvén and magnetosonic waves with a shock wave. These investigations showed that the interaction between a shock wave and a single upstream wave results in multiple downstream MHD waves, some propagating towards the upstream and some towards the downstream (in the plasma rest frame). In general, six waves can be excited downstream of an MHD shock: a forward and a reverse Alfvén wave, a forward and a reverse slow-mode wave, an entropy wave and a forward fast-mode wave (e.g. McKenzie and Westphal 1970; Whang et al. 1987). Note that there is no reverse fast-mode wave because that wave is the shock itself. Which of these waves are effectively generated depends on the geometry of the interaction between the upstream wave and the shock, specifically the angle of incidence (between the shock normal and the wave vector). Above a critical incidence angle ($\sim 60^{\circ}$), some of the wave modes cannot propagate into the downstream, in general the fast-mode wave (Whang et al. 1987).

The upstream wave also tends to be strongly amplified as it transmits through the shock. This amplification again depends mostly on the angle of incidence (Westphal and McKenzie 1969; McKenzie 1970; McKenzie and Westphal 1970; Asséo and Berthomieu 1970). In the special case of an incident Alfvén wave with its wave vector lying in the shock plane and for conditions representative of Earth’s bow shock, McKenzie and Westphal (1969) found a two- to threefold increase in the wave amplitude in one of the downstream Alfvén waves and a similar amplitude as in the upstream in the other Alfvén wave. McKenzie and Westphal (1970) further showed that pressure variations associated with fast-mode waves experience the largest increase through the shock, while magnetic field variations in fast-mode and Alfvén waves are enhanced only by a factor of 4.

These theoretical derivations of the properties of the downstream waves are based on the assumption that wave refraction through the shock obeys Snell’s law. This implies that there is continuity of the wave frequency and of the component of the wave vector tangential to the shock (e.g. Westphal and McKenzie 1969). As a result, the orientation of the wave vector of the transmitted wave is the same as that of the incident wave. The downstream counterparts of incoming waves with an upstream-oriented wavevector, such as foreshock 30-second waves, would thus retain the same wavevector orientation. We also note that the wave amplitudes are considered to be small, so that the continuity equations can be solved in the linear approximation, but this assumption may not hold as foreshock waves tend to grow in amplitude and steepen as they approach the shock (e.g. Le and Russell 1992b).

The numerical MHD simulations performed by Park et al. (2002) have shown that an array of MHD waves is similarly excited in the downstream when density or pressure pulses, instead of MHD waves, impinge on the bow shock. In particular, the interaction of a fast/slow mode-like pulse with the shock resulted in stronger fast mode waves in the downstream. This could be relevant for the interaction of foreshock waves which have developed a significant compressional component, as is typically the case in the vicinity of the shock (Le and Russell 1992b).

**Fig. 9:**
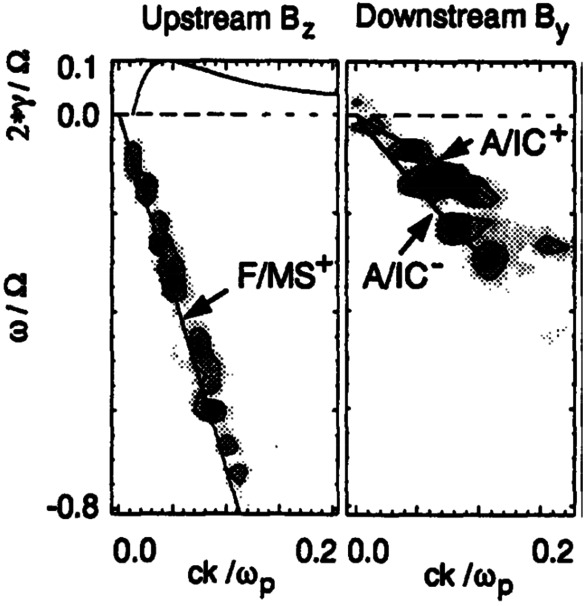
Power spectra of the waves upstream and downstream of the shock in a 1-D local hybrid-PiC simulations performed by Krauss-Varban (1995). From Krauss-Varban (1995), reproduced with permission from COSPAR, copyright by Elsevier

Because foreshock waves are driven by kinetic processes, kinetic theory and simulations are better suited to study their generation and their interaction with the quasi-parallel shock. Local hybrid-Particle-in-Cell (PIC) simulations performed by Krauss-Varban and Omidi (1991, 1993), Krauss-Varban et al. (1994), Krauss-Varban (1995) uncovered a new picture for foreshock wave transmission through the shock, contrasting with previous works based on MHD. In a hybrid-kinetic setup, they found that fast-mode waves from the foreshock cannot retain their fast-mode nature when they cross the bow shock, but are mode-converted into Alfvén waves and/or slow modes at the same frequency in the downstream. This is due to the fact that the fast mode is no longer accessible in the downstream at the foreshock wave frequency. Examples of wave power spectra obtained in the numerical simulations performed by Krauss-Varban (1995) are shown in Fig. 9, where the left panel shows compressional waves in the foreshock and the right panel shows Alfvén waves due to mode conversion in the downstream. The majority of the downstream Alfvén wave power is found on the branch that propagates towards the upstream in the plasma rest frame (see Fig. 9), but is convected into the downstream by the plasma bulk flow. Subsequent observational studies appeared to confirm the findings by Krauss-Varban and co-workers that foreshock 30-second waves cannot retain their fast-mode nature downstream of the shock, as no clear evidence of fast-mode waves could be found in the Earth’s magnetosheath. This will be discussed further in Sect. 4, where we review these observational works.

**Fig. 10:**
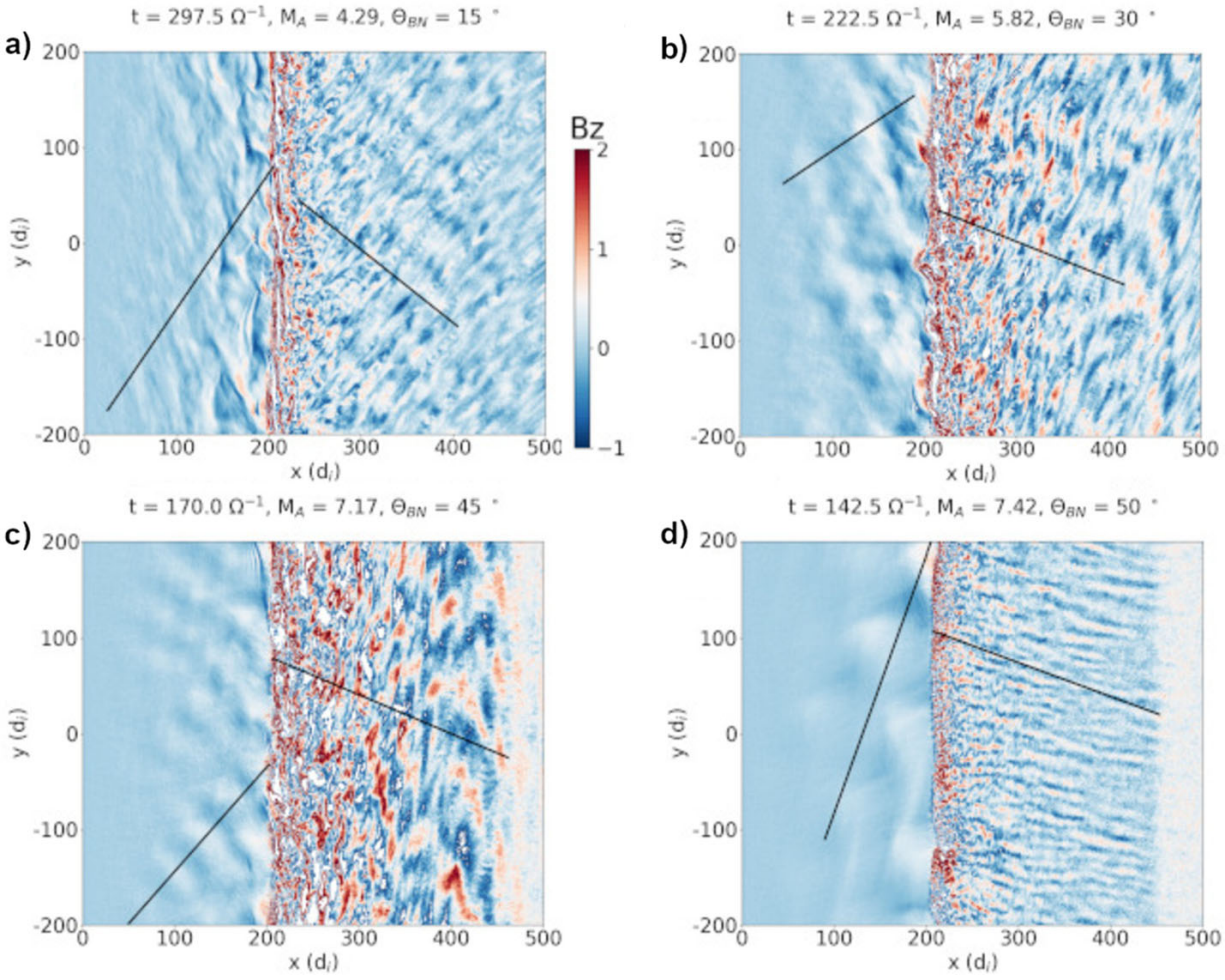
Simulation results for 4 out of 11 local hybrid runs from Kajdič et al. (2021) at times when the shock was located in the middle of the simulation domain. Colours represent the magnetic field magnitude. $\theta _{\mathrm{Bn}}$ increases from left to right while the shock’s Alfvénic Mach number ($\mathrm{M}_{\mathrm{A}}$) increases from top to bottom. The shocks on panels a, c and d are classified by Kajdič et al. (2021) as weakly rippled, while the shock on panel b is strongy rippled. Figure adapted and reproduced with permission from Kajdič et al. (2021), copyright by the AGU

Kajdič et al. (2021) performed a parametric study using numerical simulations of foreshock and magnetosheath waves in order to study the possible wave transmission across the shock. They conducted eleven local hybrid-PIC simulations of collisionless shocks (Fig. 10) with Alfvénic Mach numbers ranging between 4.29 and 7.42 and $\theta _{\mathrm{Bn}}$ values between $=15{^{\circ}}$ and $50^{\circ}$. The properties of the waves, such as their wavelength and their amplitude, were compared in the foreshock and the magnetosheath. They noted that the properties of upstream waves is very different from those of the downstream waves. The authors interpreted this to be at least partially due to shock rippling: as the upstream ULF waves arrive to the shock, different sections of a single waveform encounter a shock with different strength, shock normal orientation and $\theta _{\mathrm{Bn}}$. Thus, the upstream waveforms are heavily modified or even fragmented and destroyed by the shock.

The spectra of upstream and downstream waves were also compared. It was shown that the ULF wave signatures that were present in the spectra of the compressional component of foreshock waves also appeared in the spectra of the magnetosheath fluctuations. These features were better conserved in the case of what the authors classified as weakly rippled shocks (Fig. 10a, c and d), while the spectra of the waves downstream of strongly rippled shocks (Fig. 10b) tended to be more featureless and turbulence-like. Downstream transverse spectra exhibited mostly featureless, turbulence-like spectra. Kajdič et al. (2021) concluded that although the properties of upstream and downstream ULF waves are very different, some spectral features in the ULF wavelength range are conserved. This information could in principle be convected all the way to the magnetopause and eventually cause the Pc3 fluctuations in the magnetosphere.

**Fig. 11:**
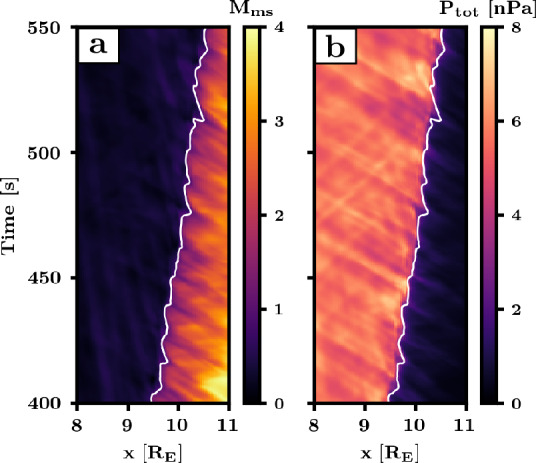
Total pressure variations in the magnetosheath caused by the foreshock waves. Time-position maps of the magnetosonic Mach number (a) and the total pressure (b) along the Sun-Earth line. The total pressure is calculated as the sum of the thermal pressure and the magnetic pressure. The white contour marks where $\mathrm{M}_{\mathrm{ms}} = 1$. From Turc et al. (2023)

In a recent work, Turc et al. (2023) returned to the issue of 30-second foreshock wave interaction with the bow shock and proposed a new scenario for their transmission into the downstream. This study examined the wave properties in a 2D global ion-kinetic simulation of the near-Earth environment performed with the Vlasiator model (Palmroth et al. 2018). Fast-mode fluctuations at similar periods were found both in the foreshock and the magnetosheath, but the orientation of the wavevectors changed from sunward in the foreshock to earthward inside the magnetosheath. Such a change is inconsistent with a direct transmission through the shock, as described with MHD theory. A closer look at the interaction of the foreshock waves with the shock revealed that the compressional component of the waves modulates the magnetosonic Mach number just upstream of the shock, and consequently its compression ratio (see Fig. 11). This results in variations of the total pressure in the downstream, which were identified as the source of the magnetosheath fast-mode waves. This mechanism, in which changes in upstream magnetosonic Mach number lead to fast-mode waves being launched in the downstream, was first proposed by Wu et al. (1993) for the interaction of discontinuities with a shock. Here it was shown for the first time to also apply to compressional waves impinging on a shock.

The results presented in Turc et al. (2023) also propose a solution to the conundrum posed by the numerical simulations of Krauss-Varban and co-authors (Krauss-Varban and Omidi 1991; Krauss-Varban et al. 1994; Krauss-Varban 1995), in which the mode conversion to Alfvén waves precluded any entry of the downstream waves into the magnetosphere. In addition to the fast-mode waves discussed above, the Vlasiator simulation also shows signatures of Alfvén waves in the downstream, likely corresponding to mode-converted foreshock waves (Krauss-Varban and Omidi 1991; Krauss-Varban et al. 1994; Krauss-Varban 1995). It was further noted that the foreshock waves participate in shock reformation, creating structures propagating with the bulk flow in the magnetosheath, as reported by Liu et al. (2021). The global simulation thus reveals that the interaction of foreshock waves with the shock is multi-faceted, with multiple processes occurring simultaneously: shock reformation, mode conversion and modulation of the shock compression ratio leading to the generation of fast-mode waves in the downstream. Only the latter does however play a role in the propagation of foreshock 30-second waves towards the magnetosphere.

Interestingly, the coexistence of Alfvén waves and downstream-oriented fast-mode waves was reported previously in another numerical work which focused on the propagation of upstream pressure pulses through a shock, using a hybrid-PIC model (Thomas et al. 1995). For a quasi-parallel shock configuration, these pressure pulses were self-consistently generated in the simulation, with properties resembling those of 3-second foreshock waves. The pressure pulses were propagating towards the upstream in the plasma rest frame, but were carried towards the shock by the faster plasma flow, as the 30-second waves. Thomas et al. (1995) found that the upstream pressure pulses continue to propagate into the downstream over large distances, at the fast-mode speed in the plasma rest frame. They described the generation of the downstream fast-mode waves as an “impulsive” process at the shock, due to the impact of the incoming pressure pulses, similar to the mechanism proposed by Wu et al. (1993).

The scenario for foreshock wave transmission proposed by Turc et al. (2023) may provide the missing link between foreshock 30-second and magnetospheric Pc3 waves. The magnetosheath counterparts of 30-second waves are not directly-transmitted foreshock waves but new fluctuations created by the modulation of the bow shock properties by the foreshock waves. The newly-created waves exhibit periods similar to those of the foreshock waves, consistent with observations showing that the wave period is preserved from the foreshock to the magnetosphere (e.g. Clausen et al. 2009; Regi et al. 2014), and in agreement with the recent numerical work by Kajdič et al. (2021) showing enhanced compressional wave power in the downstream at the foreshock wave frequency. The findings of Turc et al. (2023) also reconcile the seemingly incompatible competing theories of wave transmission and mode conversion in showing that both processes occur simultaneously at the bow shock.

## Propagation of Foreshock Waves Through the Magnetosheath

The Earth’s magnetosheath, bounded by the bow shock on its upstream side and the magnetopause in the downstream, acts as a natural interface between the solar wind and the magnetosphere (e.g. Lucek et al. 2005). It houses solar wind plasma processed by the shock — and thus hotter, denser and slower than its upstream counterpart — which flows around the magnetic obstacle formed by Earth’s magnetopause. The magnetosheath properties show significant spatial variations and strongly depend on the nature of the shock transition, quasi-parallel or quasi-perpendicular, encountered upon crossing the bow shock. As the quasi-parallel shock typically lies on the dawn flank because of the Parker spiral orientation of the IMF at Earth, pronounced dawn-dusk asymmetries are observed in the magnetosheath properties (e.g. Walsh et al. 2014; Dimmock et al. 2017). Downstream of the quasi-perpendicular shock, the dusk magnetosheath is characterised by larger velocities and magnetic field strengths (Longmore et al. 2005; Dimmock et al. 2017; Turc et al. 2020), as well as a larger temperature anisotropies, defined as the ratio of the perpendicular and parallel temperatures (Dimmock et al. 2015b). In contrast, the quasi-parallel magnetosheath contains a hotter and denser plasma (Walsh et al. 2012; Dimmock et al. 2015a; Turc et al. 2020), with larger-amplitude magnetic field and velocity fluctuations (Dimmock et al. 2014, 2016). These dawn-dusk asymmetries are reflected in the wave modes growing in the magnetosheath. Mirror mode waves are predominantly found in the quasi-perpendicular magnetosheath where the conditions of significant temperature anisotropy combined with a high plasma $\beta $ favour the mirror instability (Czaykowska et al. 2001; Soucek et al. 2015; Dimmock et al. 2015b). Under conditions of lower plasma $\beta $, the ion temperature anisotropy results in the growth of Alfvén-ion cyclotron (AIC) waves. These are typically observed in the plasma depletion layer close to the magnetopause, but also in the quasi-parallel magnetosheath where mirror mode waves are practically absent (Anderson and Fuselier 1993; Soucek et al. 2015; Anderson et al. 1994).

**Fig. 12:**
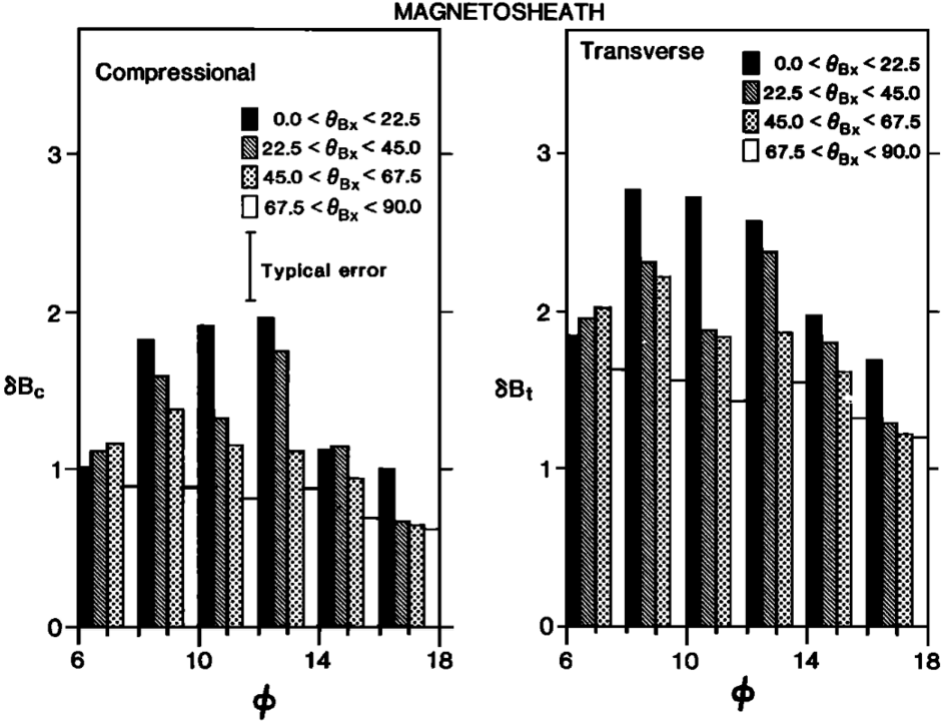
Average magnetic field parallel and transverse standard deviations as a function of magnetic local time (denoted here as $\Phi $), measured in the magnetosheath by the ISEE 2 spacecraft between October 1977 and October 1979. The data are separated into four cone angle bins, showing an enhanced level of fluctuations for low cone angle values. Figure reproduced with permission from Luhmann et al. (1986), copyright by the AGU

The quasi-parallel magnetosheath is characterised by an increased level of magnetic field fluctuations in the ULF range (Luhmann et al. 1986; Czaykowska et al. 2001; Shevyrev et al. 2006, 2007; Dimmock et al. 2014), as illustrated in Fig. 12. However, whether these are transmitted from the foreshock, are generated at the quasi-parallel bow shock or grow locally in the magnetosheath remains unresolved. It is likely that a combination of all three occurs, but their relative importance is unclear. Furthermore, to what extent the properties of the foreshock waves are preserved, if at all, upon crossing the shock, is unknown.

**Fig. 13:**
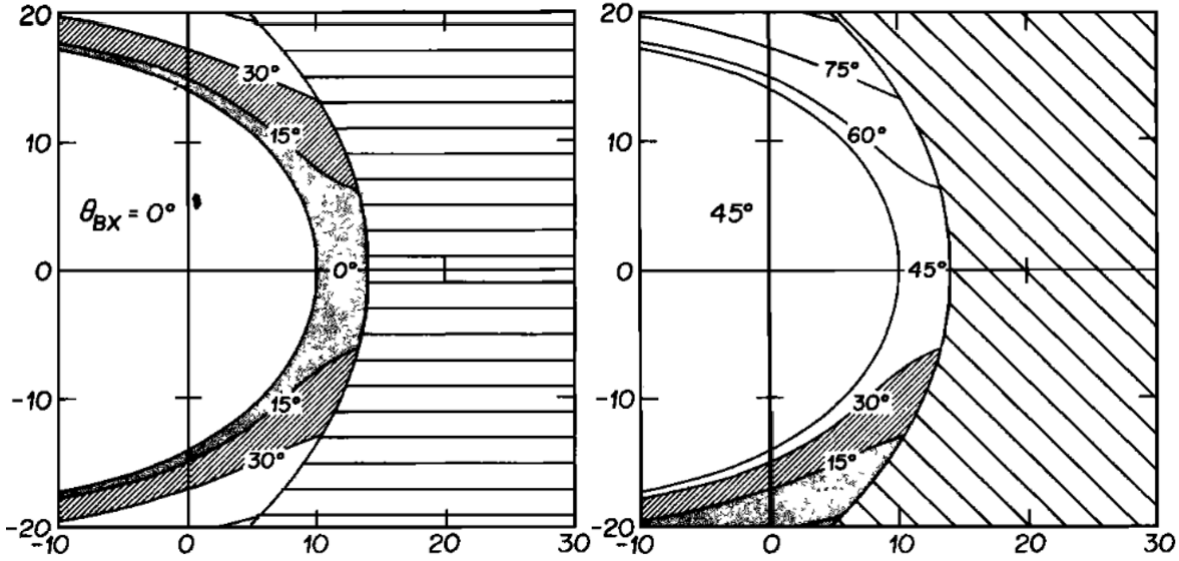
Flowlines of the magnetosheath plasma as a function of the IMF cone angle, for radial IMF ($\theta _{\mathrm{Bx}} = 0$, left) and Parker spiral IMF orientation ($\theta _{\mathrm{Bx}} = 45^{\circ}$, right), in the plane containing the IMF and solar wind velocity and passing through the subsolar point. The labels of the flowlines correspond to the $\theta _{\mathrm{Bn}}$ value encountered upon crossing the bow shock. Figure adapted and reproduced with permission from Russell et al. (1983), copyright by the AGU

As we will detail in Sects. 5 and 6, Pc3 waves are thought to enter the dayside magnetosphere as compressional fluctuations. The simplest scenario to explain wave transmission from the foreshock into the magnetosphere is therefore that the 30-second waves retain their fast-mode nature across the shock and through the magnetosheath, all the way to the magnetopause (Greenstadt et al. 1980; Russell et al. 1983; Odera 1986). Russell et al. (1983) proposed a model of the wave propagation following the magnetosheath plasma streamlines, as illustrated in Fig. 13. This model explains the different occurrence rates of Pc3 pulsations measured on the ground as a function of the IMF cone angle $\theta _{\mathrm{Bx}}$, measured between the IMF vector and the Sun-Earth line. For the sake of simplicity, Russell et al. (1983) assume that the wave phase speed is zero in the plasma rest frame, and thus that the waves are convected by the magnetosheath flow. The authors however infer that the transmitted waves likely have a non-zero phase speed inside the magnetosheath. It is worth noting that the Russell et al. (1983) model is essentially geometrical and cannot account for possible changes in the wave properties across the bow shock. According to this model, narrowband fast-mode waves with a period around 30 s should be observed in the magnetosheath downstream of the foreshock. However, no conclusive observations of such waves have come to corroborate this model.

Engebretson et al. (1991b) investigated simultaneous observations in the foreshock, magnetosheath and magnetosphere performed by the ISEE 1 and 2 and AMPTE IRM and CCE satellites. Increased turbulence in the magnetosheath was observed in conjunction with low IMF cone angle and Pc3–4 wave activity in the dayside magnetosphere, but the data did not show the expected narrowband signal at the foreshock wave frequency. Instead, it contained broadband power enhancements in magnetic field fluctuations near this frequency in the magnetosheath, which may play a role in the wave transmission. However, it is unclear how this broadband signal could be transformed into narrowband fluctuations in the dayside magnetosphere. Engebretson et al. (1991b) concluded that the spacecraft likely did not sample the volume of plasma which carried the wave information through the magnetosheath.

**Fig. 14:**
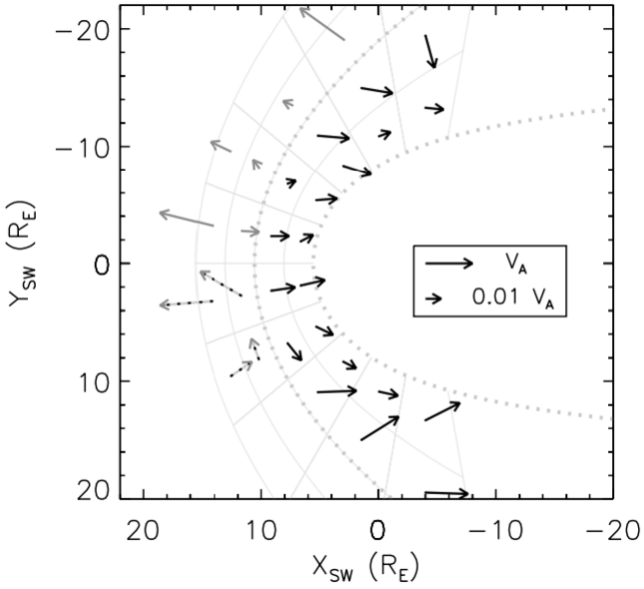
Direction of propagation of the dominant wave modes upstream and downstream of the bow shock, based on a statistical survey of Cluster data. From Narita and Glassmeier (2006)

Detailed case studies and extensive statistical surveys of ULF waves in the magnetosheath were later carried out using Cluster four-point measurements (e.g. Narita and Glassmeier 2005; Schäfer et al. 2005; Narita et al. 2006; Narita and Glassmeier 2006; Génot et al. 2011; Soucek et al. 2015). In the high-latitude magnetosheath, Schäfer et al. (2005) identified a mixture of Alfvén waves and mirror modes, the latter including both standing and propagating structures. Narita and colleagues investigated wave transmission from the foreshock into the magnetoseath in comparing the characteristics of the waves upstream and downstream of the bow shock (Narita and Glassmeier 2005; Narita et al. 2006). Using the wave telescope/$k$-filtering technique, Narita et al. (2006) retrieved the wave properties in the plasma rest frame for about 200 magnetosheath intervals in 2002, when the spacecraft separation was around 100 km, thus resolving wavelengths down to 200 km. For each interval, they focused on the dominant wave mode, based on the magnetic field power spectrum. They found that the wave polarisation changes from mostly circular in the upstream to mostly linear in the downstream, and that the wavevectors are roughly perpendicular to the ambient field in the magnetosheath, contrasting with the mostly parallel propagation in the foreshock. Both the transport ratios and the wave phase velocities are indicative of slow modes downstream of the shock, again departing from the upstream wave properties corresponding to fast modes. These suggest that magnetosheath wave activity is dominated by mirror modes, in agreement with the initial findings of Narita and Glassmeier (2005) for a single magnetosheath interval. Magnetosheath fluctuations exhibit an anti-sunward propagation (see Fig. 14 Narita et al. 2006; Narita and Glassmeier 2006), and thus do not match the upstream-propagating waves identified in the numerical works by Krauss-Varban and co-authors as mode-converted foreshock waves downstream of the shock (Krauss-Varban and Omidi 1991; Krauss-Varban 1995), as detailed in Sect. 3. Somewhat counterintuitively, similar wave properties were found downstream of both quasi-perpendicular and quasi-parallel shocks, suggesting that the shock and the foreshock would not influence magnetosheath wave activity. Based on these results, Narita and co-workers concluded that foreshock waves are not transmitted into the quasi-parallel magnetosheath.

Gutynska et al. (2012) analysed data collected during a conjunction between the GEOTAIL, Cluster and THEMIS spacecraft, providing simultaneous observations in the foreshock, the quasi-parallel and the quasi-perpendicular magnetosheath, respectively. They found no significant correlation of the fluctuations of the magnetic field magnitude upstream and downstream of the shock for this interval, suggesting once again that foreshock fluctuations are destroyed upon crossing the shock and new fluctuations are generated. It is worth noting that while GEOTAIL was located within a few $R_{\mathrm{E}}$ from the ecliptic plane, Cluster was at much higher latitudes, near $z = -10~R_{\mathrm{E}}$, during this event. As in the study by Engebretson et al. (1991b), the spacecraft were likely observing different plasma parcels, which may explain the absence of correlated fluctuations.

While the magnetosheath counterparts of foreshock waves remained elusive in spacecraft measurements, numerical simulations on the other hand suggest that foreshock 30-second waves can survive in some form inside the magnetosheath. As mentioned in Sect. 3, local shock simulations indicate that foreshock waves are mode-converted into Alfvén waves as they cross the shock (Krauss-Varban and Omidi 1991; Krauss-Varban 1995). This mode conversion, or in other words the loss of their compressional nature downstream of the shock, is however incompatible with the waves entering the magnetosphere as compressional waves (see for example the discussion in Regi et al. 2013). In global 2D hybrid simulations, Blanco-Cano et al. (2006) identify a mixture of waves downstream of the quasi-parallel shock, including mirror modes and magnetosonic waves. The latter may play a role in the transmission of foreshock waves to the magnetopause. Whether these waves are locally generated inside the magnetosheath or originate from the bow shock of the foreshock is however unclear.

An alternative wave transmission scenario was proposed by Engebretson et al. (1991b), in which localised variations of the magnetosheath dynamic pressure, due to the variability of the magnetosheath properties downstream of the quasi-parallel shock, would cause magnetopause motions. These would in turn trigger magnetospheric Pc3 waves. This scenario is closely linked with the high-latitude entry mechanism for Pc3 waves detailed in Engebretson et al. (1991a), through modulated electron precipitation or field-aligned currents in the cusp boundary layer (see Sect. 5.3), different from the compressional wave entry at equatorial latitudes.

Despite the lack of observational evidence supporting direct wave transmission through the magnetosheath, this process is still widely invoked in recent studies, assuming that foreshock waves traverse this region without significant changes to their properties (e.g. Clausen et al. 2009; Francia et al. 2012; Takahashi et al. 2016). We note that the magnetosheath crossing is not central to these works, which focus on foreshock and magnetospheric observations. Overall, the topic of wave transmission through the magnetosheath has been left largely unexplored after the detailed observational, numerical and theoretical investigations carried out from the 1970s to the 1990s (e.g. Greenstadt et al. 1980; Russell et al. 1983; Engebretson et al. 1991b; Krauss-Varban and Omidi 1991; Krauss-Varban 1995).

In the recent years, the continuing operations of the Cluster, GEOTAIL, THEMIS and MMS missions have provided new opportunities for simultaneous observations in the foreshock, quasi-parallel magnetosheath and dayside magnetosphere. At the same time, large-scale ion-kinetic simulations (e.g. Shi et al. 2017, 2021; Palmroth et al. 2018; Sibeck et al. 2021) are bringing a global view on the wave transmission which could facilitate the identification of relevant observations.

**Fig. 15:**
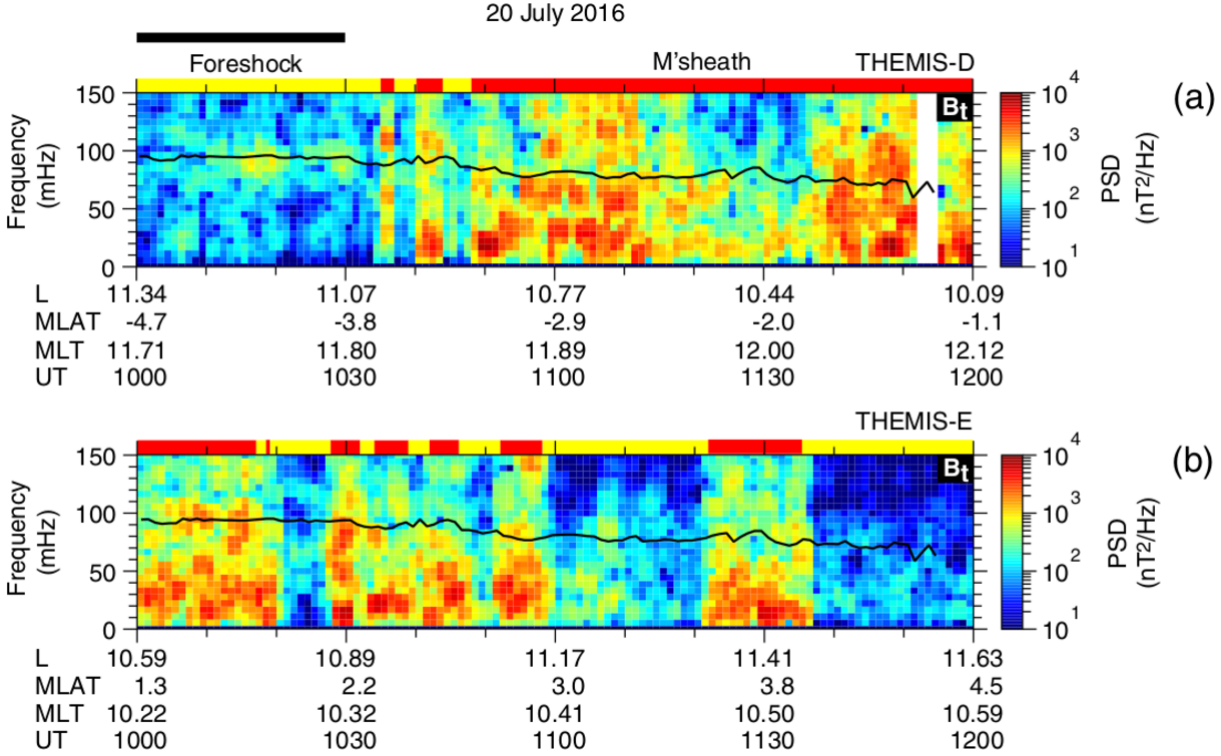
Dynamic power spectra of the total magnetic field fluctuations observed by the THEMIS-D (foreshock) and THEMIS-E (magnetosheath and foreshock) spacecraft during a period of quasi-radial IMF. The black curve indicates the expected foreshock wave frequency based on the Takahashi et al. (1984) formula. Figure adapted and reproduced with permission from Takahashi et al. (2021), copyright by the AGU

Takahashi et al. (2021) performed a detailed case study of a long-duration quasi-radial IMF interval which occurred on 20 July 2016, during the passage of large-scale solar wind structure, a magnetic cloud, at Earth. During this event, the THEMIS-A satellite was probing the dayside magnetosphere, while THEMIS-D and E were found alternatively in the foreshock and the quasi-parallel magnetosheath. At times, a clear enhancement in the total magnetic field wave power was observed in the magnetosheath at the same frequency as in the foreshock (see Fig. 15). This possible signature of transmitted waves was accompanied with other spectral peaks, suggesting the coexistence of multiple wave modes in the magnetosheath which renders challenging the analysis of the wave properties. Other intervals during the same event did not show any clear signal at the expected frequency, which might be due to the wave transmission occurring only in specific parts of the magnetosheath.

**Fig. 16:**
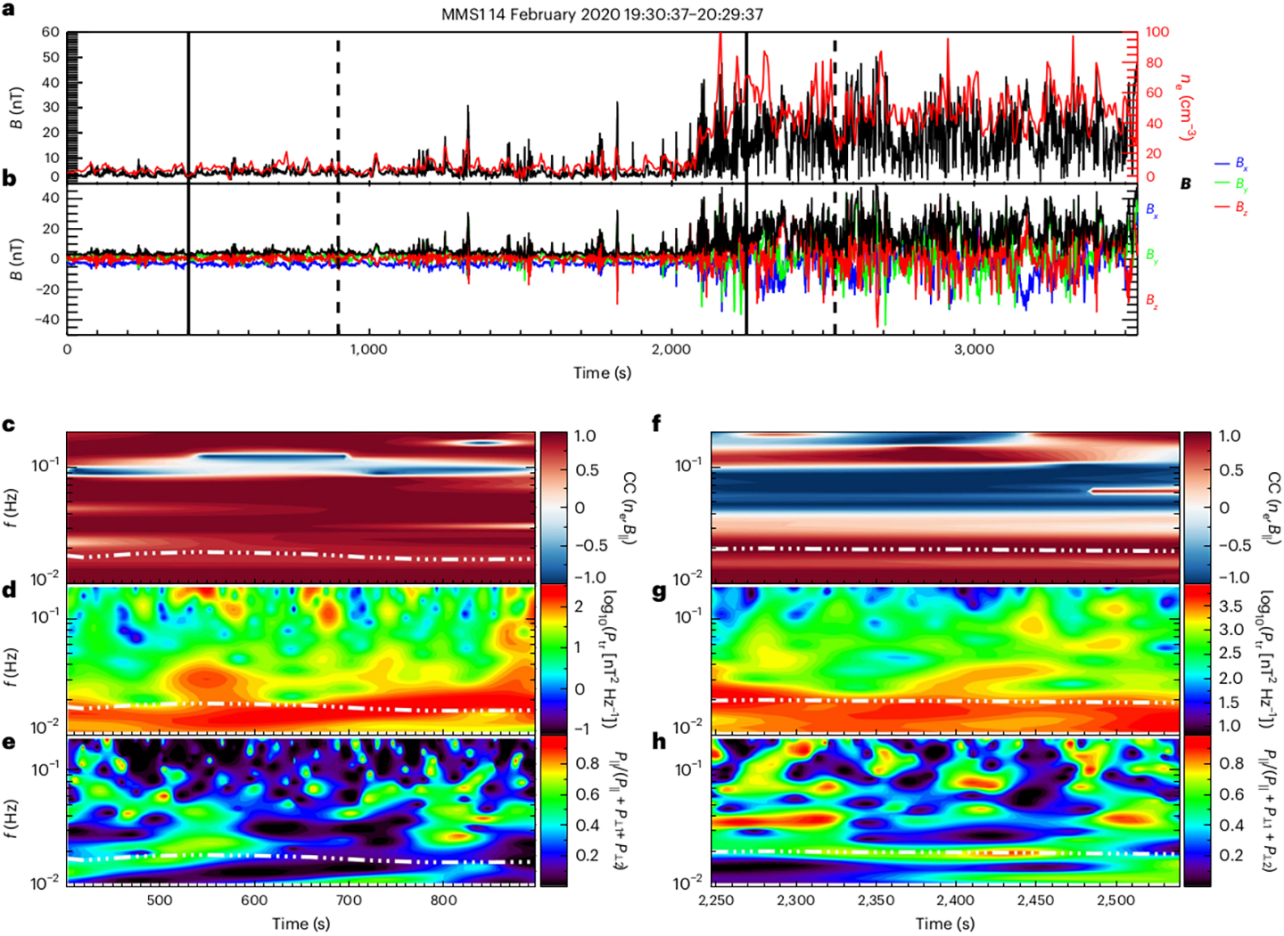
MMS observations during a one-hour interval when the spacecraft crossed from the foreshock into the subsolar magnetosheath. The top panels show (a) the magnetic field magnitude and electron density and (b) the magnetic field GSE components. The bottom part of the figure shows close-up of two intervals in the foreshock (left) and the magnetosheath (right), marked by the solid and dashed lines in the top panels. Shown in the bottom panels are the cross-correlation between the density and magnetic field strength fluctuations (c and f), the wavelet trace power spectra (d and g) and the compressibility of the magnetic fluctuations (e and h). The white dotted-dashed curves indicate the expected foreshock wave frequency based on the Takahashi et al. (1984) formula. From Turc et al. (2023)

As described in Sect. 3, Turc et al. (2023) identified fast-mode waves traversing the dayside magnetosheath in a global hybrid-Vlasov simulation, which could form the missing link between foreshock and dayside magnetosphere Pc3 waves. These waves were shown to be linearly polarised and to propagate earthward in the plasma rest frame. While they are easily detected from the global view provided by the simulation, their identification in time series mimicking actual spacecraft measurements is challenging, especially when moving away from the subsolar region. Based on these findings, Turc et al. (2023) searched for events when the MMS spacecraft were located within a few $R_{\mathrm{E}}$ from the Sun-Earth line and found a number of intervals with a clear signal at the expected foreshock wave frequency. One such event is illustrated in Fig. 16. A detailed analysis of the wave properties, using in particular the method described by Bellan (2016) which uses measurements of the magnetic field and plasma current density to obtain the wavevector, showed the presence of narrowband, earthward-propagating fast-mode waves, consistent with the numerical results.

These recent investigations suggest that the magnetosheath counterparts of foreshock waves may have eluded researchers for several decades because of the complex wave activity inside the magnetosheath, which contains a host of fluctuations in the ULF range coming from various sources, both internal and external. The fast-mode waves originating from the quasi-parallel shock do not necessarily appear as the dominant wave mode, especially further away from the subsolar region. This could for example explain why these waves were missed in the statistical survey by Narita et al. (2006), which focused on the dominant wave mode and was performed away from the subsolar point, due to the orbit of the Cluster spacecraft. In the light of these new findings, the existing datasets of spacecraft probing the subsolar region could be revisited to identify more events, opening new avenues for follow-up investigations of the magnetosheath wave properties in the context of foreshock wave transmission.

## Transmission of Pc3 Waves Through the Magnetopause

### Theory of Wave Transmission Through the Magnetopause

#### Magnetopause as a Tangential Discontinuity

The magnetopause is a current layer marking the outer boundary of Earth’s magnetosphere. It forms an obstacle that shields entry of the shocked solar wind into the magnetosphere. Likewise, the magnetopause acts as a perfect reflector to some magnetosheath ULF waves. At the same time, however, the magnetopause itself is a site of wave generation through either MHD or kinetic mechanisms. These magnetopause properties need to be considered when studying the transmission of 30-second waves into the magnetosphere. This section discusses the role of the magnetopause in transmitting and generating ULF waves.

The simplest theoretical approach to the magnetopause effect on ULF waves is to assume that the waves are MHD modes (the fast and slow magnetosonic modes and the Alfvén mode) and that the boundary is a tangential discontinuity (McKenzie 1970; Wolfe and Kaufmann 1975). For each incident wave specified by a frequency and a wave number, the properties of transmitted waves can be determined from a set of continuity conditions imposed on the boundary. Under the given assumptions, only magnetosonic modes can be transmitted because Alfvén waves propagate strictly along the magnetic field, which is parallel to the magnetopause. Therefore, in order for a 30-second wave generated in the foreshock to reach the magnetosphere, it must have a magnetosonic component in the magnetosheath. In addition, McKenzie (1970) found that only those waves close to normal incidence can be transmitted into the magnetosphere because of the vastly different Alfvén speeds on either side of the magnetopause.

Since the magnetopause is not a planar boundary, realistic MHD wave transmission through the magnetopause needs to be addressed by running 3D MHD simulations. Claudepierre et al. (2009, 2010) used the Lyon-Fedder-Mobarry global MHD code to study the magnetospheric response to solar wind dynamic pressure ($P_{\mathrm{dyn}}$) variations. MHD simulations cannot produce the ion foreshock or the 30-second waves excited in that region. However, the dynamic pressure variations generate compressional waves at the bow shock, and these waves can be treated as a proxy to ULF waves generated in the foreshock. The MHD simulations indicate, when the dynamic pressure variations have a broad spectrum, magnetospheric compressional waves are enhanced at the frequencies of the cavity mode waves or radially standing fast mode waves resulting from wave reflection at the inner (ionosphere) and outer (magnetopause) boundaries of the magnetosphere. The cavity mode waves exhibit discrete frequencies and spatial eigenmode structures, as noted earlier in linear MHD simulations assuming a dipole magnetospheric magnetic field (Lee and Lysak 1989). When the simulations is driven by monochromatic dynamic pressure variations, the corresponding magnetospheric fast mode waves are enhanced when one of the cavity mode frequencies matches the frequency of the externally applied disturbances. Cavity mode waves can be excited by various disturbances, and those directly associated with solar wind sources exhibit peak power on the day side.

An important source of ULF waves at the magnetopause is the Kelvin-Helmholtz instability (KHI), triggered by the velocity shear across the magnetopause. KHI-generated magnetopause surface waves propagate tailward and have typical periods within the Pc4–5 bands ($45 - 600$ s) (Lin et al. 2014). The instability has been theoretically studied assuming the magnetopause to be a tangential discontinuity (Southwood 1968). In the theory, the magnetic field on either side of the discontinuity provides stability to the boundary depending on the directions of the magnetic fields on the two sides of the boundary. In general, the equator is the most likely site of the instability because the magnetospheric magnetic field is the weakest there, providing the minimum stabilizing effect against tailward propagating disturbances on the magnetopause. The magnetosheath magnetic field varies in both direction and magnitude. If the direction is parallel to the equatorial plane, the magnetic field provides the strongest stabilizing effect against tailward propagating magnetopause disturbances. If the magnetosheath field is perpendicular to the equatorial plane (parallel or antiparallel to the magnetospheric field), it provides the least stabilization. From this consideration, we can understand that the velocity shear threshold for KHI depends on the magnetosheath magnetic field. If the direction of the magnetic fields varies with time, for example downstream of a quasi-parallel shock, the average angle between the fields can become small and the magnetopause may become more unstable (Nosé et al. 1995). MHD KHI may be common on the magnetopause flanks where the velocity jump is high but not at the magnetopause nose where the magnetosheath flow is stagnant (Wolfe and Kaufmann 1975; Merkin et al. 2013). When the finite thickness of the magnetosheath is imposed, the instability may be excited at discrete frequencies of waveguide modes (Mann et al. 1999). However, as the fundamental frequency of the waveguide modes has been estimated to be of the order of 1 mHz (Samson et al. 1992), it is unlikely that the modes resonate with and amplify the 30-second waves. While the KHI is generally associated with magnetospheric Pc4–5 waves (e.g. Mann et al. 2002; Kronberg et al. 2021), some studies show a positive correlation between Pc3 wave power and solar wind speed, suggesting that KHI can contribute to Pc3 wave activity inside the magnetosphere (e.g. Greenstadt et al. 1979, 1980; Odera 1986).

#### Magnetopause as a Gradient Layer

In reality, the magnetopause has a finite thickness and important wave phenomena can occur in the magnetopause gradient layer. A number of theoretical studies addressed kinetic properties of waves in this region. Observations near the magnetopause show that while magnetosheath ULF waves are mostly compressional, the waves become largely transverse when entering the magnetosphere. This abrupt change of polarization can be explained by a resonant mode coupling between the compressional waves and the Alfvén resonance (Belmont et al. 1995; Johnson and Cheng 1997; De Keyser et al. 1999), which leads to the generation of kinetic Alfvén waves (Johnson and Cheng 1997). This mode conversion was later confirmed observationally (Johnson et al. 2001). It has also been identified in global hybrid-Particle-in-Cell simulations describing the interaction of compressional disturbances generated in the foreshock with the magnetopause (Shi et al. 2013, 2017). These simulations suggest that the positively correlated density and magnetic field variations in the magnetosheath become anti-correlated in the magnetopause boundary layer, and that the kinetic Alfvén waves generated through mode conversion propagate poleward into the cusps and are carried tailward by the magnetosheath flow. We note that these waves generated or amplified in the gradient layer do not propagate across the background magnetic field and thus cannot account for compressional Pc3 waves detected deep in the magnetosphere or at low-latitude ground stations.

### Observational Evidence of Wave Transmission Across the Magnetopause

Wave transmission through the magnetopause can be inferred by comparing wave observations made across the boundary. In an early study, Wolfe and Kaufmann (1975) used Explorer-12 magnetometer data to evaluate the ratio of the wave power between the magnetosheath and magnetosphere. They found that the ratio near the subsolar point is consistent with wave transmission alone but the ratio away from the subsolar point required wave generation at the magnetopause. Using simultaneous ISEE-1 and -2 observations on either side of the magnetopause Greenstadt et al. (1983) found that the wave power of the magnetic field intensity in the 10–100 s period range is 10–1000 times lower in the magnetosphere. This result was attributed to magnetospheric waves being due to transmission (rather than production) of waves at the magnetopause.

It must be cautioned that ULF wave activity in the magnetosheath is very dynamic and contains mirror mode waves in the frequency range 10–100 mHz (Kaufmann et al. 1970) and other wave modes that are not included in the wave transmission theory. Extracting a single MHD wave mode, for example the fast mode, from spacecraft data is often a difficult task not only for the magnetosheath but also for the magnetosphere waves (Hartinger et al. 2013a). Another complicating factor is the presence of magnetopause surface modes (Archer et al. 2019), which have been reported at frequencies below 10 mHz but could extend to the Pc3 band if excited at high-order harmonics.

### Wave Transmission at High Latitudes

**Fig. 17:**
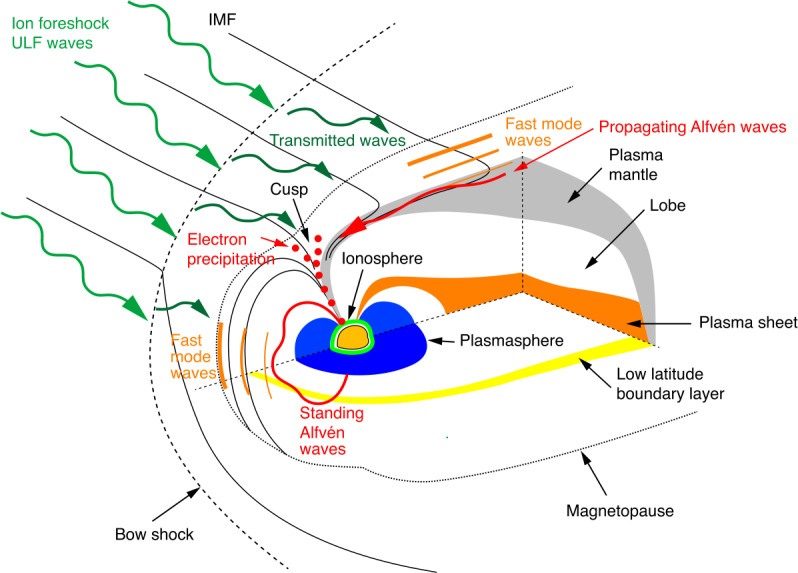
Illustration of wave entry from different locations on the magnetosheath-magnetosphere boundary. This is a composite of Fig. 1.18 of Kivelson and Russell (1995) and Fig. 7 of Chugunova et al. (2004)

The wave transmission mechanisms described above apply to the subsolar magnetopause. However, foreshock wave energy may reach the inner magnetosphere and the ground via various paths, as illustrated in Fig. 17. In addition to the subsolar magnetopause, possible wave entry sites include the cusp, mantle, and lobe regions of the magnetosphere. Chugunova et al. (2004) compared the Pc3 pulsation power on the ground observed at different magnetic latitudes ($\lambda _{m}$), namely $\lambda _{m}$ ∼72^∘^, 80^∘^, and 87^∘^. The authors noted that the power enhancement occurs at noon at $\lambda _{m}$ ∼72^∘^ but at earlier local times at the two higher latitudes. This result led the authors to suggest that magnetosheath waves of foreshock origin penetrate through the cusp, accounting for the observations at $\lambda _{m}$ ∼72^∘^, and the morning flank of the magnetosphere in the region of open field lines (mantle/tail lobe), accounting for the observations at $\lambda _{m}$ ∼80^∘^ and $\lambda _{m}$ ∼87^∘^. In related studies, De Lauretis et al. (2010) and Francia et al. (2012) argue that the compressional waves penetrate into the tail lobes and can couple to Alfvén waves on field lines near the outer boundary of the lobes/magnetosheath interface. These Alfvén waves, being excited on open field lines, will not exhibit harmonics unlike Alfvén waves excited on closed field lines.

Fast mode waves may not be the only carrier of ULF wave signals into the magnetosphere. Engebretson et al. (1991a) proposed a mechanism that does not require inward-propagating fast mode waves: the ionospheric transistor model. In this model, variations of the momentum or plasma pressure in the magnetosheath that originate from foreshock ULF waves modulate precipitation of electrons along the cusp/cleft/low-latitude boundary layer (LLBL) field lines. The cusp is the region in which the magnetosheath particle have direct access to the polar ionosphere, and the cleft is the ionospheric region that maps to the LLBL (see Fig. 17). Lanzerotti et al. (1981) had speculated on the role of the ionosphere in connecting cusp-latitude disturbances to lower latitudes but did not offer specifics. In the transistor model, the precipitation modulates the ionospheric conductivity, which leads to modulation of the region 1 (connected to the magnetopause) and region 2 (connected to the ring current) currents. If the currents are longitudinally extended, they produce azimuthal perturbations of the magnetic field away from the latitudes of strong currents.

## Magnetospheric Pc3 Waves

Inside the magnetosphere, ULF waves are generally categorised based on their period (or frequency), using the classification proposed by Jacobs et al. (1964) (see Table 1 in Sect. 1). This classification separates continuous pulsations (Pc), with a regular pattern, from irregular pulsations (Pi), characterised by irregular wave forms and generally connected with magnetospheric disturbances such as substorms. Pc waves are then further divided into 5 categories, corresponding to 5 period bands. Foreshock waves, with their typical period around 30 s, are associated with magnetospheric waves in the Pc3 band ($10-45$ s), sometimes extending into the Pc4 band ($45-150$ s). It is important to note that the Pc classification solely takes into account the wave period, and does not distinguish between different wave modes or different wave sources. Magnetospheric waves in the Pc3 band can thus have multiple origins.

**Fig. 18:**
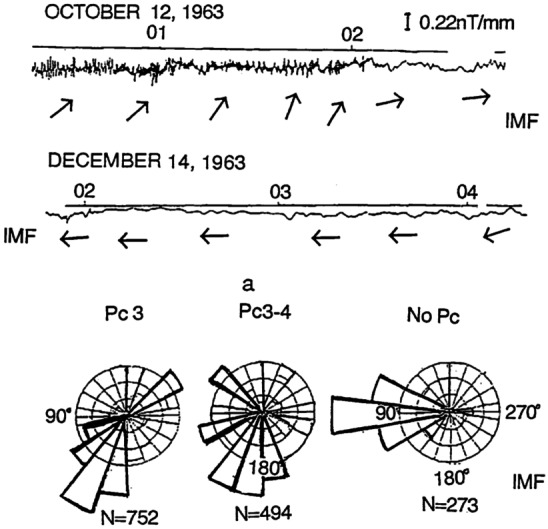
(a) Magnetic pulsations recorded at the Petropavlovsk Kamchatsky magnetometer station on two different days. The arrows indicate the direction of the IMF in the ecliptic plane, with the Sun in the upward direction. (b) Occurrence of Pc3 pulsations as a function of the IMF cone angle. Figure reproduced with permission from Troitskaya (1994), copyright by the AGU

Pc3 waves are routinely observed in the dayside magnetosphere, ionosphere and on the ground (e.g. Raspopov and Lanzerotti 1976; Arthur et al. 1977; Troitskaya 1994; Howard and Menk 2005; Engebretson et al. 2006; Heilig et al. 2007; Bier et al. 2014; Murphy et al. 2020). It was noted as early as the 1960s that the level of Pc3 wave activity depends on the IMF direction (Bol’shakova and Troitskaya 1968). Numerous studies, based on both spacecraft and ground-based measurements, have thereafter confirmed that Pc3 wave power is enhanced when the IMF cone angle is low, that is, during times when the foreshock extends upstream of the dayside magnetosphere (e.g. Troitskaya et al. 1971; Greenstadt and Olson 1976, 1977; Arthur et al. 1977; Vero and Hollo 1978; Takahashi et al. 1981; Wolfe et al. 1985; Engebretson et al. 1987; Chi et al. 1994). An example of the distribution of Pc3 wave occurrence as a function of the IMF cone angle recorded by ground magnetometer stations is displayed in Fig. 18. At the same time, clear evidence was also found that the wave frequency is controlled by the IMF strength, further suggesting an external origin for those waves (Troitskaya et al. 1971; Gul’yel’mi 1974; Vero and Hollo 1978; Pliasova-Bakunina et al. 1978; Takahashi et al. 1984; Yumoto et al. 1984; Troitskaya and Bol’Shakova 1988). We refer the interested reader to the comprehensive historical reviews of Pc3 wave research by Troitskaya (1994) and Hughes (1994).

The IMF control of the Pc3 wave properties led Troitskaya et al. (1971) to first propose that magnetospheric Pc3 waves are connected to foreshock waves, or rather *upstream waves*, as those waves are generally termed in magnetospheric studies. However, there is also evidence suggesting that part of the magnetospheric Pc3 wave activity may be unrelated to the foreshock. For example, Lessard et al. (1999) found Pc3 wave occurrence peaking in the afternoon sector, which is inconsistent with the average foreshock position, on the dawn flank because of the IMF Parker spiral orientation. One may however speculate that the data used in the Lessard et al. (1999) statistical study could have been obtained during non-Parker spiral IMF orientations, resulting in foreshock wave power being larger in the dusk magnetosphere. There are also reports of intense Pc3 waves on the nightside (Shi et al. 2018), which is again incompatible with a foreshock origin. In the next subsections, we will first focus on the wave propagation and coupling mechanisms relevant for Pc3 waves originating in the foreshock, while other sources of Pc3 waves will be discussed in Sect. 6.2. We will then review the global distribution of Pc3 wave activity in the magnetosphere in Sect. 6.3 and briefly introduce how these waves can be used for magnetoseismology in Sect. 6.4.

### Magnetospheric Pc3 Waves of Foreshock Origin

#### Pc3 Waves in the Equatorial Magnetosphere

**Fig. 19:**
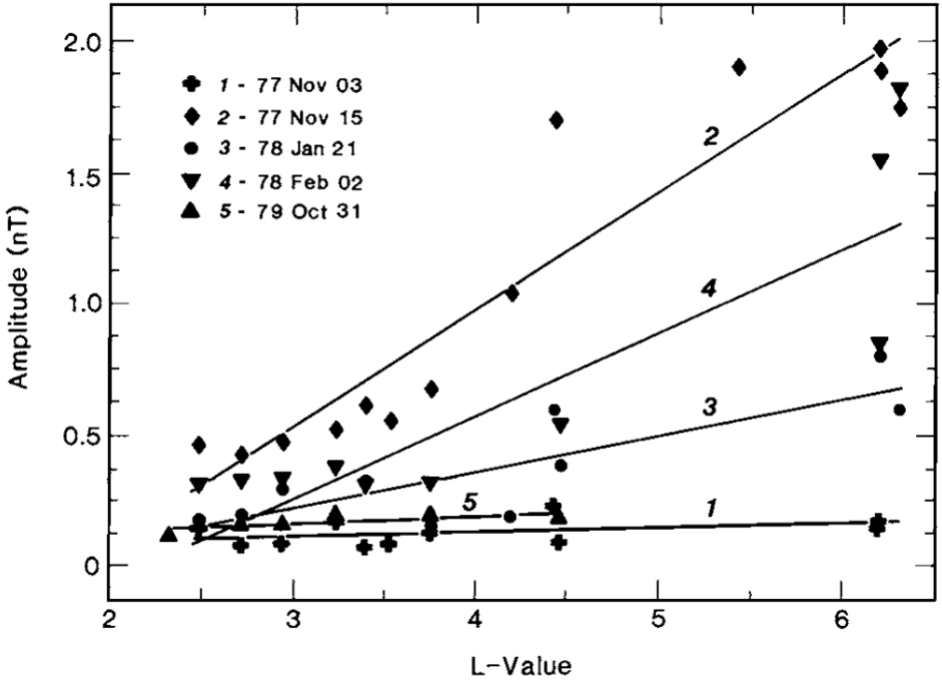
Amplitude of the Pc3 fluctuations recorded by ground-based magnetometers as a function of the $L$ value of the station, for 5 different events. The Pc3 wave amplitudes are obtained by integrating under the peak of the wave power spectra. The wave amplitude tends to increase when moving to larger $L$ values. Figure reproduced with permission from Odera et al. (1994), copyright by the AGU

As described in Sect. 5, in the equatorial entry scenario, foreshock waves enter the magnetosphere as compressional, fast-mode waves in the Pc3 band. These waves propagate tailwards, transporting the Pc3 wave energy across the geomagnetic field lines, and are observed both in space and on the ground (e.g. Yumoto et al. 1985; Odera et al. 1991, 1994; Chi et al. 1994; Takahashi et al. 1994). On the dayside, the Pc3 compressional wave power tends to decrease with geocentric distance, or latitude when observed from the ground, consistent with a wave origin external to the magnetosphere, as illustrated in Fig. 19 (Odera et al. 1991, 1994). Engebretson et al. (1987) showed for example that there is a higher probability of detecting monochromatic compressional waves beyond $L = 8$ (where a given $L$ or $L$-shell corresponds to the set of magnetospheric field lines which intersect the equatorial plane at this distance, measured in Earth radii from Earth’s centre). This suggests an attenuation of the wave amplitude as they propagate earthward. In some cases, compressional Pc3 waves have been observed to propagate all the way to the midnight sector (Ponomarenko et al. 2010; Takahashi et al. 2016; Villante and Tiberi 2016; Yagova et al. 2017). Overall, the compressional Pc3 wave power is enhanced near the equator, bringing support to the waves entering through the equatorial magnetopause (Takahashi and Anderson 1992; Heilig et al. 2007).

**Fig. 20:**
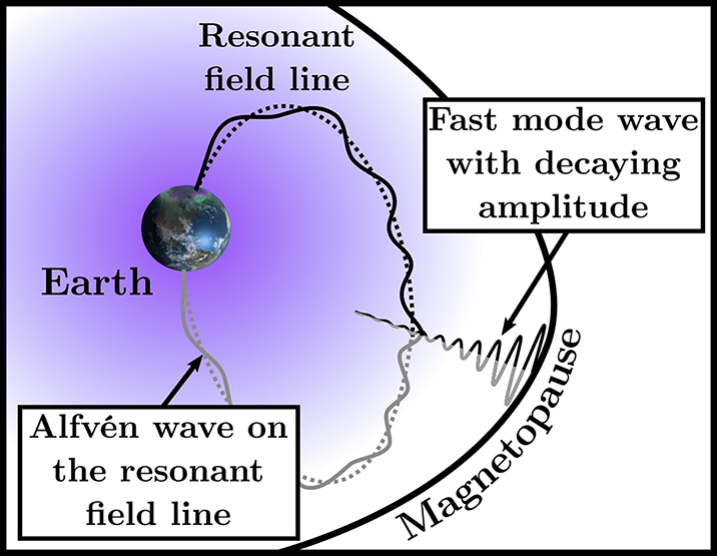
Schematic of the field line resonance process driven by fast-mode waves propagating from the magnetopause inwards

When compressional MHD waves travel in a non-uniform plasma, where the Alfvén speed varies, they can couple with standing Alfvén waves via the field line resonance mechanism. This wave coupling process enables the transfer of energy from an MHD fast-mode wave, which can propagate transverse to the field lines, to an Alfvén wave, propagating purely along the magnetic field, at the position where the resonance condition $\omega = k_{\parallel}v_{\mathrm{A}}$ is met (with $k_{\parallel}$ the wave vector parallel to the magnetic field and $v_{\mathrm{A}}$ the Alfvén velocity). The field line resonance process is illustrated in Fig. 20. The presence of standing Alfvén waves on magnetospheric field lines was first proposed by Dungey (1955) to explain the long-period, regular oscillations observed by ground-based magnetometers. The concept of field line resonance was then developed by Chen and Hasegawa (1974) and Southwood (1974), providing an explanation for the source of the standing oscillations. The standing waves are associated with an azimuthal magnetic field variation and are termed toroidal waves. These waves have low azimuthal mode numbers (in the cold plasma approximation, the waves are axisymmetric and $m = 0$) and are excited by external perturbations with a large azimuthal extent (Takahashi 2016). More detailed reviews of the theory of field line resonances can be found for example in Hughes (1994), Glassmeier et al. (1999), Elsden et al. (2022).

Due to their relatively short periods, Pc3 waves tend to couple with field line resonances at midlatitudes, between $35^{\circ}$ and $50^{\circ}$, where they match the fundamental eigenfrequencies of the field lines (Waters et al. 1991; Heilig et al. 2013). In the outer magnetosphere, they can excite high harmonic standing oscillations, which then appear at higher latitudes on the ground (Yumoto et al. 1985; Howard and Menk 2005).

#### Pc3 Waves at High Latitudes

**Fig. 21:**
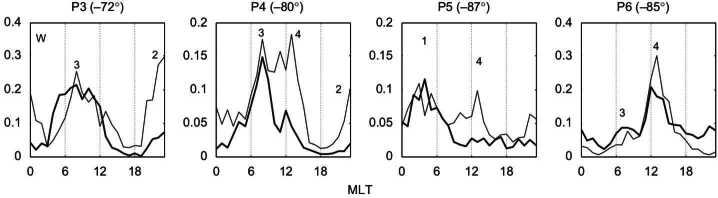
Diurnal variation of Pc3 (thick line) and Pc4 (thin line) hourly pulsation energy W [$\mathrm{nT}^{2}/\mathrm{mHz}$] of the magnetic field H component measured at four high-latitude ground stations in Antarctica. The latitudes of the stations in corrected geomagnetic coordinates are indicated on top of each of the panels and correspond to auroral latitudes (P3), cusp latitudes (P4 and P6) and polar cap (P5). Reproduced with permission from Pilipenko et al. (2008), copyright by Elsevier

Ground-based observations have provided extensive evidence that Pc3 wave activity is a common occurrence at auroral latitudes, near the cusp and in the polar cap. The wave power distribution as a function of magnetic local time strongly varies with latitude, suggesting the existence of several populations of Pc3 waves (see Fig. 21 and Chugunova et al. 2006; Pilipenko et al. 2008). At auroral latitudes, the Pc3 wave power peaks in the morning sector, consistent with the propagation into the ionosphere of high harmonic standing waves driven by field line resonances in the outer magnetosphere. In contrast, at cusp latitudes, Pc3 wave power is concentrated near local noon, suggesting wave entry through the dayside cusp (Bolshakova and Troitskaia 1984; Matsuoka et al. 2002; Chugunova et al. 2006; Pilipenko et al. 2008; Liu et al. 2008). At even higher latitudes, in the polar cap, the wave power is concentrated in the early morning hours (3–5 MLT) (Chugunova et al. 2006; Pilipenko et al. 2008). Despite these contrasted MLT distributions, there is strong evidence that Pc3 wave power in the high-latitude regions of the magnetosphere has its source in the foreshock. The high-latitude Pc3 waves show similar dependencies on solar wind and IMF conditions as their low-latitude counterparts, with their occurrence controlled by the IMF cone angle and the solar wind velocity (Engebretson et al. 1986; Ponomarenko et al. 2002; Villante et al. 2000; Pilipenko et al. 2008). It was also noted that Pc3 pulsations tend to be suppressed during extreme solar wind driving, possibly because the background noise becomes higher during such times, preventing the detection of the foreshock-driven Pc3 waves (Pilipenko et al. 2008). However, enhanced narrowband Pc3 wave power in the cusp and the polar cap cannot be explained by fast-mode waves coupling with standing waves through the field line resonance mechanism because the open field line geometry prevents such resonances. Despite their common source in the foreshock, the waves follow another pathway to reach the high-latitude magnetosphere.

In addition to its concentration near local noon, Pc3 wave activity near the cusp also differs from that at lower latitudes in that the wave packets tend to have a shorter coherence and a large azimuthal wave number, both suggesting waves with smaller scales than their equatorial latitude counterparts (Szuberla et al. 1998; Baker et al. 1998; Liu et al. 2009a). These differences further hint at a different origin of the waves.

Pc3 wave activity observed near the cusp can be explained via the ionosphere transistor model (Engebretson et al. 1991a), which involves the modulated precipitation of previously-trapped electrons on the equatorward edge of the cusp due to magnetosheath fluctuations impinging on the magnetopause. This modulated precipitation causes periodic variations of the ionospheric conductivity and thus of the ionospheric currents. The currents would thus form a source of narrowband wave energy in the Pc3 range, launching transverse magnetic field oscillations in the Pc3 range. The resulting transverse waves could then propagate towards the outer magnetosphere along the field lines, and could thus be responsible for the harmonically-structured oscillations detected at large $L$-shells (Engebretson et al. 1987, 1991a). As a result, the compressional and toroidal wave powers are independent from each other in this scenario, which may be used to distinguish between the different entry mechanisms (e.g. Anderson and Engebretson 1995). In a statistical survey of high-latitude ground-based observations, Engebretson et al. (1994a) found that the modulated precipitating particle fluxes inferred from the variations in optical auroral emissions were in quantitative agreement with the generation of Pc3 waves measured simultaneously, bringing further support to the ionospheric transistor model. The precipitating particles had characteristics of an energetic trapped population, suggesting that they originate from closed field lines, rather than cusp precipitation (Engebretson et al. 1994b). The exact source of the modulated particle precipitation remains however unclear.

Another possible source for Pc3 waves in the cusp region is through leakage of MHD turbulence from the high-altitude cusp towards the ionosphere (Engebretson et al. 2006). The turbulence at the interface between the magnetosheath and the high-altitude cusp is however typically broadband in nature, whereas cusp Pc3 waves display narrowband signatures (Matsuoka et al. 2002; Ponomarenko et al. 2002). To reconcile these contradicting observations, Pilipenko et al. (1999) proposed a theoretical model in which compressional waves in the high-altitude cusp are converted into narrowband Pc3 Alfvén waves propagating to the ground. In this model, the cusp acts both as a waveguide for the compressional fluctuations and as a resonator, amplifying selectively those fluctuations in the Pc3 band. Because of the local mode conversion within the cusp, this mechanism will result in a lack of coherence between Pc3 waves observed at conjugate locations in the two hemispheres, contrasting with Pc3 waves associated with field line resonances at lower latitudes in the magnetosphere. This lack of coherence between conjugate observations at cusp latitudes has been reported by Wolfe et al. (1990).

It is still unclear which of these two proposed scenarios operates in the cusp, as observations provide indirect evidence supporting each of them. For example, Matsuoka et al. (2002) found high coherence between magnetosheath fluctuations and Pc3 waves on the ground, despite the broadband nature of magnetosheath oscillations, which favours the hypothesis of magnetic field disturbances transmitting through the cusp. On the other hand, Yeoman et al. (2012) report that Pc3 wave power observed by ground-based observatories on Svalbard, at cusp latitudes, peaks near the equatorward edge of the cusp and its intensity tracks the motion of the cusp. The source of Pc3 wave activity near the dayside cusp thus remains an open area of research.

The observations of narrowband Pc3 waves in the polar cap were rather unexpected, since field line resonances cannot occur on open field lines (e.g. Chugunova et al. 2004; Engebretson et al. 2006). Yet multiple studies have confirmed the presence of these waves, both in situ in the magnetotail lobes and on ground-based observatories at very high latitudes, in particular in Antarctica (Chugunova et al. 2004, 2006; Pilipenko et al. 2008; De Lauretis et al. 2010; Villante et al. 2011; Francia et al. 2012; Regi et al. 2013, 2014). The processes responsible for their transmission from the magnetosheath into the magnetotail lobes are still an open question. It has been proposed that fast-mode disturbances originating from the foreshock could directly transmit into the lobes. There, mode conversion into Alfvén waves could occur, launching transverse waves propagating earthward along the field lines which would bring the wave energy into the polar cap ionosphere (Engebretson et al. 2006; Pilipenko et al. 2008; Regi et al. 2013). Another proposed scenario is that Alfvénic disturbances could propagate along reconnected field lines, thereby transmitting directly from the magnetosheath into the magnetotail lobe (Engebretson et al. 2006; Pilipenko et al. 2008). Although observations in the magnetotail suggest the presence of both compressional and transverse waves (e.g. Regi et al. 2013), it should be noted that no theory has yet been developed for mode conversion on open field lines (Engebretson et al. 2006).

**Fig. 22:**
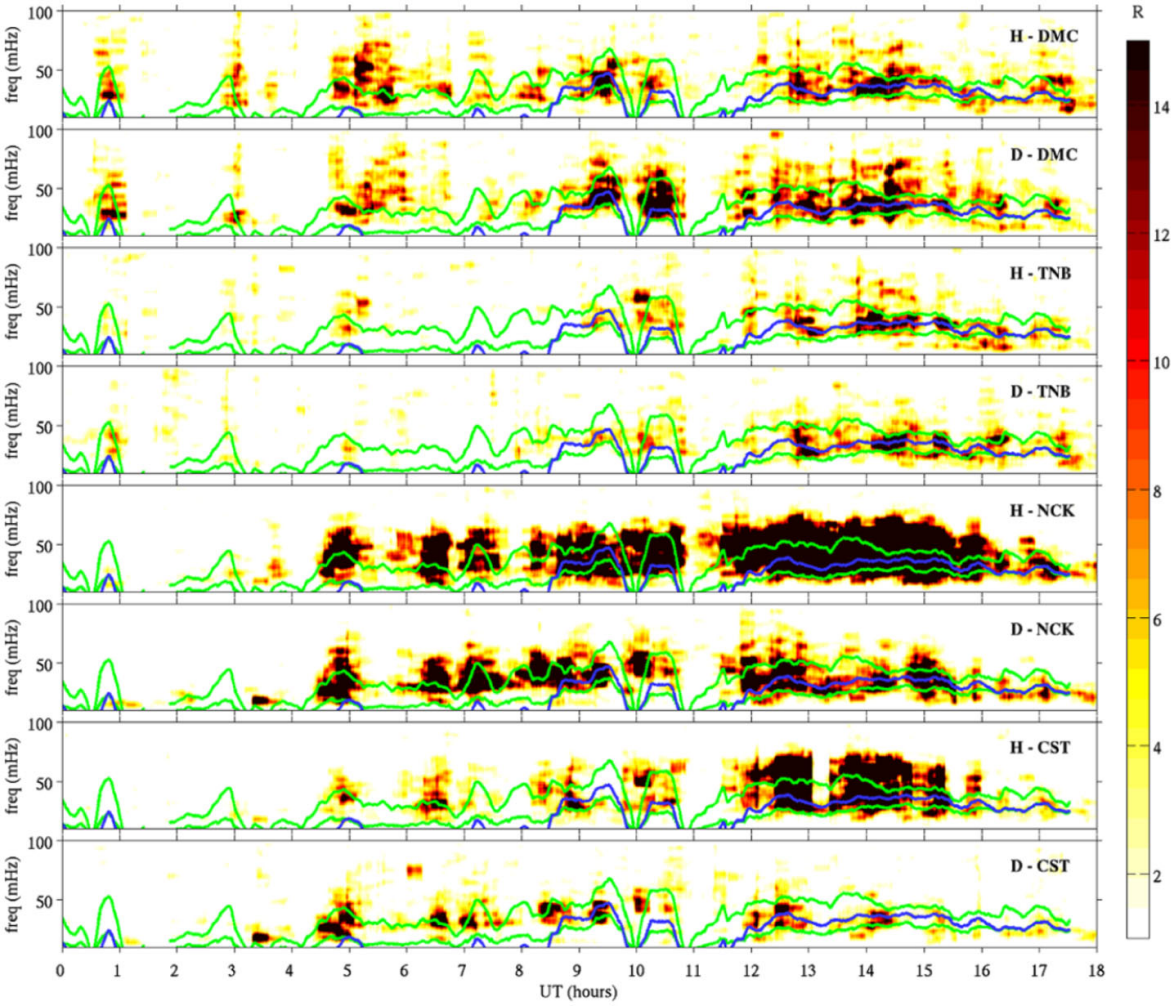
Simultaneous observations of Pc3 waves at polar and low latitudes, at the expected foreshock wave frequency (blue line). The panels show the dynamic signal-to-noise ratio R of the fluctuations of the magnetic field horizontal components (H and D) recorded on 15 February 2009 at different magnetometer stations: Dome C (DMC) and Terra Nova Bay (TNB), in Antarctica, on open field lines, and Nagycenk (NCK; Hungary) and Castello Tesino (CST; Italy), on low-latitude, closed field lines. From Regi et al. (2014)

Overall, large statistical surveys and detailed case studies of Pc3 wave activity inside the magnetosphere concur to indicate that multiple entry mechanisms can operate at the same time, enabling the transmission of foreshock waves into the equatorial magnetosphere, the cusp and the polar cap all at once (Engebretson et al. 1987; Anderson and Engebretson 1995; Chugunova et al. 2006; Heilig et al. 2007; Regi et al. 2014). This is illustrated in Figs. 22 and 26, which show intense Pc3 wave power both near the equator and at high latitudes, during a single event (Fig. 22) and based on a statistical survey of measurements in the topside ionosphere (Fig. 26). Though the exact pathways and their relative importance are still heavily debated, especially at high latitudes, there is a consensus that these different Pc3 wave populations have a common origin in the foreshock.

#### Propagation to the Ground

This section describes how waves originating from the foreshock propagate to the $L < 4$ region (plasmaspheric latitudes) on the ground.

**Fig. 23:**
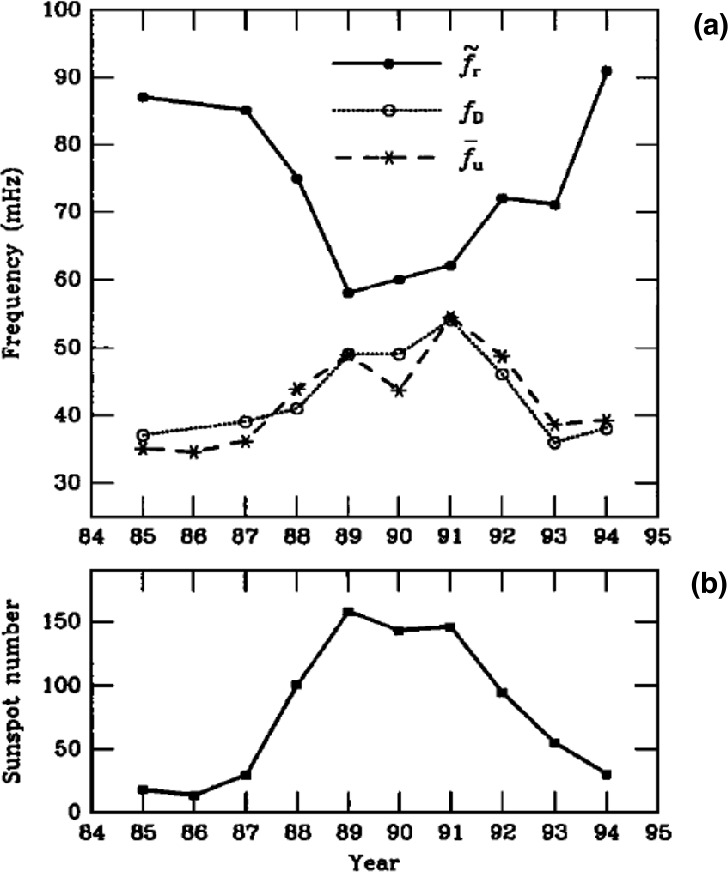
Long-term statistics of the frequencies of ground magnetic pulsations detected $L = 1.6$. (a) Dominant frequencies ($\tilde{f_{r}}$ and $f_{D}$) and a model foreshock wave frequency ($\bar{f_{\mathrm{u}}}$) derived from the annual mean of the magnitude of IMF. (b) Sunspot number. Figure reproduced with permission from Vellante et al. (1996), copyright by the AGU

Ground magnetic pulsations contain at least two frequency components, one attributed to local field line resonance (FLR) and the other to global compressional oscillations directly driven by upstream sources such as foreshock waves (see, for example, Tanaka et al. 2004). Figure 23 shows a good example of these components ($\tilde{f_{r}}$ and $f_{D}$) detected at low-latitude ($L=1.6$). $\tilde{f_{r}}$ is detected in the $H$ (horizontal northward) component and is attributed to local fundamental FLR. The exact value of this local frequency varies with the solar cycle: $\tilde{f_{r}}$ becomes lower when the sunspot number is higher because an elevated solar activity leads to feeding of more ionospheric O^+^ ions to the magnetosphere, resulting in lower FLR frequencies. $f_{D}$ is detected both in the $H$ and $D$ components (where $H$ and $D$ are the magnetic field horizontal components, oriented towards magnetic north and eastward, respectively) and is attributed to upstream waves because $f_{D}$ matches the prediction ($\bar{f_{\mathrm{u}}}$) based on an empirical model of the foreshock wave frequency $\bar{f_{\mathrm{u}}}$ (mHz) ∼ 6$B_{\mathrm{IMF}}$ (nT) where $B_{\mathrm{IMF}}$ is the magnitude of the interplanetary magnetic field (Vellante et al. 1996).

**Fig. 24:**
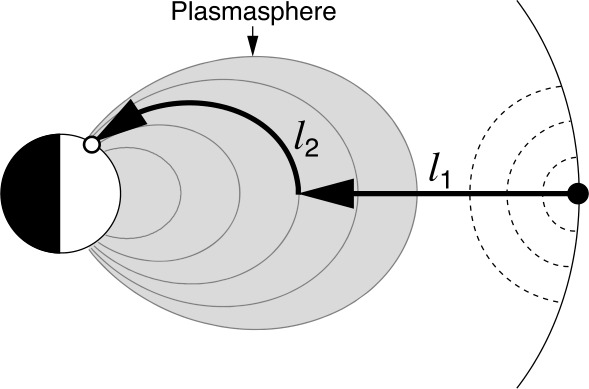
Schematic of propagation of an MHD impulse from a point source located on the magnetopause. Figure reproduced with permission from Takahashi and Heilig (2019), copyright by the AGU

Many features of the ground magnetic pulsations can be explained using a model developed by Tamao (1964) for propagation of magnetic impulses induced by sudden changes in the solar wind dynamic pressure (Ponomarenko et al. 2005; Takahashi and Heilig 2019). Figure 24 illustrates how a compressional impulse originating from a point source on the magnetopause (black filled circle) reaches a point on the ground (open circle) along two different paths. On one path, the impulse propagates in the fast mode (arcs drawn with dashed lines) all across the magnetic field. This path results in little time delay between ground observatories located at different latitudes. Because the impulse reaches the topside ionosphere as a fast mode wave the major axis of polarization of magnetic field perturbation does not rotate through the ionosphere (Nishida 1964; Kivelson and Southwood 1988). The fast mode propagation explains $D$ component pulsations, which exhibit little phase delay between different latitudes (Takahashi and Heilig 2019; Takahashi et al. 2021).

On the other path (Tamao path), the impulse propagates along two segments, $l_{1}$ and $l_{2}$. On segment $l_{1}$, the impulse propagates in the fast mode while on segment $l_{2}$ the impulse propagates in the Alfvén mode. The two modes couple in the equatorial region. The impulse on the Tamao path is detected on the ground with a time delay given by

1 $$ {T_{\mathrm{Tamao}}} = \int _{l_{1}}\frac{ds}{V_{\mathrm{f}}} + \int _{l_{2}}\frac{ds}{v_{\mathrm{A}}}, $$

where $V_{\mathrm{f}}$ and $v_{\mathrm{A}}$ are the fast mode and Alfvén mode velocities, respectively. In a cold plasma, these velocities are equal and ${T_{\mathrm{Tamao}}}$ depends on the length of segment $l_{2}$ and the Alfvén velocity on it. In regions where the mass density monotonically decreases with $L$ (for example in the plasmasphere), ${T_{\mathrm{Tamao}}}$ is an increasing function of $L$. This means that an impulse propagating along the Tamao path arrives at the ground earlier at lower $L$. Because of the Alfvénic nature of the impulse along $l_{2}$ the major axis of polarization rotates by 90^∘^ when passing the ionosphere (Nishida 1964; Kivelson and Southwood 1988). Accordingly, if the Alfvén wave on $l_{2}$ is azimuthally polarized, the corresponding ground magnetic field perturbation has a strong $H$ component.

**Fig. 25:**
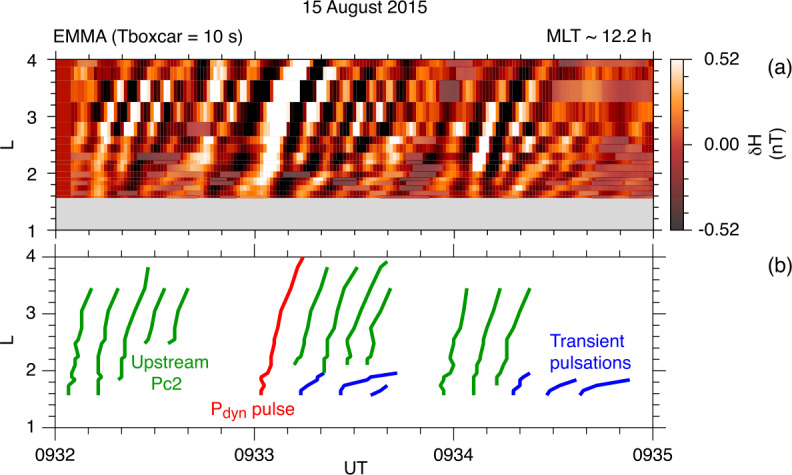
$L$ versus structure of magnetic field perturbation observed by the European quasi Meridian magnetometer array (EMMA). Figure reproduced with permission from Takahashi and Heilig (2019), copyright by the AGU

Figure 25 shows observations in support of the Tamao model. The $L$ versus time structure of the perturbation in the $H$ component (Fig. 25a) reveals regularly spaced ridges extending from $L < 2$ to $L = 4$ with a slope that does not change with time (the green lines labeled “upstream Pc2” in Fig. 25b). The slope is the same as that for the ridge produced by an isolated solar wind dynamic pressure pulse (the red line labeled “$P_{\mathrm{dyn}}$ pulse” in Fig. 25b) and matches the prediction of the Tamao model very well (figure not shown). The regular spacing is attributed to foreshock waves having frequencies (∼ 130 mHz) higher than usual because of a very high $B_{\mathrm{IMF}}$ value (∼ 20 nT).

An additional feature is seen at $L < 2$. In this region, there are ridges with slopes that changes with time (the blue lines in Fig. 25a labelled “transient pulsations”). This structure is attributed to ringing of field lines at the local eigenfrequencies. The same phenomenon occurs at higher latitudes as well (Poulter and Nielsen 1982). Cross-phase analysis of the ringing observed at latitudinally separated stations results in a cross-phase spectrum indicating the FLR frequency at the midpoint latitude (Waters et al. 1991). The FLR frequency and the foreshock wave frequency match at some $L$, and at that point the $H$ component exhibits a strong FLR oscillation. The ionospheric rotation of the polarization axis is also observed for this type of Alfvén waves (Vellante et al. 2004; Ndiitwani and Sutcliffe 2009).

### Other Sources of Pc3 Waves in the Magnetosphere

Within the magnetosphere, ULF waves are known to be triggered by external, solar wind-induced perturbations, which propagate within and couple to various modes of perturbations of the magnetosphere. For example, compressional waves driven by interplanetary shocks or pressure pulses impinging upon the magnetopause are known to couple to the shear Alfvén mode of standing ULF waves accross all ranges of frequencies through the FLR mechanism (Lysak and Lee 1992; Hudson et al. 2004; Eriksson et al. 2006; Sarris et al. 2009b,a; Liu et al. 2009b; Xie et al. 2023). Specifically for Pc3 waves, Waters et al. (1991), using ground-based measurements, found that resonances and harmonics in the Pc3 range of frequencies can be observed virtually every day, and confirmed that their frequencies are in excellent agreement with model calculations of standing toroidal field line resonances. Sarris et al. (2010) also showed a case of field line resonances that were excited globally in conjunction with a sharp increase in the upstream solar wind density, as observed at different L-shells, simultaneously with different amplitudes and at different frequencies in the Pc3 to Pc5 range.

The Kelvin-Helmholtz instability (KHI) develops at the flank (dawn and dusk) magnetopause due to the velocity shear between the magnetosheath flow and the magnetospheric plasma (e.g. Hasegawa et al. 2004; Kavosi and Raeder 2015; Masson and Nykyri 2018). The KHI generates magnetopause surface waves, which can in turn couple with FLR (Chen and Hasegawa 1974; Southwood 1974). A faster solar wind speed is more favourable to the growth of the KHI and consequently, the positive correlation between solar wind speed and magnetospheric Pc3 wave occurrence suggests that the KHI could be an important source of Pc3 waves (e.g. Singer et al. 1977; Greenstadt et al. 1979; Wolfe 1980; Takahashi et al. 1981). We note however that detailed analyses of the Pc3 wave properties such as those performed by Mier-Jedrzejowicz and Southwood (1981), Morrison (1991) and Howard and Menk (2005) indicate that the observed wave characteristics are inconsistent with a generation by the KHI and suggest a source in the foreshock. Overall, magnetospheric ULF wave activity driven by the KHI tends to be observed at lower (Pc4–5) frequencies (e.g. Mann et al. 2002; Kronberg et al. 2021).

Finally, another generation mechanism for Pc3 waves has been speculated to be associated with the dynamics of the magnetotail during active geomagnetic times, in particular at midlatitudes and in the nightside ionosphere: Using 7 years of SuperDARN data, (Shi et al. 2018) showed that Pc3 (and Pc4) events at midlatitudes were seen predominantly on the nightside with an occurrence peak in the premidnight region, in conjunction with a sharp increase of the AE index, a decrease of SYM-H index and southward IMF. These elements indicate an association with magnetotail dynamics such as substorms. The fact that substorms as well as more localised geomagnetic disturbances can contribute to Pc3 wave activity on the nightside was also pointed out by (Yagova et al. 2017). Using numerical simulations, Yamakawa et al. (2020) showed that drift-bounce resonance with ions injected into the ring current during substorms can excite poloidal Pc3 waves with high mode number in the dusk sector. In a recent statistical survey using both space-borne and ground-based data, Li et al. (2023) have shown that Pc3 wave power tends to be enhanced during the main phase of geomagnetic storms, together with ULF wave power in the Pc4 and Pc5 frequency bands. This further supports a link between geomagnetic activity and Pc3 waves.

### Global Distribution of Pc3 Waves in the Magnetosphere

**Fig. 26:**
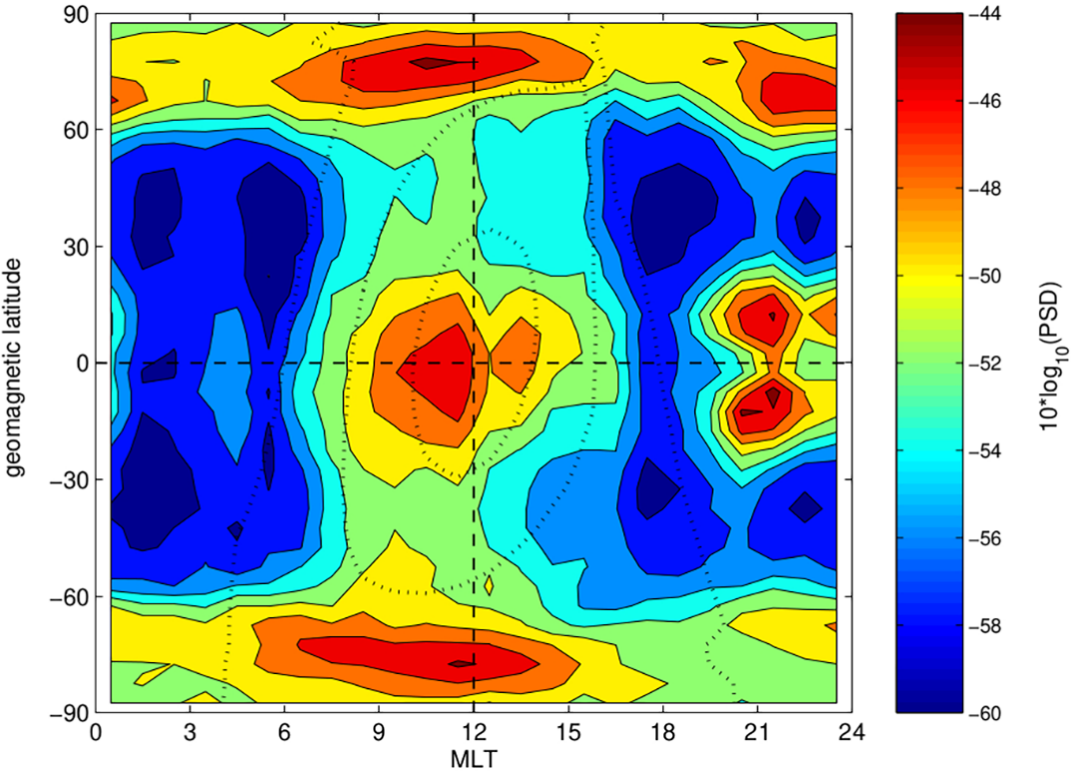
Distribution of the mean Pc3 compressional wave power measured in the topside ionosphere by the CHAMP spacecraft, as a function of MLT and magnetic latitude. The different populations of Pc3 waves, at equatorial and high latitudes, and on the dayside and the nightside, can clearly be seen from this figure. From Heilig et al. (2007)

In space, statistical surveys of in situ measurements of Pc3 waves have been performed using the data sets of various spacecraft. Multiple studies have consistently shown that equatorial compressional Pc3 wave power peaks in the pre-noon sector and decreases towards the nightside, consistent with the waves originating in the foreshock (Takahashi and Anderson 1992; Cao et al. 1994; Heilig et al. 2007; Balasis et al. 2015; Murphy et al. 2020). There is one notable exception in the study by Lessard et al. (1999), which finds compressional Pc3 wave occurrence peaking in the afternoon sector. As discussed earlier in this Section, this may be due to non-Parker spiral IMF orientations during the studied intervals. Despite the decrease in wave energy towards the nightside, Pc3 waves of foreshock origin have been observed to propagate all the way to the midnight sector (Ponomarenko et al. 2010; Takahashi et al. 2016; Villante and Tiberi 2016; Yagova et al. 2017). Using CHAMP and SWARM measurements in the topside ionosphere, Heilig et al. (2007) and Balasis et al. (2015) have compiled statistical maps of Pc3 wave power as a function of both MLT and magnetic latitude (see Fig. 26). These maps show that the Pc3 wave power decreases from the subsolar point with solar zenith angle, before increasing again at high latitudes, suggesting the existence of multiple entry mechanisms for foreshock waves (see Sect. 5 and 6.1). An enhancement in wave power is also observed in the pre-midnight sector in Fig. 26, which Heilig et al. (2007) ascribe to plasma instabilities associated with ionospheric processes. This feature is not visible in the results of the longer survey of CHAMP data displayed in Balasis et al. (2015) likely because their analysis focused on a more quiet period. Balasis et al. (2015) also reported a puzzling enhancement of compressional Pc3 wave energy in the South Atlantic Anomaly region, which they attributed to the low magnitude of the geomagnetic field therein and the resulting Alfvén velocity within this region.

On the ground, numerous studies have investigated the spatial and occurrence distribution and frequency characteristics of Pc3 waves. At mid and low latitudes, their properties are consistent with fundamental FLR, and Pc3 wave power peaks in the pre-noon region, extending to the morning and afternoon regions (Tanaka et al. 2004). At higher latitudes ($56^{\circ}$–$76^{ \circ}$), Howard and Menk (2005) find that the occurrence of FLR observed on the ground peaks similarly between 07 and 13 MLT. Pc3 waves at these latitudes correspond to harmonics of the local FLR, as the fundamental mode typically falls in the Pc5 range at high latitudes. Furthermore, ground-based observations have shown that Pc3 waves can also be observed at locations magnetically connected to the cusps (e.g. Chugunova et al. 2006; Pilipenko et al. 2008; Liu et al. 2008) and the polar caps (Chugunova et al. 2004, 2006; Pilipenko et al. 2008; De Lauretis et al. 2010; Villante et al. 2011; Francia et al. 2012; Regi et al. 2013, 2014), as discussed in Sect. 6.1.

Pc3 waves in the magnetosphere can also be measured using the Super Dual Auroral Radar Network (SuperDARN), a global network of high frequency radars that has been designed primarily with the aim to study ionospheric plasma convection (Chisham et al. 2007). Most studies of ULF waves using SuperDARN have focused on Pc5 waves, since the common mode of operation of SuperDARN involves azimuthal sweeps with a cadence of 1 min (e.g. Sakaguchi et al. 2012; Bland et al. 2014); however there have also been studies of ULF waves in the Pc3 range that are based on the high time resolution measurements of SuperDARN (e.g. Matsuoka et al. 2002; Shi et al. 2018), which is also known as the “THEMIS” mode, with a rate of $\sim~6~\mathrm{s}$. Matsuoka et al. (2002) reported a clear peak in wave power at cusp latitudes, with a steep decrease towards higher latitudes. The statistical survey by Shi et al. (2018) shows that Pc3 (and Pc4) waves occur predominantly at midlatitudes on the nightside and at high latitudes on the duskside, with very few events at polar latitudes; contrary to that, Pc5 waves are observed predominantly at high latitudes. At midlatitudes, the occurence of Pc3 (and Pc4) waves was found to peak at premidnight and to increase at equinox, during southward IMF conditions and during times of increased geomagnetic disturbances. We note that this statistical survey based on SuperDARN data did not cover the dayside, due to insufficient observation time or lack of ionospheric backscatter (Shi et al. 2018).

### Pc3 Waves and Magnetoseismology

ULF waves generated in the ion foreshock play a central role in a technique known as magnetoseismology. This technique uses the frequencies of toroidal standing Alfvén waves to estimate the plasma mass density based on the fact that the frequencies depend on the Alfvén velocity and thus on the mass density (Menk and Waters 2013). Magnetoseismology is valuable given the difficulty in determining the mass density using particle experiments. In magnetoseismology, we consider each field line to execute eigenmode oscillations with unique frequencies and field line mode structures and use these wave properties to estimate the mass density for each field line. For magnetospheric regions where we have good models of the background magnetic field (i.e., most of the dayside magnetosphere and the near-Earth portion of the nightside magnetosphere), we can solve an appropriate standing wave equation (Singer et al. 1981) to relate the frequencies to the mass density. Note that we also need to specify how the density varies along the field line to define the Alfvén velocity along the entire field line (see below how this can be inferred). On the ground, toroidal wave frequencies are determined using data from pairs of magnetometers with a small latitudinal separation. The relative phase and amplitude ratio between the wave signals from the two locations lead to unambiguous determination of the toroidal wave frequency (Baransky et al. 1985; Waters et al. 1991). In the magnetosphere, dynamic spectra of the electric field and/or magnetic field measured by a single spacecraft usually exhibit narrow spectral peaks, from which accurate determination of toroidal wave frequencies is possible (Takahashi et al. 2006).

Because toroidal waves are excited by fast mode waves through the field line resonance mechanism (Hasegawa et al. 1983; Kivelson and Southwood 1986), the intensity and frequency of the toroidal waves depend on the fast mode waves. In general, more information on the mass density is gained if the toroidal waves are stronger (easier detection) and contain more harmonics. Spacecraft observations have demonstrated that fast mode waves of foreshock origin, usually spanning the Pc3 to Pc4 bands (7–100 mHz), fill a large portion of the dayside magnetosphere unless the IMF is nearly perpendicular to the Sun-Earth line (Greenstadt et al. 1980).

**Fig. 27:**
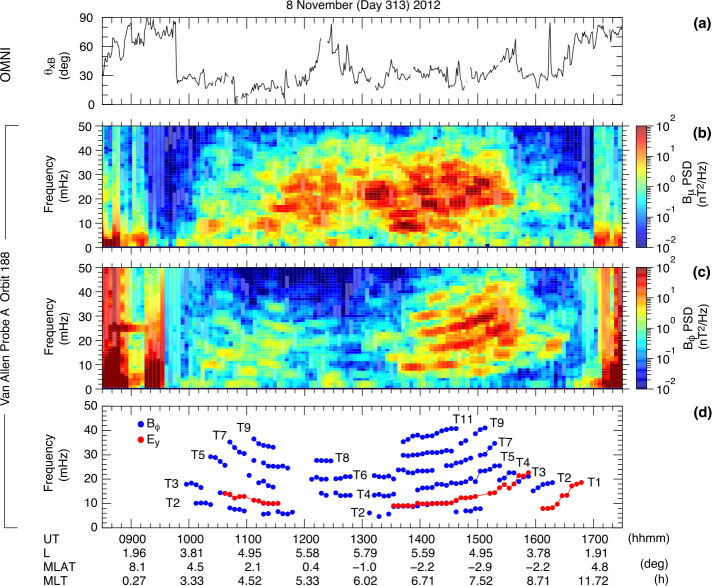
Example of multiharmonic toroidal waves observed by Van Allen Probe A. (a) IMF cone angle according to the solar wind OMNI data. (b) Dynamic spectra of the magnetic field compressional component at Van Allen Probe A. (c) Dynamic spectra of the magnetic field azimuthal component. (d) Frequencies of toroidal waves determined from the magnetic field spectra (blue) and electric field spectra (red). The labels T1, T2,.. indicate the harmonic mode number. The spacecraft location is given at the bottom. This figure combines panels selected from Figs. 3, 4, and 6 of Takahashi et al. (2015), reproduced with permission from Takahashi et al. (2015), copyright by the AGU

Figure 27 shows toroidal waves excited at multiple harmonics spanning the fundamental (T1) through the eleventh (T11). In this example, the IMF cone angle was lower than 45^∘^ most of the time (panel (a)), and this condition clearly led to elevated intensity of foreshock ULF waves and the subsequent broadband enhancement of compressional magnetic field oscillations in the magnetosphere (panel (b)) and their coupling to numerous toroidal harmonics (panels (c) and (d)). Observations like this enable successful execution of magnetoseismology in much of the dayside magnetosphere (Takahashi et al. 2010; Min et al. 2013; Del Corpo et al. 2020). On some occasions, fast mode waves of dayside origin propagate to the midnight sector and couple to toroidal waves (Takahashi et al. 2020). By including such cases, we can construct mass density models covering all local times (Denton et al. 2022).

When toroidal waves are excited at multiple harmonics, the frequencies and the field line mode structure of the waves can be used to infer the mass density distribution along the field line. Typically, one assumes that the mass density ($\rho $) varies along the magnetic field line in the simple form $\rho = \rho _{\mathrm{eq}}(r_{0}/r)^{\alpha}$ (Cummings et al. 1969), where $r_{0}$ is equatorial distance to the field line, $r$ is distance to a point on the field line, $\rho _{\mathrm{eq}}$ is the equatorial mass density and $\alpha $ is the mass density index. The latter two are free parameters to be determined from observation. $\alpha $ specifies how the mass density varies with magnetic latitude. Since there are two free parameters in this model, we need to obtain two or more pieces of information to determine these parameters. In one approach, the information comes from the frequency ratios between harmonics (Takahashi and Denton 2007). In another approach, the information comes from the location of the nodes of the harmonics (Takahashi and Denton 2021). In either approach, $\alpha $ is found to be $\sim 1$, indicating that the mass density decreases slowly from the equator toward the ionosphere along the field line.

## Impact of Pc3 Waves on the Inner Magnetosphere Dynamics and Ionosphere

While the previous section discussed the propagation of Pc3 waves through the magnetosphere, the coupling between different wave modes and their transmission into the ionosphere, we will now focus on the impact of these waves on their environment. There are relatively few studies of the influence of Pc3 waves on the inner magnetospheric and ionospheric plasmas, possibly because of the more limited role they play in magnetospheric dynamics, compared to waves in the Pc5 frequency range. We review here studies showing clear evidence of the impact of Pc3 waves on their environment, and also discuss other possible ways in which these waves could affect the surrounding plasma which have not been confirmed yet.

### Pc3 Modulation of Wave Activity in the Inner Magnetosphere

Pc3 waves have been shown to modulate the generation and growth of higher frequency waves in the inner magnetosphere (Mursula et al. 1997; Rasinkangas and Mursula 1998; Motoba et al. 2019). Mursula et al. (1997) and Rasinkangas and Mursula (1998) analysed a series of bursts of electromagnetic ion-cyclotron (EMIC) waves observed in the inner magnetosphere by the Viking spacecraft and on the ground. During this event, the EMIC wave packet periodicity was about 43 s, which corresponded well with the period of Pc3 waves observed simultaneously by ground-based magnetometers. At the same time, the IMP-8 spacecraft measured ULF waves at similar periods upstream of the quasi-parallel bow shock, suggesting that the foreshock was the source of the magnetospheric Pc3 wave activity. Rasinkangas and Mursula (1998) proposed that Pc3 fluctuations modulate the equatorial growth rate of EMIC waves, in changing periodically the plasma parameters. Even slight changes in those parameters can lead to significant variations of the growth rate (Gail 1990). Similar modulations of EMIC waves at Pc3 frequencies were also observed by the DEMETER spacecraft in the upper ionosphere during geomagnetic storms (Parrot et al. 2006).

In addition to EMIC waves, compressional Pc3 waves can also affect whistler wave activity in the magnetosphere. The modulation of whistler waves by ULF waves was first described in the theoretical work by Coroniti and Kennel (1970), who proposed that ULF waves modulate the distribution of electrons that are in resonance with the whistler waves. This in turn causes a periodic variation of the whistler growth rate. The whistler wave modulation by ULF waves has been extensively confirmed in spacecraft observations, both for Pc3 waves (Morrison 1990; Motoba et al. 2019) and for Pc4–5 fluctuations (e.g. Li et al. 2011, 2022).

**Fig. 28:**
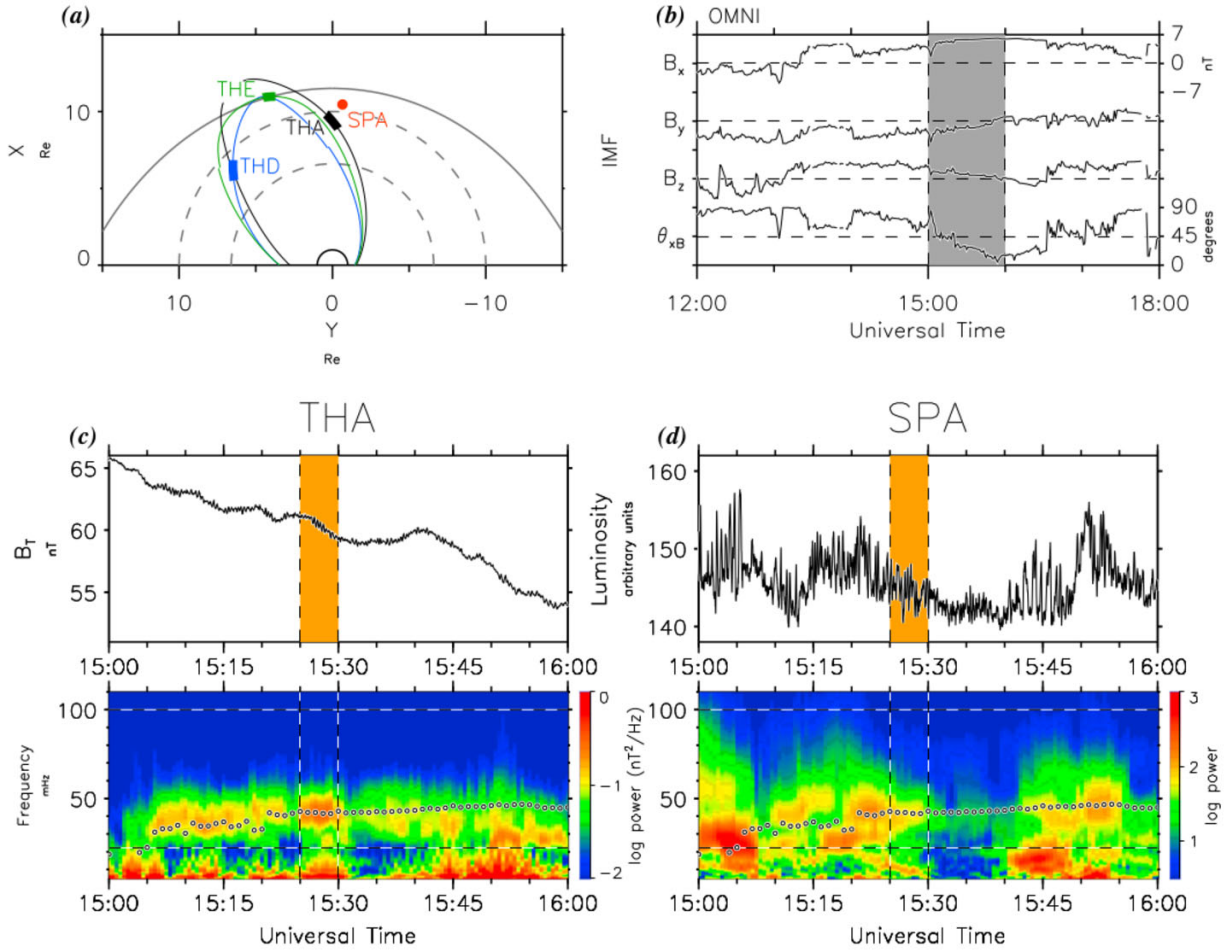
Simultaneous observations of compressional Pc3 waves in the dayside outer magnetosphere and daytime auroral pulsations on the ground. (a) Location of three THEMIS probes and the projection of South Pole Station (SPA) onto the equatorial plane. (b) IMF components and cone angle upstream of the Earth’s bow shock from the OMNI data set. (c) Magnetic field strength measured by the THEMIS-A spacecraft and its associated dynamic power spectrum. (d) Auroral luminosity measured at SPA and its associated dynamic power spectrum. The white circles on the dynamic power spectra correspond to the expected frequency for the foreshock waves, estimated using the Takahashi et al. (1984) formula. Figure reproduced with permission from Motoba et al. (2019), copyright by the AGU

Compressional Pc3 waves are often associated with daytime auroral pulsations, observed equatorward of the cusp, in which the luminosity of the dayside aurora is modulated with a period in the Pc3 range (Wu and Rosenberg 1992; Engebretson et al. 1994b; Vorobjev et al. 1999; Motoba et al. 2017, 2019). An example of simultaneous observations of Pc3 waves and daytime auroral pulsations is shown in Fig. 28. These periodic changes in auroral intensity are due to variations in the precipitating electron fluxes. They in turn can be caused by the modulation of the chorus whistler-mode wave power by compressional Pc3 waves, as chorus waves are an important source of electron precipitation through pitch-angle scattering. Recent works have also shown that compressional ULF waves can act as direct drivers of electron precipitation through the modulation of the loss cone (Brito et al. 2015; Rae et al. 2018). We note that these studies focused on compressional waves in the Pc4 and Pc5 ranges, but in principle, compressional Pc3 waves in the outer magnetosphere could directly drive electron precipitation through the same process. We note that while some events of daytime auroral pulsations were clearly observed in conjunction with foreshock waves (e.g. Engebretson et al. 1990; Motoba et al. 2019), the statistical survey by Vorobjev et al. (1999) showed a larger occurrence rate at cone angles $\theta _{\mathrm{Bx}} > 45^{\circ}$, suggesting that foreshock waves are not the only source of these auroral pulsations. These dayside auroral pulsations occur mostly during geomagnetically quiet times, and their origin remains unclear (Vorobjev et al. 1999).

### Resonant Interactions Between Radiation Belt Electrons and Pc3 Waves

ULF waves are known to play a major role in electron acceleration within Earth’s radiation belts, due to resonant interaction between the waves and the particles (e.g. Zong et al. 2017). However, for this resonance to occur, the wave properties must fulfill some conditions, in particular regarding their frequency which must correspond to a multiple of one of the electron characteristic frequencies. Particles in drift-resonance with a given ULF wave with frequency $\omega $ satisfy the following relation:

2 $$ \omega = m \omega _{\mathrm{d}} $$

where $\omega _{\mathrm{d}}$ is the drift frequency of the electrons and $m$ the azimuthal mode number. Drift-bounce resonance occurs when the following relation is fulfilled:

3 $$ \omega = m \omega _{\mathrm{d}} + l \omega _{\mathrm{b}} $$

where $\omega _{\mathrm{b}}$ is the electron bounce frequency and $l$ a longitudinal mode number of the wave.

Pc3 waves have periods ranging from 10 to 45 seconds. The drift periods of radiation belt electrons around Earth decrease with increasing energy and with increasing distance from the Earth (i.e. with increasing $L$ parameter), and are typically of the order of several hundreds of seconds for relativistic electrons. They are therefore considerably longer than Pc3 periods and their fundamental resonance occurs only with Pc5 or Pc4 waves that have longer periods. For example, the drift period of 10 MeV electrons at $L=6$ is about 100 seconds. The bounce periods of radiation belt electrons are in turn well below the Pc3 band, from a few tens of a second to a few seconds. They decrease with increasing energy and increase with the increasing distance from the Earth. Pc3 wave periods thus reside in-between the bounce and the drift period of typical radiation belt electrons.

Drift and drift-bounce resonance between Pc3 waves and radiation belt electrons could nevertheless occur at higher wave modes. For example, Pc3 waves with a frequency $\omega \sim 33$ mHz, corresponding to a period of 30 s, could resonate with electrons with a drift frequency $\omega _{\mathrm{d}} \sim 5.5$ mHz if their azimuthal mode number is $m = 6$. However, there are no reports of such high wave mode resonances with magnetospheric Pc3 waves, possibly due to the lower wave power in this frequency band, compared to Pc4–5 frequencies, which limits the impact of those waves on radiation belt electron populations.

**Fig. 29:**
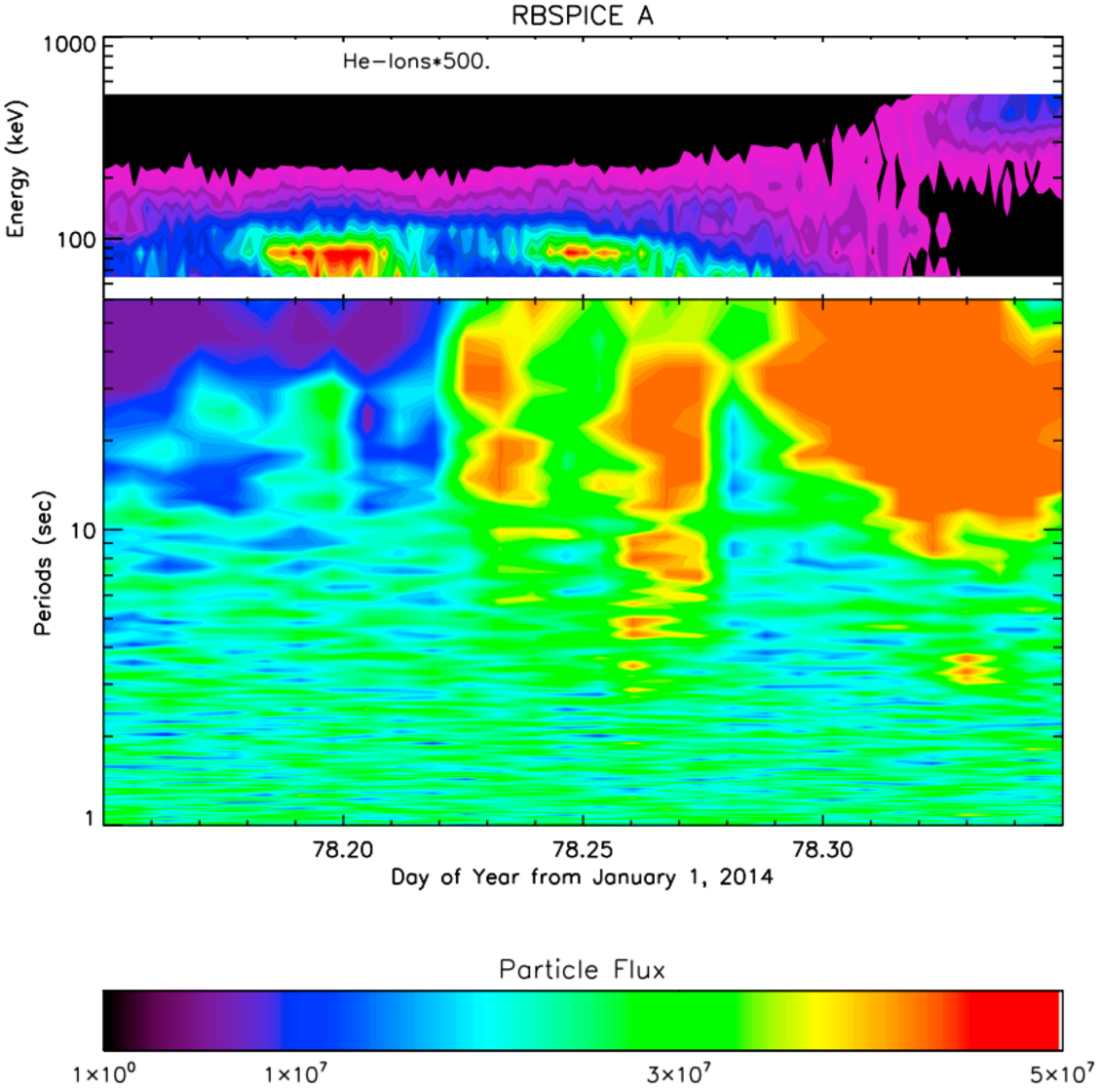
Ion differential fluxes for the $90^{\circ}$ pitch angle helium ions (top) and power spectrum of the parallel magnetic field component (bottom) measured by Van Allen Probe A. A clear anti-correlation can be see between the He ion fluxes and the wave power. From Kim et al. (2017)

Drift-bounce resonance can also take place with other particle populations. Specifically, the period of Pc3 waves matches well with the bounce period of helium ions in the ring current. Kim et al. (2017) report anticorrelated variations of the He^+^ ion fluxes and the compressional Pc3 wave power observed by the Van Allen Probes in the inner magnetosphere (see Fig. 29). This anticorrelation can be interpreted as the signature of drift-bounce resonance between the waves and the He^+^ ions: the ions in resonance with the Pc3 waves are scattered when the Pc3 wave power is enhanced, causing the flux oscillations.

Radial diffusion of radiation belt electrons is generally ascribed to the sole action of Pc4–5 waves. In a recent study, George et al. (2022) estimated the contribution of foreshock-driven Pc3 waves to radial diffusion in a 2D global numerical simulation performed with the Vlasiator model. Using a new methodology they introduced to calculate the Lagrangian derivative in the Roederer $L^{*}$, they found that the diffusion coefficients resulting from Pc3 wave activity alone are several orders of magnitude lower than those obtained in other studies which include Pc4–5 waves. The minor contribution of Pc3 waves to radial diffusion is likely due to the low wave power in this period range, compared to that in the Pc4–5 ranges, as the magnetospheric ULF wave power decreases with frequency as a power law (Gamayunov and Engebretson 2021).

In the ionosphere, Pc3 waves may contribute to Joule heating, although their contribution is likely very small. Using data from the THEMIS satellites, Hartinger et al. (2015) quantified the ULF wave Poynting vector mapped to the ionosphere, as an estimate of the Joule heating rate. The Poynting flux was integrated over frequencies ranging from 3 to 30 mHz, thus including part of the Pc3 range ($22-100$ mHz). They found that while ULF wave contribution to Joule heating is generally quite low, it can become significant in some events, which are typically associated with enhanced geomagnetic activity. They also noted that the ULF wave Poynting vector decreases with increasing frequency, with an energy deposition rate 10 times larger at 5 mHz compared to 10 mHz, suggesting that Pc3 waves contribute to only a very small fraction of the Joule heating. Their contribution may become more significant in geomagnetically quiet times, when Pc5 wave power is low while there may be strong Pc3 wave activity originating from the foreshock.

## Influence of Solar Wind Conditions on Pc3 Wave Activity

The near-Earth space is driven by the continuously changing solar wind. Variations in the solar wind forcing that is exerted onto the magnetosphere affect both the direct wave generation in the foreshock as well as their internal excitation through different mechanisms described in Sects. 2 and 6.

The way the solar wind varies is not completely random. The conditions are determined by the Sun’s global magnetic field that controls the slow and fast stream structures and whose reorganization leads to solar eruptions that can propagate through interplanetary space. The dominant solar wind structures and their properties vary also in concert with the solar activity level through the ∼ 11-year solar cycle.

### Overview of Solar Wind Conditions and Drivers

The most prominent feature of the quiet Sun effects to the heliosphere is the regularly repeating pattern of slow and fast solar wind streams. The fast wind emanates from large coronal holes, while the sources of the slow wind are more varied and less understood (e.g., Cranmer et al. 2017). According to the current understanding, the slow solar wind could come from closed field lines of the coronal streamer belt where it is released via magnetic reconnection, active region outflows and boundaries of coronal holes. During the phases of declining and minimum solar activity in particular, long-lived and large coronal holes dominate near the poles with extensions towards the equator. As a consequence, the fast wind pattern near the ecliptic repeats in 27-day intervals determined by the rotation period of the Sun. The fast solar wind is tenuous and has a relatively high temperature. Its key characterizing signatures are low-frequency Alfvén waves where magnetic and plasma density fluctuations (anti)correlate. Generally, the Alfvénicity and geoffectivity increase with increasing speed of the stream (e.g., Belcher and Davis 1971; Snekvik et al. 2013). The slow wind is denser and cooler than the fast wind and it exhibits large variability in its parameters.

The collision of the fast wind with the slow wind ahead creates compressive regions called stream interaction regions (SIRs) or co-rotating interaction regions (CIRs) to emphasize their often regularly repeating nature with the 27-day solar rotation period (e.g., Richardson 2018). SIRs/CIRs have large-amplitude out-of-ecliptic magnetic field variations that when coupled to high dynamic pressure typically induce weak to moderate magnetospheric storms (e.g., Richardson and Cane 2012; Kilpua et al. 2017b). The following Alfvénic fast stream can keep activity enhanced over periods of several days, in particular causing repeating substorms at high latitudes (Tsurutani and Gonzalez 1987).

Another key component shaping heliospheric conditions are transient Coronal Mass Ejections (CMEs; e.g., Webb and Howard 2012) that originate from gigantic releases of mass and magnetic flux from the Sun. The occurrence rate and properties of CMEs vary in concert with solar activity (e.g., Lamy et al. 2019). Near solar maximum prominent CMEs can occur even several times per day, and they are stronger and faster than during solar minimum conditions (e.g., Yashiro et al. 2004; Gopalswamy et al. 2009). When CMEs are observed directly in the heliosphere they are called Interplanetary CMEs (ICMEs; e.g. Kilpua et al. 2017a). The subset of ICMEs associated with a sustained and smooth rotation of the IMF over a large angle and low plasma $\beta $ are called magnetic clouds (Burlaga et al. 1981). ICMEs, and in particular magnetic clouds, cause irregular and intense geomagnetic storms (e.g., Zhang et al. 2007; Kilpua et al. 2017b; Richardson and Cane 2012).

When ICMEs are sufficiently faster than the preceding solar wind they form an interplanetary shock wave ahead of them. The shock is followed by a turbulent and compressed sheath region (see e.g. Kilpua et al. 2017a, for a review). Shocks and often the sheath as well compress considerably the whole magnetosphere and can excite intense wave activity in the inner magnetosphere. The sheath often contributes significantly to the generation of magnetospheric disturbances and can drive magnetic storms alone even though the following ICME would not be geoeffective (e.g., Huttunen et al. 2005; Kilpua et al. 2017b). Also ICMEs that are not fast enough to drive a shock have disturbed regions ahead if they propagate faster than the preceding solar wind. SIRs/CIRs in turn do not typically have fully developed shocks at the Earth’s orbit but develop them at larger heliospheric distances (e.g., Jian et al. 2006).

### Solar Wind Parameters and Pc3 Wave Generation

#### IMF Orientation

The most critical parameter controlling the occurrence of Pc3 waves is the IMF cone angle $\theta _{\mathrm{Bx}}$, measured between the IMF vector and the Sun-Earth line. The importance of the cone angle stems from its controlling the bow shock geometry, and where its quasi-parallel and quasi-perpendicular regions will be located. As was discussed previously in Sects. 2 and 4, these configurations have very different characteristics in the plasma both upstream and downstream of the shock, and therefore, affect significantly the excitation of 30-second/Pc3 waves.

The average cone angle at Earth is $\sim 45^{\circ}$ corresponding to the Parker spiral orientation, which divides the bow shock into quasi-perpendicular and quasi-parallel regimes. The cone angle can however have any value. Quasi-radial IMF ($B_{x} / B \approx 1$) periods correspond to low cone angle values ($\theta _{\mathrm{Bx}}\leq 30^{\circ}$), during which the subsolar magnetosphere is largely behind a quasi-parallel shock and Pc3 waves are expected to be particularly effectively generated (e.g. Gul’yel’mi 1974; Greenstadt and Olson 1977; Russell et al. 1983; Takahashi et al. 1984). Pi et al. (2014) explored the characteristics and occurrence of radial IMF events using the near-Earth OMNI data base for 1995–2011. The authors found that such periods of nearly radial field are associated with lower magnetic field magnitude, densities and temperatures than on average in the solar wind. Radial IMF (defined in their study as $B_{x} / B \gtrapprox 0.9$) was present 10–15% of the time and the total annual time did not change with the solar activity cycle. The occurrence of long-duration radial IMF events ($> 4$ hours of continuously radial IMF) however was found to vary with the solar cycle with more events observed near minimum and rising phase of solar activity. Long-duration radial IMF periods occur both during solar wind high-speed streams (HSS) and during ICMEs (e.g., Gosling and Skoug 2002). In ICMEs they occur typically in their trailing parts associated with steadily declining solar wind speed and last on average approximately 10 hours (e.g., Neugebauer et al. 1997). They have been associated either to the legs of the ICMEs or to the solar wind flow from transient coronal holes opened by the CME eruption.

**Fig. 30:**
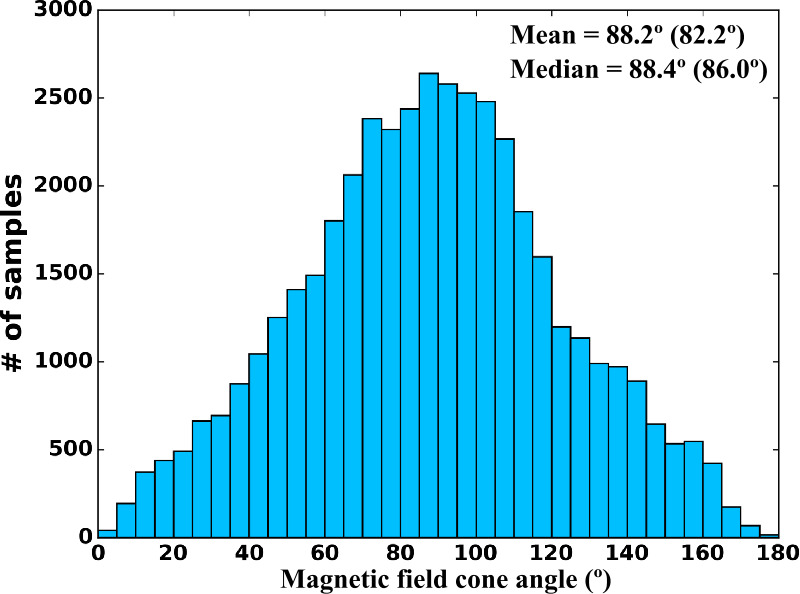
IMF cone angle distribution during magnetic clouds arriving at Earth’s orbit during 2000–2014. Figure reproduced with permission from Turc et al. (2016), copyright by the AGU

Magnetic clouds offer another sustained well-organized behaviour of the IMF as in them the magnetic field direction changes smoothly over a period of about one day. The distribution of cone angles in magnetic clouds has nearly a Gaussian distribution that peaks around $90^{\circ}$ and the dayside bow shock configuration is thus dominantly quasi-perpendicular (see Fig. 30 and Turc et al. 2016). This implies that during magnetic clouds conditions are not always that favorable for the generation of Pc3 waves, but we emphasize that the cone angle can vary greatly between magnetic clouds and also during a given magnetic cloud, and periods with both quasi-parallel and quasi-perpendicular bow shock regimes, as well as periods with an exclusively quasi-parallel bow shock are found.

In compressive solar wind structures, slow–fast stream interaction regions and ICME-driven sheath regions, the direction of the IMF makes large and fast variations. They therefore expose the bow shock under conditions where its configuration can vary in a rapid manner (e.g., Ala-Lahti et al. 2021) that is expected also to have consequences on the Pc3 wave generation. On average, the cone angle tends to be large, above $60^{\circ}$, during sheath regions (Koller et al. 2023). In contrast, its values extend over a much broader range during HSSs and CIRs (Koller et al. 2023), suggesting that these structures may be more favourable for Pc3 wave generation. Villante et al. (1999) have reported Pc3 wave observations at a low-latitude ground station during a HSS. The wave activity peaked at two frequencies, the higher one likely corresponding to local field line resonances. The wave activity at lower frequencies was intermittent, due to the highly-variable IMF orientation, and its frequency varied together with the IMF strength, and thus these waves were identified as transmitted foreshock waves propagating antisunward.

#### IMF Strength

As evidenced already in early works, the frequency of foreshock waves and of Pc3 pulsations on the ground appears to be linearly related to the magnitude of the interplanetary magnetic field (e.g., Saito 1964; Russell and Hoppe 1981; Hoppe and Russell 1982; Takahashi et al. 1984; Troitskaya and Bol’Shakova 1988; Le and Russell 1996; Heilig et al. 2007). This is due to the waves being generated by (anomalous) cyclotron resonance in the foreshock, and their frequency is thus proportional to the ion cyclotron frequency in the foreshock. A number of studies have focused on establishing the relationship between these two parameters, finding that the wave frequency in mHz is given by the IMF strength in nT multiplied by a factor close to 6. Using observations in the foreshocks of Mercury, Venus, Earth and Jupiter, Hoppe and Russell (1982) also showed that the frequency of foreshock waves increases linearly with the IMF magnitude across the solar system as $f [\mathrm{mHz}] = 5.8B_{\mathrm{IMF}}[\mathrm{nT}]$.

**Fig. 31:**
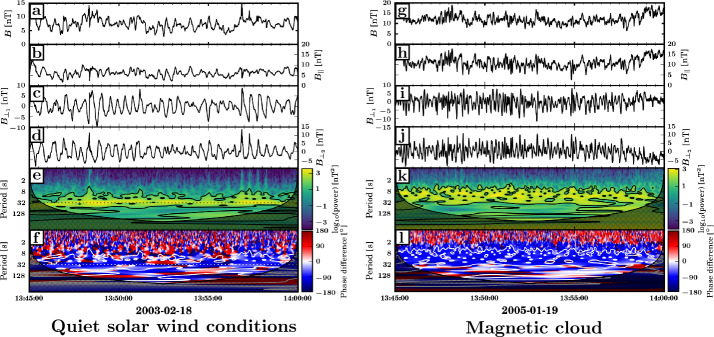
Comparison of foreshock 30-second wave properties during quiet solar wind conditions (left) and magnetic cloud events (right), showing a higher and more variable wave frequency when the IMF strength is enhanced. Figure reproduced with permission from Turc et al. (2019), copyright by the AGU

At Earth, high IMF magnitude conditions occur in particular during ICMEs. While the nominal solar wind IMF strength is about 5 nT, in ICMEs it is typically > 10 nT and can exceed several tens of nT for extended periods of time (e.g., Kilpua et al. 2017a; Gopalswamy et al. 2015). The properties of foreshock 30-second waves during magnetic cloud events have been analysed in Turc et al. (2019) (see Fig. 31). Six events were identified during which the Cluster satellites were located inside the foreshock while the spacecraft separation was suitable to perform detailed multi-spacecraft analysis of the wave properties. As expected, the foreshock wave period was much lower than its typical 30-second value, because of the enhanced magnetic field strength inside magnetic clouds which results in a higher cyclotron frequency. More surprisingly, the study also revealed that the usually quasi-monochromatic wave activity is replaced by a superposition of fast-mode waves at different periods during magnetic clouds. The transverse extent of the wave fronts is also considerably reduced, from 8–$18~R_{\mathrm{E}}$ during quiet solar wind conditions (Archer et al. 2005) to $3~R_{\mathrm{E}}$ on average for magnetic clouds (Turc et al. 2019). These results were consistent with numerical simulations performed with the Vlasiator model (Turc et al. 2018, 2019). This suggests that the unusual solar wind conditions during magnetic clouds tend to disrupt the large-scale structuring of the foreshock wave field.

**Fig. 32:**
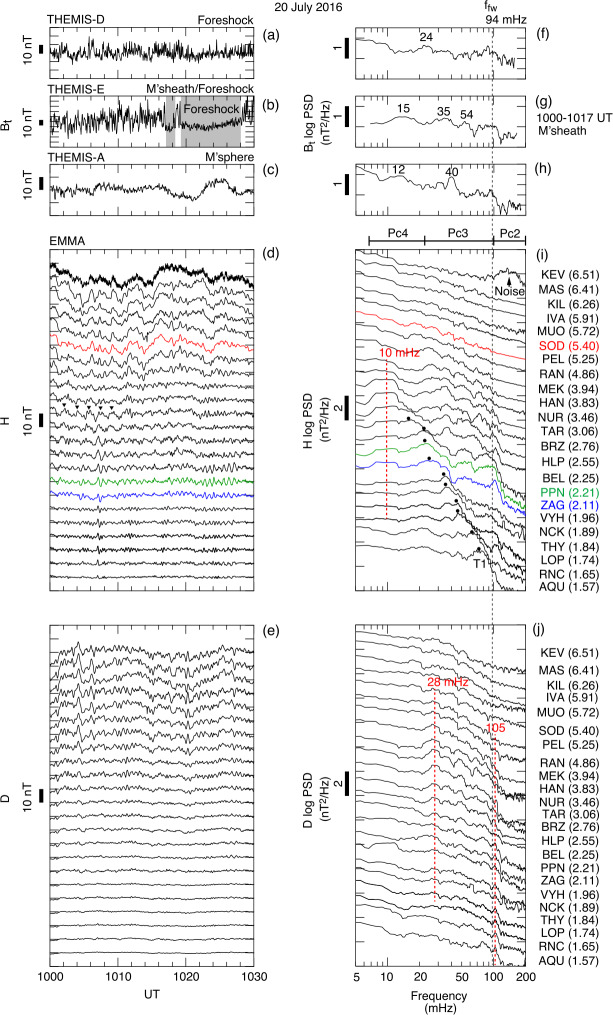
Magnetic field measurements and associated power spectra of the fluctuations from the THEMIS-D (foreshock), THEMIS-E (magnetosheath and foreshock), and THEMIS-A (dayside magnetosphere) satellites, and from magnetometers from the EMMA network (ground). The ground-based measurements strongly differ from the upstream observations, showing in particular no clear peak at the foreshock wave frequency (94 mHz). Figure reproduced with permission from Takahashi et al. (2021), copyright by the AGU

The consequences of these changes in the foreshock wave properties for their Pc3 counterparts inside the magnetosphere was then investigated in Takahashi et al. (2021). The conjunction of spacecraft and ground-based measurements during the magnetic cloud that impacted Earth on 20 July 2016 allowed a detailed study of Pc3 waves from the foreshock to the nightside magnetosphere in these atypical driving conditions. The foreshock wave power was particularly high during this event, likely due to the enhanced solar wind density accompanying the strong magnetic field. Clear signatures of compressional Pc3 fluctuations were observed in the dayside magnetosphere, suggesting wave transmission. However, spacecraft on the nightside did not measure any significant wave power enhancement in this frequency range, whereas foreshock Pc3 waves are known to transmit all the way to the midnight sector during typical solar wind conditions (Takahashi et al. 2016). This was attributed to the shorter spatial scale lengths of the waves in the foreshock, both their wavelength and their transverse extent (Turc et al. 2019), which likely prevents them from penetrating deep into the magnetosphere. Furthermore, it was found that because of the higher frequency of the foreshock waves (90 mHz), the transmitted waves could not couple with fundamental field-line resonances at midlatitudes, as the wave frequency no longer matches the resonant frequencies at these latitudes. As a result, no outstanding oscillations at the foreshock wave frequency could be found in the ground-based measurements (see Fig. 32). The altered foreshock wave properties during magnetic cloud events thus profoundly modify their transmission into the magnetosphere.

#### Other Solar Wind Parameters

**Fig. 33:**
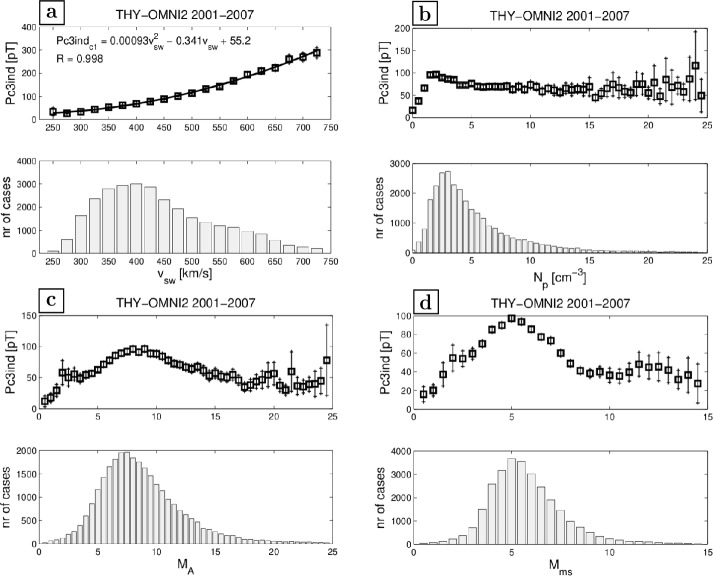
Pc3 wave power observed on the ground between 2001 and 2007, as a function of (a) solar wind velocity, (b) solar wind density, (c) Alfvén and (d) magnetosonic Mach number. Adapted from Heilig et al. (2010)

Heilig et al. (2010) investigated Pc3 geomagnetic fluctuations for 2001–2007 under different solar wind conditions. The solar wind speed had a strong positive correlation with the Pc3 activity (see Fig. 33a). The speed dependency has been demonstrated in other studies as well, (e.g., Saito 1964; Greenstadt et al. 1979; Takahashi et al. 1981; Wolfe 1980; Morrison 1991; Heilig et al. 2007). One suggested explanation is that the enhanced likelihood for the Kelvin-Helmholtz instability to develop at the magnetopause during high speed solar wind could lead to enhanced Pc3 wave power. Another possibility is that higher solar wind speeds could result in larger foreshock wave amplitude, due to the larger shock Mach number (Kovner et al. 1976). The latter is in better agreement with the other observed dependencies of the Pc3 wave power, which suggest that the foreshock is the dominant driver of magnetospheric Pc3 wave activity (although not the only one, as described in Sect. 6). In addition to controlling magnetospheric Pc3 wave power, the solar wind speed can also influence the propagation of these waves towards the nightside magnetosphere. Yagova et al. (2017) showed that Pc3 waves of foreshock origin are observed by ground-based stations on the nightside when the solar wind speed exceeds 500 km/s.

For the solar wind density in turn Heilig et al. (2010) did not find a clear correlation with Pc3 wave occurrence except that Pc3 wave activity was extremely low during periods of very tenuous solar wind despite otherwise favourable upstream conditions such as a low IMF cone angle (Fig. 33b). They suggested that this could be due to the ceasing of the ion-cyclotron resonance that generates upstream ULF waves as there would be too few suprathermal foreshock ions in these conditions, weakening and relaxation of the bow shock, or the solar wind becoming sub-Alfvénic. A similar result was also reported for example by Francia et al. (2013) and Le et al. (2000) who observed that ULF waves on the ground disappeared during very low solar wind density conditions ($< 2~\mathrm{cm}^{-3}$). The authors concluded that this is in favour of the interpretation that Pc3 waves are related to the foreshock waves as a very weak bow shock reflects ions inefficiently. In contrast, the numerical simulations performed in Turc et al. (2022) still showed significant Pc3 wave power both in the foreshock and the dayside magnetosphere despite using a low solar wind density, $1~\mathrm{cm}^{-3}$. The authors suggested that this could be due to the relatively high Mach numbers in their low-density simulations. This would result in a stronger bow shock than typically observed during tenuous solar wind at Earth which is generally accompanied by a low Alfvén Mach number (e.g. Le et al. 2000).

For solar wind dynamic pressure Heilig et al. (2010) in turn found a clear trend of Pc3 wave occurrence increasing with increasing dynamic pressure values. This could be partly related to clear positive correlation with the solar wind speed discussed earlier and partly due to for example strong magnetospheric compression enhancing the wave generation. Heilig et al. (2010) also demonstrated that the occurrence of Pc3 pulsations was clearly highest when the magnetopause was more compressed than when it was located further away from the Earth.

Solar wind density, speed and IMF magnitude determine the solar wind Alfvén Mach number and therefore the strength of the bow shock. The Alfvén Mach number increases with increasing solar wind speed and density, and decreases with increasing IMF magnitude. The generation of Pc3 waves is expected to depend strongly on the shock strength: the stronger the shock, the higher the power of both foreshock waves and Pc3 pulsations. This trend was confirmed in global numerical simulations (Turc et al. 2022), but interestingly, the ground-based measurements analysed in Heilig et al. (2010) show that Pc3 wave power maximises near the average Alfvén and magnetosonic Mach numbers at Earth and then decreases again (see the bottom panels of Fig. 33). Le et al. (2000) investigated the interesting and widely-studied period on 11 May 1999 known as the “*day the solar wind almost disappeared*”. At this time solar wind density was unusually low, resulting in the bow shock moving far from the Earth and becoming very weak because of the very low Mach number. As a consequence, unusually low Pc3–4 wave activity was observed in the magnetosphere.

#### Empirical Relationship Between 30-Second/Pc3 Wave Frequency and Upstream Solar Wind Parameters

To conclude this section, we provide here a list of some of the relationships between the 30-second/Pc3 wave frequency and the upstream solar wind conditions that have been established either empirically or based on theoretical considerations. We note that these different formulas can lead to vastly different predictions for a given set of solar wind parameters (e.g. Turc et al. 2022).

As mentioned above, numerous studies calculated the relationship between the IMF magnitude and the wave frequency in their data set, either based on measurements of Pc3 waves on the ground (Troitskaya et al. 1971)

4 $$ f [\mathrm{mHz}] = 6.15 B_{\mathrm{IMF}}[\mathrm{nT}] $$

or observations of foreshock waves (Russell and Hoppe 1981)

5 $$ f [\mathrm{mHz}] = (5.81 \pm 0.14) B_{\mathrm{IMF}}[\mathrm{nT}]. $$

Based on a theoretical model of particle reflection at the shock, Takahashi et al. (1984) proposed the following equation for Pc3 wave frequency:

6 $$ f [\mathrm{mHz}] = 7.6 B_{\mathrm{IMF}}[\mathrm{nT}] \cos ^{2} \theta _{\mathrm{Bx}}. $$

Le and Russell (1996) analysed observations from the ISEE satellite in the foreshock to obtain an empirical relationship for the wave frequency:

7 $$ f [\mathrm{mHz}] = (0.72 + 4.67\cos \theta _{\mathrm{Bx}}) B_{ \mathrm{IMF}}[\mathrm{nT}]. $$

The following expression was obtained by Heilig et al. (2007) using measurements from the SWARM satellites in the topside ionosphere:

8 $$ f [\mathrm{mHz}] = (0.708 \mathrm{M}_{\mathrm{A}} + 0.64)[ \mathrm{mHz}/\mathrm{nT}]\cdot B_{\mathrm{IMF}}[\mathrm{nT}]. $$

Heilig et al. (2010) employed multiple regression analysis and artificial neural networks to obtain an empirical model of Pc3 wave power based on seven years of data from ground-based magnetometers at a range of latitudes. The best-fit values for their multi-parameter model can be found in Heilig et al. (2010).

## Beyond Earth

### 30-Second Waves in Other Planetary Foreshocks

Foreshocks are a ubiquitous feature of collisionless supercritical quasi-parallel shocks, throughout the heliosphere and beyond. Fast-mode waves generated via the ion-ion beam right hand resonant instability, analogous to the terrestrial 30-second waves, have been observed at most planetary bow shocks in our solar system (e.g. Hoppe and Russell 1982; Russell 1994; Dubinin and Fraenz 2016). The early work by Hoppe and Russell (1982) compares the frequency of these waves at Mercury, Venus, Earth and Jupiter, and found that the nearly linear relationship between the wave frequency and the IMF strength already established at Earth (Russell and Hoppe 1981) extends throughout these different planetary foreshocks, spanning two orders of magnitude in frequency and IMF strength.

At Mercury, the cone angle of the Parker spiral IMF is around 20–30^∘^ (James et al. 2017). The foreshock thus typically extends in front of the dayside magnetosphere in the Hermean system due to this quasi-radial IMF orientation. The local equivalent of 30-second waves have periods between 3–20 s, due to the large IMF strength (Le et al. 2013; Jarvinen et al. 2020b; Romanelli et al. 2020). One crucial difference with Earth’s foreshock is that these waves are observed only sporadically, while the dominant wave mode are whistler waves with frequencies around 2 Hz. Recent statistical studies using data from the MESSENGER mission have estimated the occurrence rate of these 3–20 s-waves: they are observed only $0.5\%$ of the time when the spacecraft is magnetically connected to the bow shock (Romanelli et al. 2020; Romanelli and DiBraccio 2021). This is likely due to the low Alfvén Mach number of the solar wind at Mercury, which typically ranges between 3–6 because of the larger IMF strength closer to the Sun. The influence of the Alfvén Mach number is further supported by the fact that the wave occurrence is larger for lower IMF strength, associated with times when Mercury is found at larger heliocentric distances (Romanelli and DiBraccio 2021). A reduced Alfvén Mach number results in a low density of suprathermal ions, which in turn lowers the growth rate of the waves. The small size of the Hermean environment, providing little time for wave growth, as well as the enhanced variability of the solar wind at Mercury’s orbit, could also affect the wave growth (Le et al. 2013).

The amplitude of foreshock waves tends to be lower at Mercury, with $\delta B/B \sim 0.2$ (Romanelli et al. 2020), whereas $\delta B/B \sim 1$ is commonly observed at Earth (e.g. Hobara et al. 2007). Furthermore, the waves have not been observed in their steepened form, as shocklets, in MESSENGER data (Le et al. 2013). This is again likely due to the low Alfvén Mach number in the solar wind at Mercury.

**Fig. 34:**
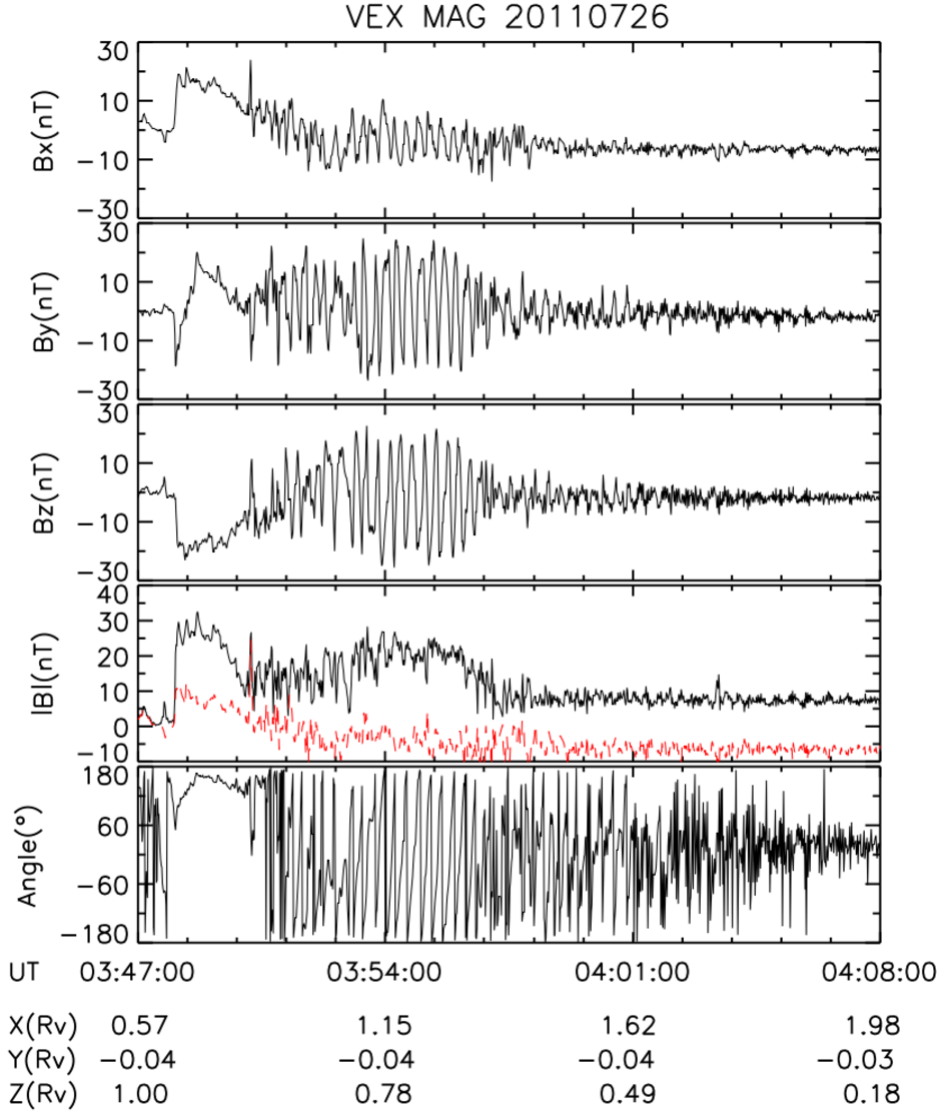
Large-amplitude ULF waves with period of $\sim 20$ s in the foreshock and magnetosheath of Venus observed by the Venus Express satellite on 26 July 2011. Figure reproduced with permission from Shan et al. (2014), copyright by the AGU

Observations in and numerical simulations of the Cytherean environment have revealed that its foreshock is populated with quasi-monochromatic fast-magnetosonic waves with periods around 20–30 s, very similar to their terrestrial counterparts (see Fig. 34 and Shan et al. 2014, 2016; Omidi et al. 2017; Shan et al. 2018; Jarvinen et al. 2020a). Furthermore, waves at similar frequencies can be found in the magnetosheath of Venus (Luhmann et al. 1983; Shan et al. 2014). Analysis of the wave properties showed that the waves retained their spacecraft frame frequency, propagation direction, and polarisation in the magnetosheath, as well as their fast-mode nature (Shan et al. 2014). In contrast with Mercury, foreshock transient structures such as SLAMSs and SHFAs (see Sect. 2) have been identified in spacecraft data, suggesting that foreshock waves can grow and steepen in the Cytherean environment (Collinson et al. 2012, 2017). Because of the small size of Venus’ induced magnetosphere, the bow shock subsolar point is located at an altitude of about 2000 km (Zhang et al. 2008; Shan et al. 2015). As a result, foreshock waves can have a large impact on this planetary environment. Numerical simulations suggest in particular that foreshock fast-mode waves can modulate oxygen ion escape at Venus (Jarvinen et al. 2020a).

The foreshock ULF wave field at Mars is dominated by fast-mode waves at the local ion gyrofrequency and whistler waves (e.g. Dubinin and Fraenz 2016, and references therein). This pronounced difference with Earth’s foreshock is due in particular to the presence of pick-up ions upstream of Mars’ induced magnetosphere, which are thought to be the source of the waves at the proton gyrofrequency. To our knowledge, there is only one recent report of observations of fast-magnetosonic waves in the Martian foreshock at a period consistent with a generation due to ion beam instabilities, and thus comparable to the terrestrial 30-second waves (Shan et al. 2020). The numerical simulations by Jarvinen et al. (2022) also show foreshock compressional waves at frequencies lower than the proton gyrofrequency, which could be caused by foreshock ion beams rather than pickup ions. Independently of their source, fast-magnetosonic waves transmitting from the foreshock into the downstream can have a significant impact on the Martian environment. They can induce fast-magnetosonic waves in the ionosphere, which heat and energise the plasma in this region, and can lead to ionospheric particle escape (Collinson et al. 2018; Fowler et al. 2018, 2021).

### ULF Waves Upstream and Downstream of Interplanetary Shocks

Interplanetary shocks form when a faster flow overtakes slower plasma in the heliosphere, with a difference in speed larger than the local fast magnetosonic speed (see Sect. 8.1). These shocks typically precede solar wind high-speed streams and interplanetary coronal mass ejections (e.g. Kilpua et al. 2015) and play an important role in the generation of energetic particles in the solar system (Lee et al. 2012). Because of the large scales of their drivers, their radius of curvature is much larger than that of planetary bow shocks, and interplanetary shocks can be considered planar over large distances. The majority of interplanetary shocks observed at Earth’s orbit have a quasi-perpendicular geometry, while only about 25% of the events are in a quasi-parallel configuration (Kilpua et al. 2015; Blanco-Cano et al. 2016). Interplanetary shocks tend to be much weaker than Earth’s bow shock: their magnetosonic Mach number is generally below 2.5 (Kajdič et al. 2012; Kilpua et al. 2015; Blanco-Cano et al. 2016), while the typical Mach number for Earth’s bow shock is about 8–10 (e.g. Ma et al. 2020). Observations have even shown examples of subcritical interplanetary shocks, at which energy dissipation takes place through resistivity only (Kajdič et al. 2012).

**Fig. 35:**
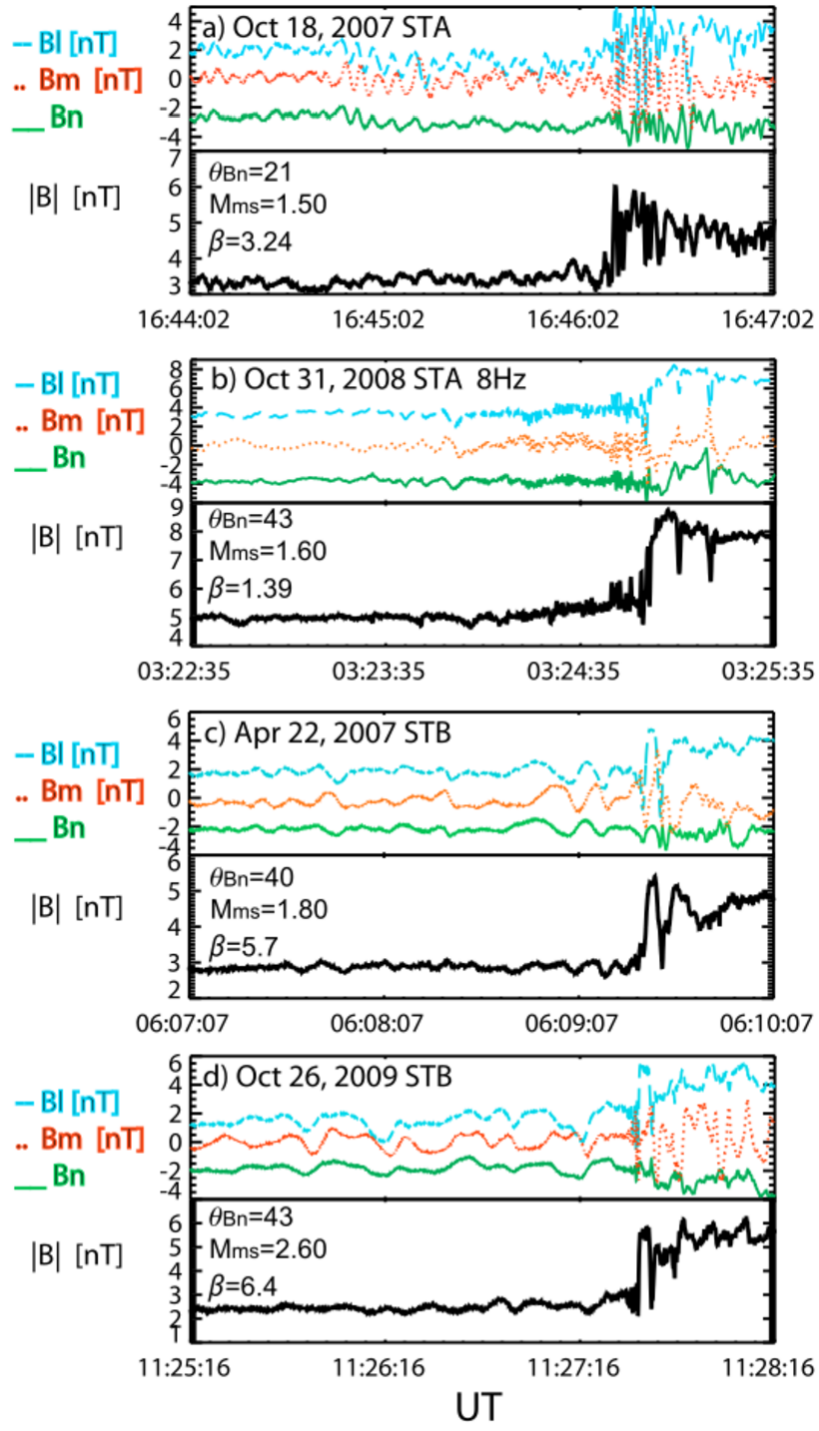
Examples of magnetic field measurements upstream and downstream of quasi-parallel interplanetary shocks observed by the STEREO spacecraft, showing prominent, mostly transverse ULF waves in the upstream of the shocks. Figure reproduced with permission from Blanco-Cano et al. (2016), copyright by the AGU

Quasi-parallel supercritical interplanetary shocks can be preceded by an extended foreshock populated with suprathermal ions and/or ULF waves (Tsurutani et al. 1983; Kajdič et al. 2012; Blanco-Cano et al. 2016). These ULF waves have properties similar to the terrestrial 30-second waves: they are mostly transverse, circularly- or elliptically-polarised right-handed waves, with their wavevector nearly aligned with the ambient magnetic field, and their properties are consistent with a generation through cyclotron resonance with the backstreaming ions (Tsurutani et al. 1983). Four examples of ULF waves upstream of quasi-parallel interplanetary shocks are shown in Fig. 35. Contrary to Earth’s foreshock, however, the wave amplitudes remain small and wave steepening has only been observed in rare instances, with just a few reports of compressive shocklets in the foreshock of interplanetary shocks (Lucek and Balogh 1997; Wilson et al. 2009; Trotta et al. 2023). This is likely due to the low Mach numbers associated with these shocks. Interestingly, ULF waves are also found upstream of more oblique shocks, with $45^{\circ} < \theta _{\mathrm{Bn}} < 65^{\circ}$, together with field-aligned beams which are typically found upstream of this portion of the shock (e.g. Meziane et al. 2005). In contrast, at Earth, these waves are confined to magnetic field lines threading the quasi-parallel shock, due to the finite growth rate of the waves, and are not observed in conjunction with field-aligned beams (Blanco-Cano et al. 2009; Turc et al. 2018). This difference may be due to the large radius of curvature of interplanetary shocks, which results in a different large-scale organisation of interplanetary shock foreshocks (Blanco-Cano et al. 2016). This could also be due to the interplanetary shock configuration having changed from quasi-parallel to perpendicular shortly before the time of observation (Kajdič et al. 2012).

The downstream region of quasi-parallel interplanetary shocks is populated by compressive ULF waves with a combination of different polarisations (Kajdič et al. 2012; Blanco-Cano et al. 2016). It is probable that these waves are a mixture of transmitted waves from the upstream and locally-generated fluctuations, as in Earth’s magnetosheath. Mirror modes and Alfvén ion cyclotron waves have been in particular identified in sheath regions preceding ICMEs, with occurrence rates peaking just downstream of the interplanetary shock (Ala-Lahti et al. 2018, 2019). Whether and how wave transmission takes place through the interplanetary shock remains however an open question.

### Field Line Resonances at Other Planets

Field line resonances are thought to be a common feature of planetary magnetospheres in our solar system. While some of their properties such as their typical frequencies can differ due to the distinct plasma parameters associated with each planet’s environment, the basic physical process at play remains the same: shear Alfvén waves propagating along a field line form a standing wave between two conjugate points in the ionosphere. While less studied than their terrestrial counterparts due to scarcer data, the characterisation of field line resonances at other planets can bring new insights into this process in other plasma regimes and for different magnetospheric plasma distributions.

At Jupiter and Saturn, the periods of field line resonances are much longer than at Earth, between 10–100 min instead of 1–10 min, due to the large size of the giant planets’ magnetospheres (Delamere 2016; Lysak 2022). The first observations of possible standing waves in the Jovian system were reported as early on as the Voyager 2 flyby (Khurana and Kivelson 1989). They revealed magnetic pulsations with periods around 10–20 min, although it was concluded that their properties were likely not consistent with a coupling between compressional and Alfvén mode, which is typically associated with field line resonances at Earth (Khurana and Kivelson 1989). Quasi-periodic magnetic perturbations have then been repeatedly identified in satellite measurements collected in Jupiter’s environment (Delamere 2016). Models of wave propagation in the Jovian magnetosphere suggest that the majority of these quasi-periodic pulsations can be ascribed to standing Alfvén waves (Manners et al. 2018; Manners and Masters 2019; Lysak and Song 2020).

Similarly, the magnetosphere of Saturn harbours persistent quasi-periodic waves with a period of about one hour (Palmaerts et al. 2016; Yates et al. 2016). Modelling works show that their properties are consistent with field-line resonances (Yates et al. 2016; Rusaitis et al. 2021). To our knowledge, there are no reports of field line resonances or standing Alfvén waves at Uranus and Saturn, but in theory these waves could develop in the magnetospheres of the ice giants as well.

Another crucial difference between the giant planets and Earth that is relevant for the development of field line resonances is the distribution of plasma inside the magnetosphere. At Jupiter, the absence of a plasmasphere and the presence of a dense plasma torus results in a different distribution of the Alfvén speed as a function of planetocentric distance (Lysak 2022). Furthermore, the magnetic field lines are nearly dipolar only in the inner magnetosphere, while a magnetodisc of distorted field lines is found in the outer magnetosphere, which again can affect the characteristics of field line resonances (Delamere 2016).

The main sources of ULF fluctuations in the giant planets’ magnetospheres are moon–magnetosphere interactions, radial transport and flux circulation in the magnetodisc, and external drivers such as the Kelvin-Helmholtz instability and pressure variations in the solar wind (Delamere 2016). The relative importance of these internal and external sources remains unknown. In a recent statistical survey combining data from multiple missions, Manners and Masters (2020) investigated the global distribution of ULF waves in the equatorial magnetosphere of Jupiter. Their results showed that the waves occur most often in the outer magnetosphere on the dayside and dusk flank, likely corresponding to external generation processes, but that the wave energy is largest in the inner magnetosphere (Manners and Masters 2020).

We note that foreshock waves are not mentioned as a possible source of ULF perturbations at the giant planets in the review by Delamere (2016). This could be due to the large cone angle of the Parker spiral IMF at the orbits of Jupiter and Saturn, which is about $80^{\circ}$ (Jackman and Arridge 2011). As a result, the foreshock influences only sporadically the dayside magnetosphere as it would lie on average much further down on the dawn flank than at Earth.

**Fig. 36:**
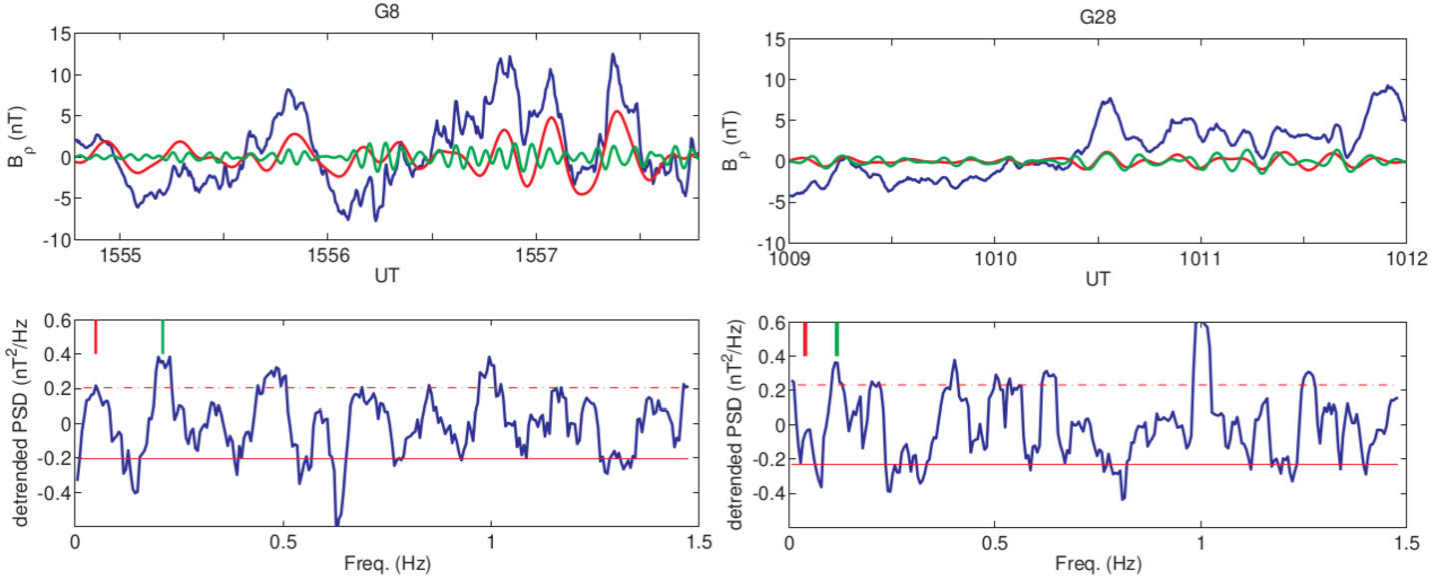
Field-line resonances in the magnetosphere of Ganymede, observed during the G8 and the G28 Galileo flybys. The top panels show one of the transverse magnetic field components and the bottom panels the associated power spectra. The red and green bars in the bottom panels indicate the first and second harmonics and the horizontal lines depict the 95% confidence level. From Volwerk et al. (2013)

Smaller magnetospheres can also harbour field line resonances. Measurements taken during the Galileo flybys of Jupiter’s largest moon Ganymede showed signatures of standing waves with multiple harmonics, at frequencies ranging between 60 mHz and 1 Hz (Volwerk et al. 1999, 2013). This suggests that the mini-magnetosphere of Ganymede, embedded within Jupiter’s magnetic field, can sustain its own field line resonances (see Fig. 36 and Volwerk et al. 1999, 2013).

In the Hermean environment, the Mariner 10 observations first showed evidence of narrowband waves with properties consistent with field line resonances (Russell 1989). More recently, James et al. (2016) and James et al. (2019) conducted statistical surveys of ULF waves at Mercury using data collected by the MESSENGER mission, and showed that transverse wave power, which could partly correspond to field line resonances, is mostly found on the dayside. Pronounced north-south asymmetries were found in the displacements of the field lines due to the planet’s offset dipole (James et al. 2019). One event with a change in wave polarisation which could reflect the coupling between a compressional wave and a standing Alfvén wave is described in James et al. (2016).

An interesting difference between field line resonances at Mercury and other planets is that Mercury does not possess an ionosphere, and thus the boundary conditions for the resonance to be established are unclear (James et al. 2016). It has been suggested that the metallic core could play the role of the ionosphere (Russell 1989), or that the low conductivity could result in an open-ended boundary (Glassmeier et al. 2004). Furthermore, the frequency of field line resonances is of the order of the ion gyrofrequency at Mercury, due to the small scale of the Hermean system (Glassmeier et al. 2004). As a result, MHD does not apply and the waves must be studied in a kinetic framework. The presence of multiple ion species in Mercury’s magnetosphere also complicates matters when studying the generation of ULF waves in this system.

ULF wave activity at Mercury is thought to be mainly driven by the Kelvin-Helmholtz instability, solar wind buffeting of the magnetosphere, and the formation of flux transfer events at the magnetopause (James et al. 2016). As described in Sect. 9.1, the Hermean foreshock houses significant ULF wave activity, which could be transmitted into the downstream. In a recent study, Kallio et al. (2022) report ULF waves on closed field lines in a global hybrid-PiC model of Mercury’s environment. As these waves are found on the same flank as the foreshock, the authors speculate that foreshock waves could be a source of the waves identified inside the magnetosphere. Upcoming observations from the BepiColombo mission (Benkhoff et al. 2021), which is scheduled to arrive at Mercury towards the end of 2025, could shed new light on ULF wave activity and wave transmission in the Hermean environment.

## Outlook

As demonstrated in this review article, 30-second/Pc3 waves are the topic of ongoing research in Earth’s magnetosphere and beyond. In this section, we provide a list of outstanding questions associated with these waves, their transmission, their impact on their environment and their role in solar wind-magnetospheric coupling. We then discuss how recent and upcoming developments in our observational and numerical capabilities could help addressing these unresolved issues. Synergies between global simulations and multi-point spacecraft observations offer a particularly promising avenue for breakthroughs in our understanding of these waves.

### Outstanding Questions

#### Impact of Foreshock Waves on Solar Wind-Magnetosphere Coupling

The foreshock plays an integral part in the processing of the solar wind through the bow shock, initiating the slowdown and deflection of its flow (Cao et al. 2009; Gutynska et al. 2020), and contributing to particle acceleration to suprathermal energies. As discussed throughout this review, foreshock waves can transmit into the shock’s downstream regions, causing enhanced wave activity in the magnetosheath and inside the magnetosphere. As a result, pronounced differences are observed in the properties of the quasi-perpendicular and quasi-parallel magnetosheaths, leading to marked dawn-dusk asymmetries (e.g. Dimmock et al. 2014, 2016, 2017). Furthermore, the foreshock is a major source of transient structures, such as foreshock transients and magnetosheath jets, which can cause local and global disturbances in Earth’s magnetosphere (Plaschke et al. 2018; Zhang and Zong 2020; Zhang et al. 2022). Since this review focuses on foreshock waves, we will concentrate on open questions pertaining to those waves, and discuss two ways in which they can alter solar wind-magnetosphere coupling in influence processes responsible for plasma entry into the magnetosphere.

**What is the impact of foreshock waves on plasma entry into the magnetosphere associated with the Kelvin-Helmholtz instability?**

A major open question for solar wind-magnetosphere coupling is the source of the observed dawn-dusk asymmetry in the plasma sheet ions. Ions in the dawnside plasma sheet are 30-40% hotter and denser than in the duskside, suggesting an asymmetry in the entry mechanism (Wing et al. 2005). On average, the ion temperature in the dawnside magnetosheath is 15% larger compared to the duskside magnetosheath, likely due to the influence of the quasi-parallel shock and its associated foreshock (Dimmock et al. 2015a). Enhanced turbulence downstream of the quasi-parallel shock could in particular cause this increased heating. Another mechanism that likely contributes to the plasma sheet asymmetry is the Kelvin-Helmholtz instability. Numerical works suggest that when the IMF has a component in the equatorial plane, this instability develops preferentially downstream of the quasi-parallel shock (Nykyri 2013). Furthermore, it has been shown that larger velocity fluctuations are observed in the quasi-parallel magnetosheath (Dimmock et al. 2016), and that these would result in enhanced plasma transport through the magnetopause (Nykyri et al. 2017). These works were however done using local simulations of the KHI, based on magnetosheath parameters from global MHD simulations. We are therefore still lacking a comprehensive view connecting the foreshock to its impact on magnetosheath waves and on the development of the KHI. Future studies using global kinetic simulations could shed further light on this topic and enable quantifying the impact of foreshock waves on plasma transport associated with the KHI.

**What is the impact of foreshock waves on magnetopause reconnection?**

Foreshock-associated waves and turbulence transmitting into the magnetosheath can change significantly the magnetic field orientation in this region. This is particularly important when the IMF has a strong radial component, as this can cause reversals of the magnetic field North-South component, or generate a southward magnetic field component when there was previously none in the upstream solar wind. In turn, this modified IMF can trigger bursts of magnetic reconnection at the dayside magnetopause. Evidence of this phenomenon has been shown in the recent numerical study by Chen et al. (2021a). Furthermore, foreshock waves and turbulence can affect the steadiness of reconnection, leading to bursty reconnection during steady solar wind driving (Zou et al. 2022). Further work is required to fully understand and quantify the impact of foreshock waves on dayside reconnection.

#### 30-Second Waves in the Foreshock and the Magnetosheath

**What is the spatial variability of 30-second wave properties inside the foreshock?**

While the influence of the solar wind conditions on some of the 30-second wave properties, in particular their frequency, has been the topic of multiple studies (e.g. Hoppe and Russell 1982; Le and Russell 1996; Hsieh and Shue 2013; Turc et al. 2019), very few investigations have focused on their spatial variations inside the foreshock. For a given set of solar wind conditions, it is generally assumed that the 30-second wave properties are homogeneous and are solely controlled by the solar wind parameters. However, recent numerical simulations providing a global view of the foreshock wave properties during steady solar wind conditions suggest that the wave period and amplitude vary across the foreshock (Turc et al. 2022). Further work is required to quantify this variability and identify the causes of these variations, which may be related for example to inhomogeneties in the foreshock ion population.

It would be extremely valuable to complement these numerical works with observational studies. However, conducting such an investigation using spacecraft measurements is challenging, as data collected at different positions in the foreshock are also associated with different solar wind conditions. The extended data base of foreshock observations provided by long-lived missions such as Cluster could however facilitate the study of spatial variations inside the foreshock. Case studies with multi-point measurements at relatively large separation distances could also be compared with numerical results. Global maps of the wave frequency, compressibility or transverse extent throughout the foreshock would provide crucial information regarding the large-scale organisation of the foreshock wave field, and help in characterising its impact on the shock and the downstream regions.

**What is the relationship between 30-second waves, shocklets and SLAMS?**

Foreshock quasi-sinusoidal 30-second waves are known to become more compressional and steepen as they approach the bow shock. Large-amplitude solitary structures such as shocklets and short large-amplitude magnetic structures (SLAMS) are commonly observed near the shock front, but their formation mechanisms and their connection to 30-second waves are still unclear. Furthermore, even the separate classification of SLAMS and shocklets is still debated, as discussed in Zhang et al. (2022). The distinct properties of SLAMS and shocklets, including their typical scale sizes, have led some authors to suggest that these structures have different origins (e.g. Wilson 2016), while others argue that they are the manifestation of two different stages of the non-linear evolution of foreshock ULF waves (Zhang et al. 2022). The recent work by Chen et al. (2021b) suggests that SLAMS formation may be related to 3-second waves, rather than to 30-second waves. This would be consistent with the narrower spatial scales associated with SLAMS (Lucek et al. 2004), whereas the spatial scale sizes of shocklets are comparable to those of 30-second waves, of the order of a few Earth radii (Le and Russell 1994; Zhang et al. 2022). Further research is thus needed to fully understand the non-linear evolution of 30-second foreshock waves.

**What is the impact of transmitted foreshock waves on the magnetosheath, in particular on the other magnetosheath wave modes?**

The magnetosheath is populated with a variety of ULF waves, in particular mirror modes and Alfvén ion cyclotron waves which are locally generated by temperature anisotropies in this region (see Sect. 4). Turc et al. (2023) discuss that transmitted foreshock waves can only be observed clearly in satellite measurements in the subsolar magnetosheath, while they are hidden by other magnetosheath waves with stronger wave power further on the flanks. Whether this is merely due to the superposition of the different wave modes or to wave-wave interactions in the magnetosheath remains however unclear.

**How do 30-second waves influence the formation of magnetosheath high-speed jets?**

Magnetosheath high-speed jets are localised dynamic pressure enhancements in the magnetosheath, which can hit the magnetopause and cause disturbances inside the magnetosphere (e.g. Plaschke et al. 2018). These jets are predominantly found downstream of the quasi-parallel shock, and their main proposed formation mechanisms are related to foreshock ULF waves. Suni et al. (2021) have shown that even weakly compressive structures in the foreshock could result in magnetosheath jet formation. The foreshock compressive structures identified by Suni et al. (2021) are likely closely linked with 30-second waves. Another well-established formation scenario for magnetosheath jets is via shock ripples, through which the solar wind is deflected with little slowdown as it crosses into the downstream (Hietala et al. 2009). Foreshock ULF waves can control the spatial scale size and the amplitude of shock ripples (Kajdič et al. 2021). Therefore, 30-second waves may play an important role in the formation of magnetosheath high-speed jets, for example in controlling some of their properties such as their spatial scales or the magnitude of the dynamic pressure enhancements, which remains to be explored.

#### Wave Transmission

**Does the transmission of foreshock waves through the bow shock depend on the geometry of the interaction?**

We summarised in Sects. 3 and 4 some recent results pertaining to foreshock wave transmission through the bow shock, proposing a new pathway to connect 30-second waves with magnetospheric Pc3 waves (Turc et al. 2023). This study however focused on a specific geometry, with the IMF making a $\sim 30^{\circ}$ cone angle with the Sun-Earth line and the foreshock edge lying rather close to the subsolar bow shock. Future studies should confirm whether wave transmission takes place through the same pathway for other IMF orientations as well. It would also be interesting to identify whether there are critical cone angle values for the waves to reach the dayside magnetosphere, and to investigate whether the IMF direction affects the properties of the magnetosheath counterparts of foreshock waves.

**How do the fast-mode pulses propagating through the magnetosheath generate quasi-monochromatic waves inside the magnetosphere?**

Foreshock waves can form coherent wave fronts with a transverse extent spanning up to several Earth radii, but their coherence scale can also be much smaller than the scale size of the shock, especially just upstream of the shock front (Archer et al. 2005; Turc et al. 2019; Takahashi et al. 2021; Kajdič et al. 2021). This implies that different parts of the bow shock will be interacting with different phase fronts at a given time, and will generate fast-mode pulses traversing the magnetosheath in a desynchronised manner. One may thus expect to observe a broadband spectrum of waves in the dayside magnetosphere, caused by the superposition of the staggered fast-mode pulses at the foreshock wave frequency. However, observations indicate that magnetospheric Pc3 waves are quasi-monochromatic, suggesting that the finite transverse extent of the foreshock wave fronts does not affect wave transmission. One possibility could be that only those fast-mode pulses crossing the subsolar magnetosheath are relevant for wave transmission, while foreshock waves impacting other parts of the shock do not reach the magnetosphere. Global 3D numerical simulations of the dayside magnetosphere will be crucial to address this question, as they will provide a global view of wave transmission from the foreshock into the magnetosphere and will enable comparing the wave properties in different parts of the foreshock, magnetosheath and dayside magnetosphere.

**How does foreshock wave transmission take place in other planetary environments?**

As described in Sect. 9, observations and numerical simulations of the plasma environments of Venus and Mars indicate that foreshock fast-mode waves can transmit into the magnetosheath (e.g. Shan et al. 2014; Jarvinen et al. 2022). The wave properties inside the magnetosheath however differ from those reported at Earth in Turc et al. (2023), with in particular the waves retaining their circular polarisation in the downstream (Shan et al. 2014). This suggests that a different process may be responsible for wave transmission through the shock at Venus and Mars. This could be due to different wave properties in the foreshock, such as a reduced compressibility of the waves, or to the smaller scale of these planetary plasma environments, resulting in the bow shock curvature becoming comparable with the ion gyroradius. These effects would be particularly prominent at Mars, where the bow shock standoff distance is of the order of the solar wind ion gyroradius. The presence of pick-up ions at these weakly-magnetised planets may also influence the processes at work in foreshock wave generation and transmission. Further studies are needed to understand the mechanisms underlying foreshock wave transmission in these planetary environments.

#### Magnetospheric Pc3 Waves

**What are the relative contributions of the equatorial and high-latitude entry mechanisms to Pc3 wave activity in the dayside magnetosphere?**

As described in Sect. 5, several mechanisms have been proposed for the entry of compressional waves into the dayside magnetosphere. Near the equator, fast-mode waves can propagate through the magnetopause considered as a tangential discontinuity, while at high latitudes the waves can either penetrate through the cusp or modulate electron precipitation. Disentangling the contribution of these various entry processes to the global distribution of Pc3 waves inside the magnetosphere is however challenging, as the waves can propagate away from their sources and couple with field-line resonances. The statistical analysis of Pc3 wave measurements in the topside ionosphere conducted by Heilig et al. (2007) clearly shows peaks in compressional Pc3 wave power both near the subsolar point and at high latitudes. This could suggest that the contribution of both equatorial and high-latitude entry may be equivalent on average, although the wave power enhancement at high latitudes is ascribed to auroral activity by Heilig et al. (2007). More studies are needed to quantify the relative contributions of these different pathways for Pc3 wave transmission, and how they are related to foreshock wave properties and upstream solar wind conditions. 3D global hybrid simulations could provide a useful tool to separate the relative contributions of equatorial and high-latitude entry.

**What is the impact of Pc3 waves in the inner magnetosphere?**

The influence of Pc3 waves on inner magnetospheric dynamics has received little attention so far. A handful of studies suggest that Pc3 waves may play an important role in modulating the amplitude of EMIC and chorus waves, which in turn affect particle precipitation into the upper atmosphere (Mursula et al. 1997; Motoba et al. 2019). There have also been observations of drift-bounce resonance between Pc3 waves and He^+^ ions from the ring current, causing particle scattering (Kim et al. 2017). In theory, Pc3 waves with high azimuthal wave numbers could be in drift or drift-bounce resonance with radiation belt electrons, but there is no observational evidence of such resonant interactions. This might be due to the wave power of Pc3 waves being too low compared to that of Pc4–5 waves, which have a dominant contribution to radiation belt dynamics, or to their typical wave numbers being too low to fulfill the resonance condition. Quantifying the azimuthal wave numbers of Pc3 waves in the inner magnetosphere would provide crucial clues regarding their possible impact on radiation belt electrons.

#### Impact of Solar Wind Conditions

**What is the impact of extreme solar wind conditions on foreshock and Pc3 wave properties?**

A number of studies have investigated the relationship between Pc3 wave properties and solar wind parameters (see Sect. 8), and shown a positive correlation between Pc3 wave power and solar wind speed (Heilig et al. 2007, 2010), dynamic pressure (Heilig et al. 2010), and an increase in wave power at lower cone angle (Heilig et al. 2010). The influence of the solar wind density on the wave amplitude observed on the ground seems to be limited, except for extremely low values ($< 2~\mathrm{cm}^{-3}$), which lead to Pc3 wave activity vanishing despite otherwise favourable solar wind conditions (Le et al. 2000; Heilig et al. 2010; Francia et al. 2013). However, numerical simulations with a solar wind density of $1~\mathrm{cm}^{-3}$ can show significant compressional wave power in the dayside magnetosphere (Turc et al. 2022). One possible explanation for this apparent discrepancy is that those simulations were associated with relatively high Alfvén Mach numbers, whereas intervals of low solar wind density at Earth occur generally conjointly with low Alfvén Mach numbers, for which less particle reflection takes place at the shock and foreshock waves cannot grow to large amplitudes. This issue could be resolved by revisiting these intervals of low solar wind density and analysing conjointly the influence of the Alfvén Mach number. Identifying whether there is a threshold in one or a combination of solar wind parameters below which Pc3 wave activity disappears in the dayside magnetosphere would provide additional clues regarding their transmission mechanism. At the other end of the spectrum of solar wind conditions, it would also be interesting to characterise how large the amplitude of foreshock and Pc3 waves can grow, and for which values of the upstream parameters, in order to determine when these waves would have the largest impact on dayside magnetospheric dynamics.

Another topic which has been little investigated is the transverse extent of the foreshock wave fronts. It has been shown that a larger IMF strength reduces their transverse extent (Turc et al. 2019), but whether other solar wind parameters also affect this remains unknown. Also, the fact that the waves have both a shorter wavelength and a shorter transverse extent at higher IMF magnitudes may imply that these two parameters are related, which requires further investigation.

**How are the properties of foreshock and Pc3 waves modified during large-scale solar wind structures?**

The recent works by Turc et al. (2019) and Takahashi et al. (2021) have shown that for high IMF strength, the foreshock waves display a broader spectral peak, compared to the narrowband waves typically observed in the foreshock. Furthermore, the transverse extent of the foreshock waves is reduced and the transmitted waves inside the magnetosphere are strongly attenuated. Observations suggest that these waves cannot couple with field line resonances because of their higher frequency, which does not match the fundamental frequencies of the inner magnetosphere field lines (Takahashi et al. 2021). This study was however limited to a single event, with an extended interval of radial IMF combined with a high IMF strength. Future studies including more events associated with enhanced IMF magnitude are needed to gain further insight into these unusual wave transmission conditions.

Solar wind parameters favourable to foreshock wave generation and transmission are rare during ICMEs. These solar wind structures tend to have larger cone angles, both during their sheath region and their magnetic ejecta (Turc et al. 2016; Koller et al. 2023). Consequently, the foreshock extends further down on the bow shock flanks and little Pc3 activity is expected to arise in the dayside magnetosphere. Long-duration radial IMF intervals can be observed in the trailing edge of ICMEs (Watari et al. 2005), which could provide suitable events to study foreshock wave transmission for extended periods of time. We note however that the IMF strength tends to be close to its typical values in the trailing part of ICMEs and thus the solar wind conditions may not differ strongly from the quiet solar wind.

Solar wind high-speed streams are associated with a broader range of IMF cone angle values (Koller et al. 2023), and thus can provide conditions more favourable for foreshock and Pc3 wave generation than ICMEs. The properties of these waves during high-speed streams have however not been investigated to date, likely because the IMF direction tends to be strongly variable in these structures, which makes it difficult to capture accurately the wave properties.

### Present Observational Capabilities and Future Opportunities for Multi-Point Observations of Foreshock and Pc3 Waves

As detailed in the previous sections, multi-point observations are crucial to accurately determine the wave characteristics in the different regions of near-Earth space. Cluster’s four-spacecraft measurements have been key to retrieving the wave properties in the plasma rest frame, thus allowing an unambiguous identification of the wave modes both in the foreshock and the magnetosheath (e.g. Eastwood et al. 2002, 2005a; Narita et al. 2006; Narita and Glassmeier 2006; Hobara et al. 2007). Inside the magnetosphere, the combined analysis of data sets from multiple spacecraft, multiple ground-based observatories, or combinations of both space and ground measurements, provide critical information regarding the global distribution of Pc3 wave power and the propagation direction of the waves (e.g. Tanaka et al. 2004; Takahashi et al. 2016; Kim et al. 2020). Furthermore, multi-point observations in different regions of near-Earth space are essential to track the waves as they propagate from the foreshock to the inner magnetosphere.

**Fig. 37:**
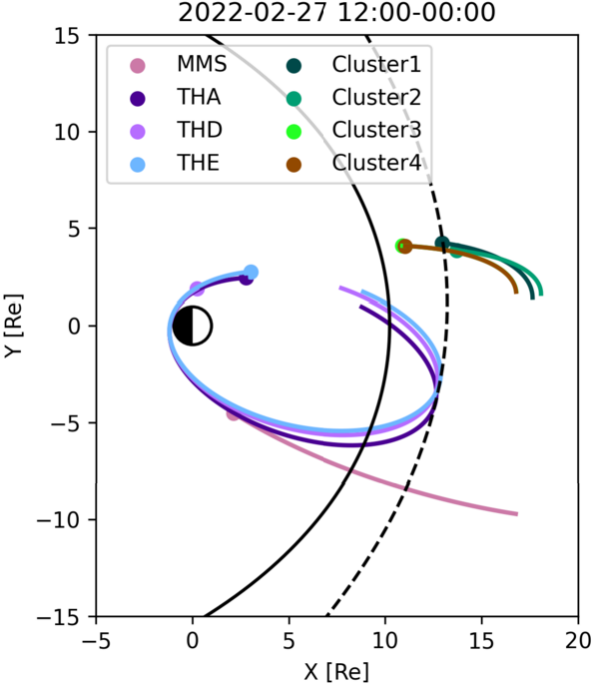
Dayside conjunction of the Cluster, MMS and THEMIS missions on 27 February 2022. The approximate magnetopause (solid black line) and bow shock (dashed black line) positions are determined using the Shue et al. (1998) and Merka et al. (2005) models, respectively, using the average solar wind parameters between 12:00 and 23:59 UT on 27 Feb 2022

A few detailed case studies combining observations from multiple vantage points throughout near-Earth space have been carried out, providing important insight into wave transmission (Clausen et al. 2009; Francia et al. 2012; Takahashi et al. 2016, 2021). However, the magnetosheath is seldom included in those studies, for lack of simultaneous measurements in this region. Spacecraft conjunctions, such as the dayside conjunctions of the Cluster, THEMIS and MMS missions in early 2022, can provide suitable configurations to study foreshock wave transmission into the magnetosheath and the magnetosphere. An example of such conjunction which occurred on 27 February 2022 is displayed in Fig. 37. These measurements can be complemented with data from ground-based observatories and from spacecraft orbiting in the inner magnetosphere such as GOES and Arase, in order to cover the entire chain from the foreshock to the ground.

In the coming years, new missions could provide further opportunities to refine our understanding of foreshock/Pc3 wave generation and transmission. NASA’s HelioSwarm (Klein et al. 2023) will comprise a fleet of nine spacecraft, with interspacecraft separations ranging from the fluid to the sub-ion scales. Although the mission’s science objectives are focused on solar wind turbulence, the spacecraft orbit will cross regularly through the foreshock and the magnetosheath, thus providing new multi-point measurements in these regions. The cross-scale spacecraft constellation could allow studying the global structure of the foreshock wave field, which at the moment can mostly be investigated using global numerical simulations. Similarly, the Plasma Observatory mission concept proposed to ESA’s M7 call would provide seven-point measurements at distances ranging from the ion to the fluid scales (Retinò et al. 2022). The foreshock and the magnetosheath are among the key science regions for Plasma Observatory, where the spacecraft will dwell longer. This will enable high-fidelity measurements of foreshock and magnetosheath waves, and possibly both simultaneously, for extended periods of time. These future constellation missions will thus greatly expand our observational capabilities to investigate the transmission of foreshock waves through the outer regions of near-Earth space. At other planets, the dual-spacecraft Bepi-Colombo mission, which will orbit Mercury from December 2025 onwards (Benkhoff et al. 2021), will provide a great opportunity to study ULF wave generation and transmission in a small magnetosphere under strong solar wind driving. Comparing and contrasting the processes at play at Earth and at Mercury will further our understanding of these phenomena.

### Towards More Realistic Simulations of Foreshock and Pc3 Waves in Near-Earth Space

Using numerical simulations to study foreshock/Pc3 waves and their transmission across near-Earth space poses numerous challenges. One of the major hurdles is that the waves are triggered by an ion-kinetic process, which thus calls for simulations going beyond an MHD approach. Hybrid (kinetic ions and fluid electrons) or fully kinetic simulations are however much more computationally expensive than MHD simulations. Because of this, most numerical works pertaining to foreshock/Pc3 waves have focused on their generation and their properties in the foreshock, first using local shock simulations (e.g. Krauss-Varban and Omidi 1991; Krauss-Varban 1995), and later global setups (e.g. Omidi et al. 2005; Blanco-Cano et al. 2006). Recent works have investigated the wave transmission into the magnetosheath and the dayside magnetosphere (Takahashi et al. 2021; Turc et al. 2022, 2023), but the simulations used in these studies were limited to two dimensions (2D) in ordinary space, and thus cannot describe the coupling of compressional waves with field-line resonances inside the magnetosphere. Takahashi et al. (2021) and Turc et al. (2022) focused on compressional Pc3 waves caused by foreshock waves in the outer magnetosphere.

As computing resources increase, global 3D hybrid simulations providing a self-consistent description of foreshock and Pc3 waves in their global context progressively become achievable. The impact of fast-mode disturbances originating from the foreshock on the magnetopause and the magnetosphere has been studied using global 3D hybrid-PIC simulations, revealing mode conversion into kinetic Alfvén waves at the magnetopause, and the generation of field line resonances and field-aligned currents inside the magnetosphere (Shi et al. 2013, 2017, 2021). Whether those foreshock disturbances actually correspond to 30-second waves is however unclear because the scaled ion inertial length, set to $0.1~R_{\mathrm{E}}$ in these simulations, alters the relative scale sizes between foreshock structures and waves and the global scales of the magnetosphere. This makes the comparison with near-Earth space measurements challenging. More work is needed to obtain the global picture of foreshock/Pc3 wave transmission from numerical simulations.

Another important challenge for these numerical works is to include an accurate description of the inner magnetospheric plasma, which is crucial for the coupling of the incoming compressional waves with field line resonances. Specifically, the density distribution inside the magnetosphere, and thus the Alfvén velocity profile, is central to properly simulate field line resonances (e.g. Lysak 2022). The magnetospheric plasma parameters are also important for wave transmission though the magnetopause, as MHD theory predicts that their transmission rate depends on the wave velocities both upstream and downstream of the boundary (McKenzie 1970).

Finally, we note that the ion-kinetic approach is not only crucial to describe foreshock wave generation, but also provides a more accurate description of other regions of near-Earth space, which may play a role in wave transmission from the foreshock to the inner magnetosphere. In the magnetosheath, there may be interaction with other magnetosheath waves, such as the mirror modes and the Alfvén ion cyclotron waves which dominate wave activity in this region. The growth of both of these modes is caused by a temperature anisotropy in the plasma, and thus require an anisotropic plasma description to be properly simulated. The inclusion of kinetic effects is expected to result in mode conversion into kinetic Alfvén waves at the magnetopause (Johnson and Cheng 1997; Johnson et al. 2001; Shi et al. 2013, 2017). Also, finite Larmor radius effects can arise in kinetic simulations, in particular at the magnetopause, which might affect wave transmission across this boundary. Investigating the propagation of foreshock/Pc3 waves across near-Earth space using global ion-kinetic simulations could thus bring new insight into the transmission of other externally-generated ULF waves, which is typically studied using MHD simulations because their sources do not involve ion-kinetic processes (Claudepierre et al. 2010; Ellington et al. 2016; Archer et al. 2022).

### Synergy Between Simulations and Observations

There is a tremendous potential in studies combining both observations and numerical simulations to further our understanding of foreshock and Pc3 waves in near-Earth space. This has however been hindered by the challenges inherent to simulating these waves in their global context, as detailed just above. Recent works carried out in the framework of our ISSI team clearly demonstrate the potential of this synergy, as simulations provide the global context to localised measurements and facilitate event identification (Takahashi et al. 2021; Turc et al. 2023). As numerical simulations of foreshock and Pc3 waves become more realistic, including the global magnetosphere in 3D, with correct scale separation and with a better description of the inner magnetospheric plasma, this will open up new avenues for detailed model-data comparisons, making use of the wealth of multi-point observations available in near-Earth space.

## Summary

Foreshock 30-second waves were discovered in the 1960s, and their connection with Pc3 wave activity inside the magnetosphere was established in the same decade. Extensive research has been carried out since then, aiming at understanding the generation mechanisms and the properties of those waves, their transmission across near-Earth space and their relation with upstream solar wind conditions. Yet despite these considerable efforts, many issues remain unsolved, as evidenced by the open questions we have collected in Sect. 10. More advanced numerical models and data from new missions are required to address these questions. Foreshock/Pc3 waves travel from the outermost regions of Earth’s magnetic environment all the way down to our planet’s surface, spanning multiple regions of geospace which are typically studied separately. We hope that this review fulfills its goal of providing a comprehensive view of the physics of these waves on their earthward journey, tying together the processes taking place in distinct regions of near-Earth space, and that it may inspire new researchers to embark on the study of this fascinating topic.
